# Latitude or altitude as the future refugium? A case for the future of forests in Asia Minor and its surroundings

**DOI:** 10.1002/ece3.11131

**Published:** 2024-04-12

**Authors:** Bikem Ekberzade, Omer Yetemen, Yasemin Ezber, Omer Lutfi Sen, Hasan Nuzhet Dalfes

**Affiliations:** ^1^ Eurasia Institute of Earth Sciences, Istanbul Technical University Istanbul Turkey; ^2^ Istanbul Turkey

**Keywords:** biodiversity, climate change, dynamic vegetation modeling, ecological refugia, LPJ‐GUESS, plant traits, vegetation range shifts

## Abstract

At the current juncture with climate change, centennial projections of species distributions in biodiversity hotspots, using dynamic vegetation models may provide vital insight into conservation efforts. This study aims to answer: (1) if climate change progresses under a business‐as‐usual scenario of anthropogenic emissions for this century, how may the forest ranges be affected? (2) will there be potential regional extinctions of the taxa simulated? (3) may any site emerge as a potential refugium? Study Area: Anatolian Peninsula and its surroundings, longitudes 24–50° E, latitudes 33–46° N. Time Period: 1961‐2100. Major Taxa Studied: 25 woody species and a C3 grass‐type. Method: Keeping a spatial window large enough to track potential changes in the vegetation range and composition especially in the mountain ranges within the study area, we parameterized a process‐based regional‐to‐global dynamic vegetation model (LPJ‐GUESS v 4.1), forced it with ERA5‐Land reanalysis for the historical period, and five different bias‐corrected centennial global circulation model (GCM) datasets under SSP5‐8.5, and simulated the dynamic responses of key forest species. Bivariate spatio‐temporal maps from the simulation results were constructed for final analysis. Results: A significant increase in woody taxa biomass for the majority of our study area, towards the end of the century was simulated, where temperate taxa with high tolerance for drought and a wider range of temperatures took dominance. The mountain ranges in our study area stood out as critical potential refugia for cold favoring species. There were no regional extinctions of taxa, however, important changes in areal dominance and potential future forest composition were simulated. Main Conclusions: Our simulation results suggest a high potential for future forest cover in our study region by the end of the century under a high emissions scenario, sans human presence, with important changes in vegetation composition, including encroachment of grasslands ecosystems by woody taxa.

## Graphical abstract

Determining potential eco‐refugia or any future range shift for woody taxa requires a clear assessment of what an uncertain future may hold for these potential climate change mitigators in their native ranges. By means of a process‐based dynamic vegetation model and high resolution climate data, we simulated the potential forest ranges for the Anatolian Peninsula and its surroundings, sans human presence, for 25 key forest species and a grass‐type, keeping a close watch on the mountain systems to assess their potential as eco‐refugia for sensitive taxa. Our simulation results suggest important centennial changes in vegetation composition in favor of the temperate taxa for our study area, a potential woody species encroachment into steppe ecosystems, and an altitudinal prioritization for potential migration of the boreal taxa rather than a latitudinal one for a temporal window of 140 years.

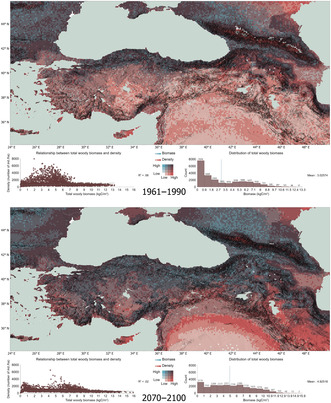

## 1 INTRODUCTION

Paleo records indicate that the Anatolian Peninsula acted as an efficient land bridge, allowing the migration of taxa mainly between Africa‐Arabia and Eurasia for millennia (Korkmaz et al., 2014; Rögl, 1999; Xafis et al., 2021). Although the vegetation composition in this important seed bank went through changes over geological time as did the climate of the peninsula (Akkemik et al., 2023; Denk et al., 2017), a significant number of present‐day taxa are descendants of a lineage that survived major climatic shifts prior (Bouchal et al., 2018; Güner et al., 2017). Despite the heavy human footprint on its ecosystems, the region is still home to a critical but fragmented forest mass which is under threat by changes in climate triggering potential shifts in drought patterns, untimely frost, and changes in the frequency and severity of wildfires (IPCC, 2021). Since the future bears much uncertainty, modeling exercises with different high‐resolution datasets under probable emission scenarios are one way of preparing for what is yet to come. The use of process‐based dynamic vegetation models in predicting possible long‐term outcomes of forest composition, production, density, and distribution (Hickler et al., 2004; Morales et al., 2007; Usman et al., 2021) by forcing these models with different climate datasets (Wu et al., 2017) is one way to estimate potential ecological responses of taxa to concepts such as competition and disturbance (D'Onofrio et al., 2018; Gritti et al., 2006). These experiments may contribute to minimize uncertainties by taking into consideration spatial heterogeneity, thus improving forecast accuracy (Frnda et al., 2022; Matsueda & Palmer, 2011).

The Anatolian Peninsula and its immediate surroundings include a series of critical biodiversity hotspots for woody taxa, including old growth forests (Gerber et al., 2015; Kurdoğlu & Şen, 2005; Moreno‐Sanchez & Sayadyan, 2005; Nakhutsrishvili, 1998; Naqinezhad et al., 2012). These natural seed banks are vital hubs which may become critical sources for future conservation, rewilding, and restoration efforts. The heterogeneous topography of the region may also continue to act in part as potential future refugia for taxa – as it has in the past (Bilgin, 2011; Homami Totmaj et al., 2021; Médail & Diadema, 2009). However, the diverse terrestrial ecosystems of this unique region are under continuous pressure from anthropogenic interferences, including severe ecosystem fragmentation due to land use and land cover change (Nikolaishvili & Dvalashvili, 2015; Sefidi et al., 2022; Sekercioglu et al., 2011). Therefore, simulation experiments designed to forecast the potential distribution of woody species, including the direction of any potential migration, or an “escape route” under different climate change scenarios are timely and pertinent.

Similar studies aimed at forecast have been conducted in the past for Europe (Hickler et al., 2012), or at a coarser scale globally (Sitch et al., 2003; Smith et al., 2001), and for the historical period, using high‐resolution climate data to determine the potential forest cover and productivity, recently for Türkiye (Ekberzade et al., 2022). However, resolving potential centennial forest composition and distribution using high‐resolution climate data at a regional scale including Asia Minor, the Caucasus, and northern part of the Middle East, is yet unprecedented.

In this study, through a series of model experiments, we aim to answer three overarching questions under a worst‐case scenario of atmospheric emissions: (1) how may the potential forest ranges and composition change by the end of the century? (2) Will there be potential regional extinctions among the key forest taxa simulated? (3) Will any site emerge as a potential refugium for certain taxa in a warmer and less predictable world?

## 2 MATERIALS AND METHODS

### 2.1 Dynamic vegetation model

Regional vegetation dynamics for the study area, with a focus on species' stand density and biomass, were simulated for the period of 1961–2100 using the process‐based dynamic regional‐to‐global vegetation model LPJ‐GUESS v.4.1. The model requires climate datasets as input files for temperature, precipitation, solar radiation, wind, and relative humidity. Additionally, atmospheric CO_2_ and a gridded soil dataset are needed for the model to simulate species distribution based on ecological notions such as competition for resources and disturbance (Smith, 2007; Smith & Siltberg, 2021). LPJ‐GUESS simulates growth, establishment, and mortality of a list of species categorized under plant functional types (PFT) for a given stand, providing monthly or annual results. The PFTs are assigned species‐specific bioclimatic thresholds and phenological and ecological properties (those for these sets of simulations are provided in the Appendix 1: Tables A1 and A2). All PFTs defined in the instruction file for a simulation are assigned to each stand with a “grass only” initial condition, and only those with bioclimatic preconditions that match the climatic conditions for the stand and its soil type survive to enter into competition with their cohorts. Individual plant traits mandate the outcome for individual species' survival, while their response to changes in climatic drivers and disturbances determine the final stand structure and composition.

### 2.2 Study area

**FIGURE 1:**
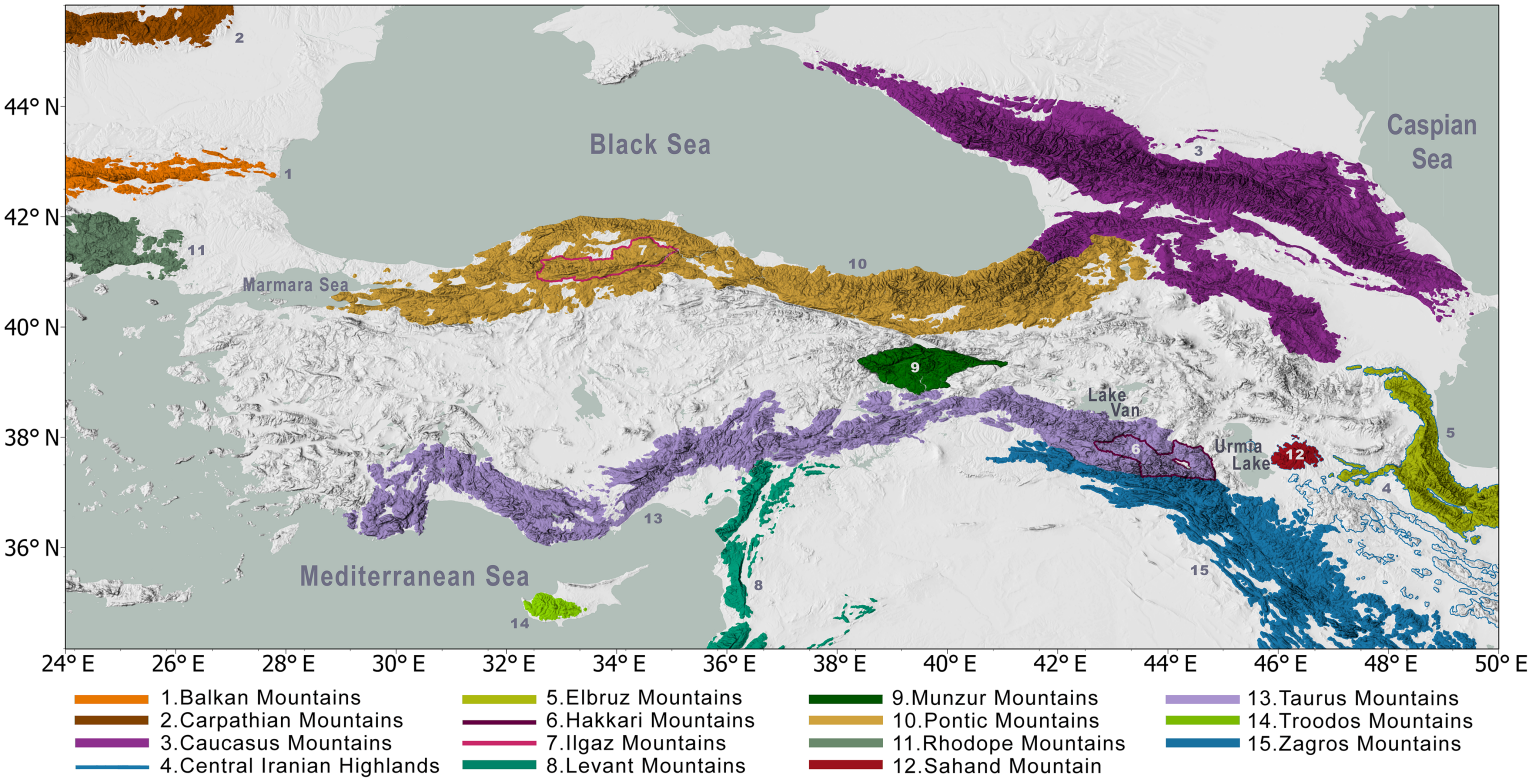
Main mountain ranges and important segments within the study region, adapted from Snethlage et al. (2022).

The study area is a rectangular window encircling the Anatolian Peninsula and its immediate surroundings between longitudes 24–50° E and latitudes 33–46° N. The mountainous terrain of the peninsula rests on a high plateau crisscrossed by several active fault lines underneath, with the mountain ranges that line up its southern and northern coastlines acting as reminders of a long history of tectonism and contributing to the complex topography of the peninsula (Akkemik et al., 2022; Harzhauser et al., 2007; Kuzucuoğlu et al., 2019). The main mountain ranges join neighboring systems such as the Caucasus Mountains in the northeast and the Zagros Mountains in the southeast. For this study, we kept them within our study area to better monitor potential future range shifts of species as these systems contribute to the natural distribution of taxa within the region both as agents of vicariance, and islands of biodiversity (Figure 1).

**FIGURE 2:**
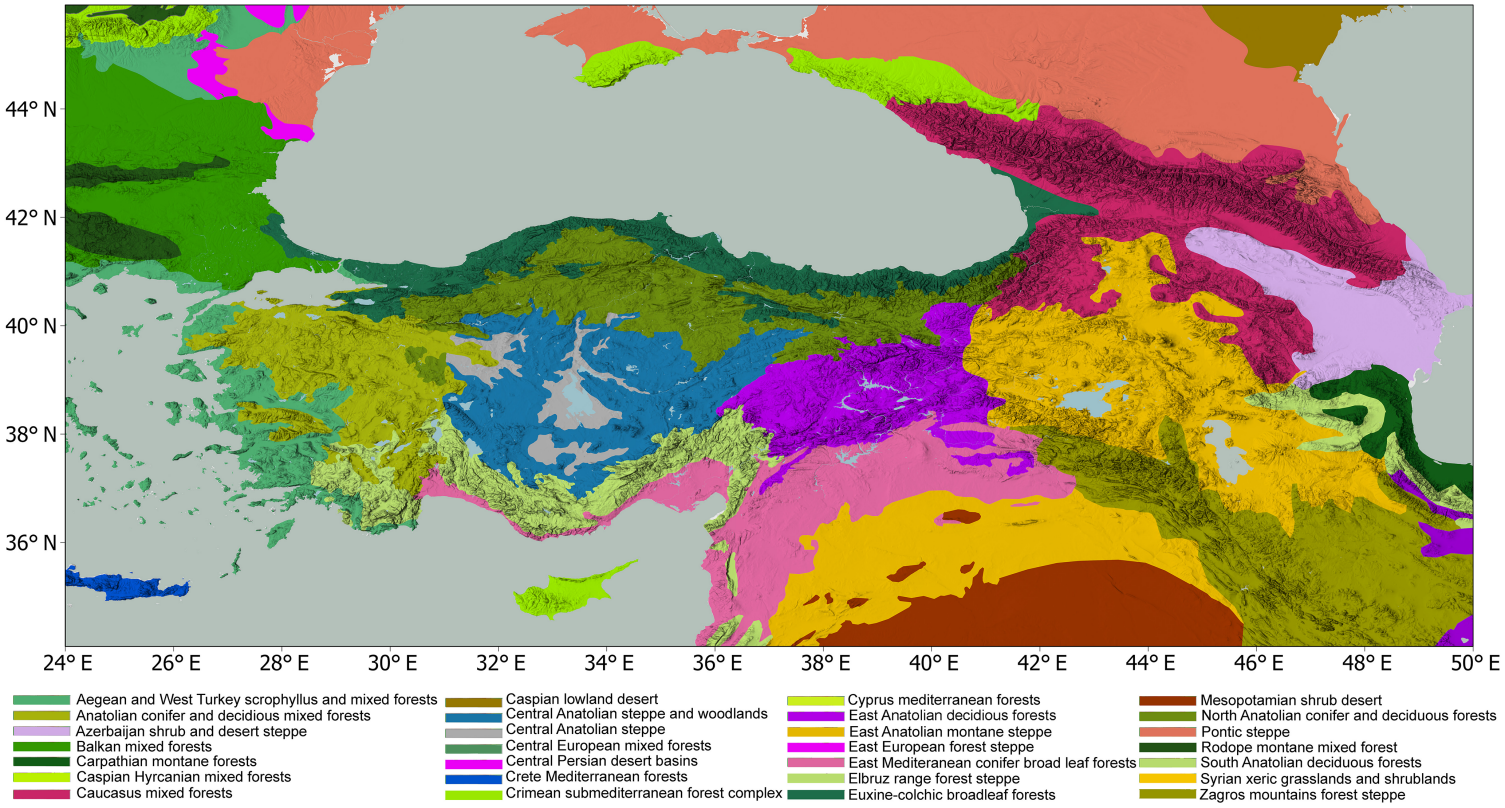
Distribution of the terrestrial ecoregions within the study area adapted from Dinerstein et al. (2017).

While the woody forest taxa, distributed along different climatic belts of these mountain systems show continuity throughout the high terrain, vegetation composition changes due to climate and topography. Figure 2 shows the diverse ecoregions within the study area – providing a higher resolution alternative from a biogeographic perspective than biome distributions – highlighting the close proximity of diverse vegetation combinations and forest, shrub, and grassland distributions (Olson et al., 2001).

### 2.3 Input datasets and modeling protocol

The model was forced with climate datasets of daily temporal resolution, while the spatial resolution of ERA5‐Land reanalysis (9 km horizontal) was preserved. This lent an area of 81 km^2^ to each stand, divided into 1000 m^2^ patches with 25 replicates. We designed our simulations with an average return period of 200 years for the generic patch destroying disturbance, and a model spin‐up period of 600 years. The output files listed year‐end results.

We used annual global mean of atmospheric CO_2_ concentrations from The Global Monitoring Division of NOAA/Earth System Research Laboratory for the period 1961–2022, and global projections modeled with MAGICC7.0 (Meinshausen & Nicholls, 2018) for the period 2023–2100. Our soil dataset was aggregated from the General Directorate of Forestry (GDF) of Türkiye's national dataset (2021), which we initially converted to a raster dataset with 1 km resolution, and then upscaled it to 9 km horizontal resolution, each grid representing the mode of nine grids within its perimeters. For regions outside Türkiye, we interpolated the 0.5° global soil texture dataset compiled by Ben Smith and provided with the model code, to 9 km horizontal resolution and used that with his permission (personal communication).

LPJ‐GUESS v 4.1 offers two fire models to choose from: GLOBFIRM, and BLAZE. For our simulations, the wildfire combustion model BLAZE was selected. BLAZE requires three additional climate datasets, minimum and maximum temperature, and wind speed to calculate fire probability and intensity for each stand (a stand in our simulations corresponds to a grid in the climate datasets). LPJ‐GUESS v 4.1 coupled with BLAZE, also runs an inherent algorithm where the burnt area is calculated by means of a simple statistical model, SIMFIRE. During these calculations each stand is assigned a biome rank, which are then used to calculate the fraction of a grid‐cell which will be consumed by wildfire. The algorithm for SIMFIRE, by design, accounts for human presence. However, since our simulations were aimed at forecasting changes in the potential forest cover and vegetation composition of our study area without any human presence, we altered the code so that no human population density was assigned to any stand simulated.

### 2.4 Parameterizing PFTs

The default PFT settings for European species provided with the model code required calibrations to better reflect their historical distributions within our study area. Following the same methodology of parameterization as in Ekberzade et al. (2022), we further finetuned our list of PFTs, taking into consideration the taxa's sensitivities for additional climatic inputs which we ran the current version of the model with: minimum and maximum temperature, and relative humidity. To do this, we superimposed climate data averaged for 1990–2020 from ERA5‐Land reanalysis, over the taxa's recorded distributions in the GDF's national forest inventory dataset (2021), focusing on specific GIS coordinates acquired in our site visits.

Also, through site visits to locations within the Anatolian Peninsula where forests exhibited little anthropogenic disturbance, bioclimatic limits of two additional taxa, *Alnus glutinosa* and *Quercus macranthera* were identified and included in our simulations. *Corylus avellana*, naturally occurring in shrub form in the study area was changed as such from its default form of tree in the model instruction file, and the bioclimatic properties of the rest of the shrub genera were calibrated to reflect their current distributions. Since species may show regional acclimatization, in our parameterizations we focused on regions where the taxa had natural distributions, and were well established with healthy present‐day populations: that is, for fir, we recalibrated their bioclimatic limits according to their populations in western and central Pontides; for cedar, we chose populations on the eastern segment of the Taurides to better observe their potential continuity along and beyond the Zagros Mountains. A complete list of our final PFT parameterizations are provided in Appendix 1.

We verified our simulation results for the historical period, again using the same methodology as in Ekberzade et al. (2022), where we superimposed the simulated ranges of the taxa over occurrence data. For occurrence data, in addition to the GDF dataset and our field observations, we also referred to a number of sources including the European Forest Genetic Resources Programme's species distribution atlases (EUFORGEN, 2021); literature review; and consultation with forestry and botany experts.

### 2.5 Climate datasets for future projection

For the historical period, ERA5‐Land hourly data were calculated as daily sum for precipitation and as daily mean for all other datasets. For the scenario period, five different GCM datasets, contributions to the sixth phase of Coupled Model Intercomparison Project (CMIP6) experiments, were selected based on their spatio‐temporal resolution (daily data with centennial timeseries; spatial resolution 50–100 km) and our emissions scenario of choice (Shared Socioeconomic Pathways 5–8.5). Data were interpolated to 9 km horizontal resolution, and bias corrected with the climatology of the reference period (1995–2014) from the ERA5‐Land dataset. The reference period was selected in accordance with the 2021 report from IPCC (2021). Table S1 in Appendix S1 lists the GCM datasets used in our simulations.

### 2.6 Processing model output

For all dataset calculations and mapping, Climate Data Operators (Schulzweida, 2019), R programming language (R Core Team, 2021), and ArcGIS Pro v.3.0 were used. Annual results of the different simulations from individual GCM runs, and four sets of 30‐year means from the beginning (1961–1990), middle (1991–2020 and 2031–2060), and end of the timeseries (2071–2100) were calculated and plotted using R, to observe patterns of convergence and divergence among different model datasets and simulation results. Sum of absolute deviations was the chosen method in this initial analysis to understand where and when the simulation results for biomass, density, as well as the input datasets of precipitation and temperature diverged (Appendix S1, Figures S2–S5).

The ensemble medians of the period mean of output files were aggregated to large datasets in R for the beginning and the end of the simulations, and transferred to ArcGIS Pro for detailed bivariate analysis. We ran our analyses on the median of 30‐year means for the beginning and end of the individual GCM simulations (1961–1990 and 2071–2100). To further analyze the spatio‐temporal changes in our simulation results, we added Shuttle Radar Topography Mission's (SRTM) 30 arc‐second global digital elevation dataset resized to 9 km resolution.

For comprehensive analyses of the simulated responses of individual taxa, scales of our taxon‐specific bivariate maps (shown in Appendix 2) were adjusted for density across taxa, and for biomass, scales were adjusted for minimum and maximum values within hardwood and softwood groups. This rescaling allowed us not only to compare changes in biomass and density per taxon overtime, but also differences between species in terms of range preference, response to changes in climate and signs of potential migration. For density, we selected a minimum number of 100 individuals/ha of a given taxon as our threshold; for biomass we kept our minimum threshold at 1 kgC/m^2^ for softwood and 2 kgC/m^2^ for hardwood species (1 kgC/m^2^ was preserved in maps where multiple taxa were plotted). Values below these thresholds were classified as both low density and low biomass for a given stand for the taxa in question, and when they occurred together, no color was assigned to those grids. We selected our minimum simulated biomass thresholds in our analyses with a general consideration for the global values reported by Erb et al. (2017). For our minimum density thresholds, we picked a high enough number that would allow us to concentrate only in places where the taxon being analyzed established populations of higher number of individuals (including seedlings) than our minimum density threshold.

## 3 RESULTS AND DISCUSSION

### 3.1 Latitude or altitude?

**FIGURE 3:**
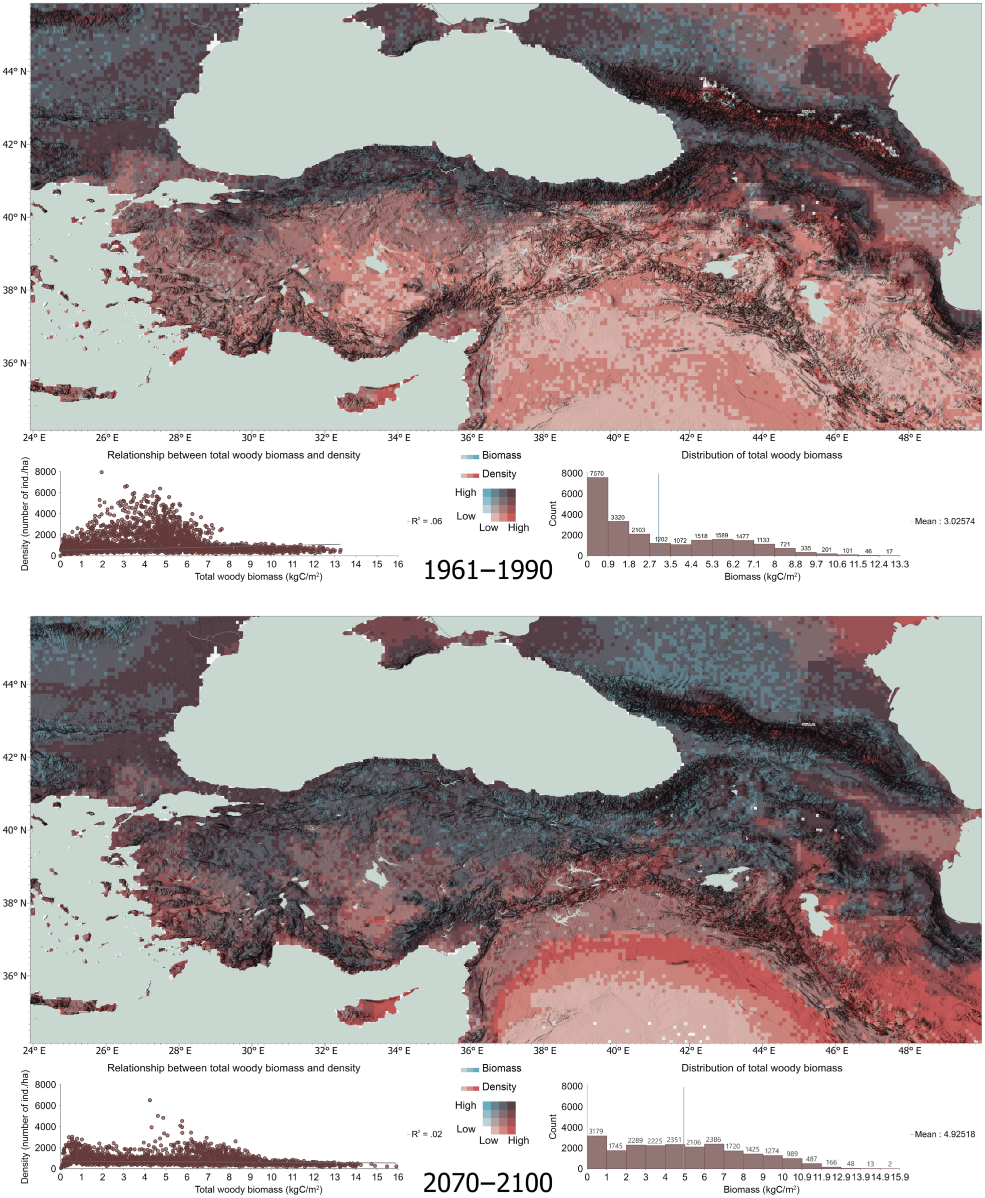
Total woody biomass and density for the beginning and end of the simulation period. Scales of maps have been standardized for comparison.

Figure 3 shows the results of our bivariate analyses of total density and total woody biomass from our simulations for the beginning and end of our simulation period. The simulated total density of woody taxa shows an overall centennial decrease per stand from an average maximum of 6000 individuals/stand in parts to below 4000, which is compensated by an increase in biomass, a change that reflects the natural correlation between biomass and density: as woody taxa grow and gain biomass both above and below ground overtime, competition for space becomes a primary limitation to seedlings' success.

The simulated increase in woody biomass is largely consistent across our study area and more pronounced for the central Anatolian steppe ecosystems, and the northern segment of our study area (latitudes 36° N and above). North‐central and western parts of Anatolia (especially southeast of the Marmara Sea) and the eastern highlands show a simulated centennial increase in total woody biomass, as well as the Taurus Mountains along the Mediterranean coast. Simulated changes in density in the Mesopotamian shrub and desert ecoregions by the end of the century duly hint at the distinct ecoregion “belts” for this area. In the Zagros Mountains Forest Steppe ecoregion, with maximum altitudes between 2000 and 3000 m for the study area, a potential increase in density of woody taxa with a substantial increase in biomass in higher terrain is simulated for 2100.

Our simulations show a centennial increase in total density and biomass of woody taxa for the southern Black Sea region, especially along the eastern segment of the Pontic Mountains (the mountain system is also referred to as North Anatolian Mountains), while the majority of the species in the northern part of the study area appear to gain in both density and biomass. In the Caucasus Mountains, especially in higher elevations, in the Pontic Steppes north of the Crimean Peninsula, and in the East European forest steppes along the western Black Sea, higher potential biomass and density can be seen by the end of the century. The Rhodope and Balkan Mountains, and the eastern edge of the Carpathians also show high simulated biomass.

**FIGURE 4:**
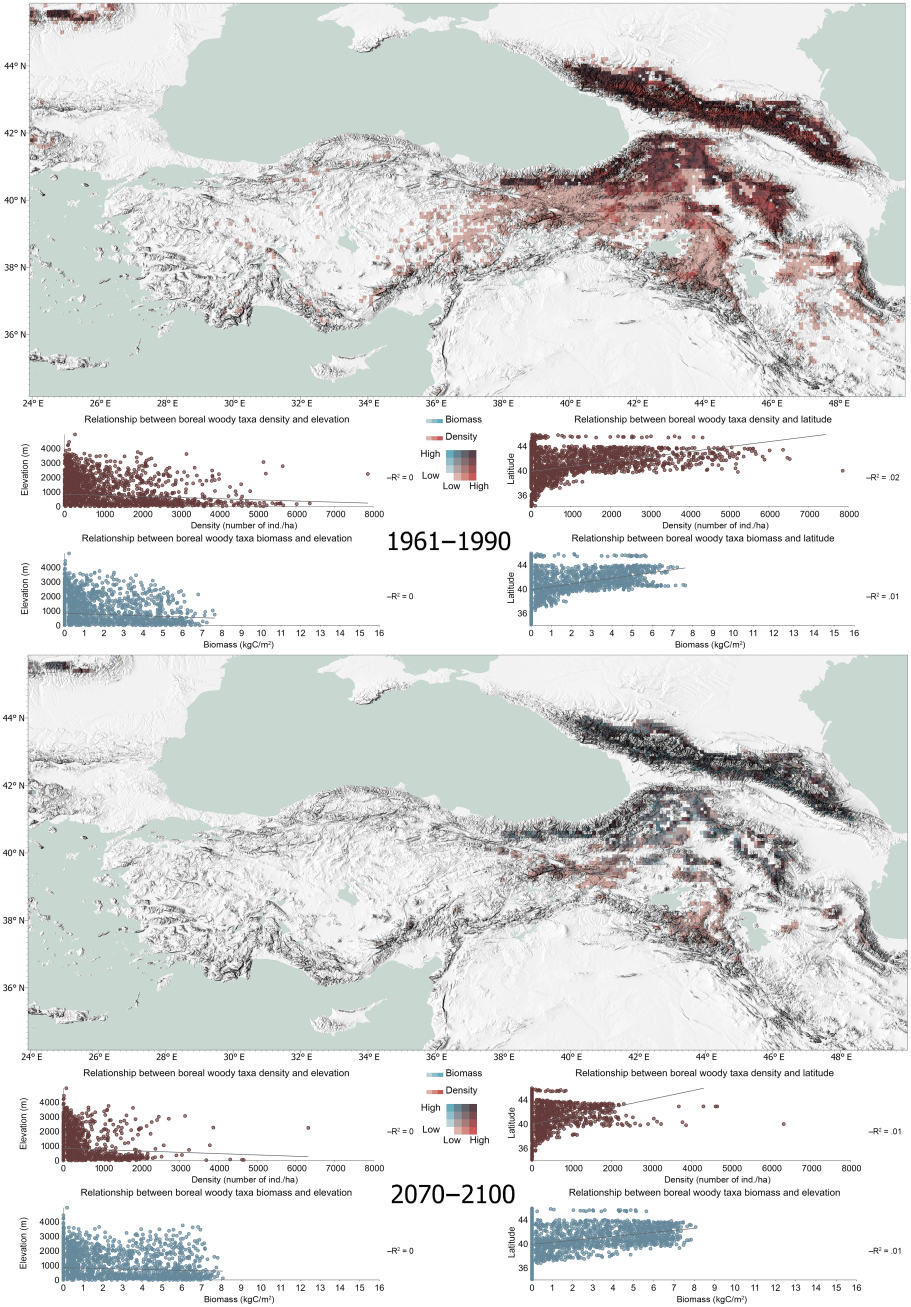
Density and biomass relationship for the boreal taxa for the beginning and end of the simulation.

When we further analyze simulated biomass and density for the two distinct bioclimatic groups, a clear pattern emerges. Figure 4 highlights the potential shift from high density and high biomass to low density and high biomass of the boreal taxa, where they consolidate their ranges on mountain regions, increase their biomass on higher altitudes with negligible signs of a latitudinal movement by 2100. The maps suggest a climbing tendency of the boreal species – with mostly *Pinus sylvestris* taking the lead – in eastern Anatolia, potentially converging around highland regions of Munzur Mountains, with elevations over 3300 m (and a natural distribution range for *P. sylvestris*). Also, albeit with lower biomass, they consolidate their southeastern range around Hakkari Mountains southeast of Lake Van.

**FIGURE 5:**
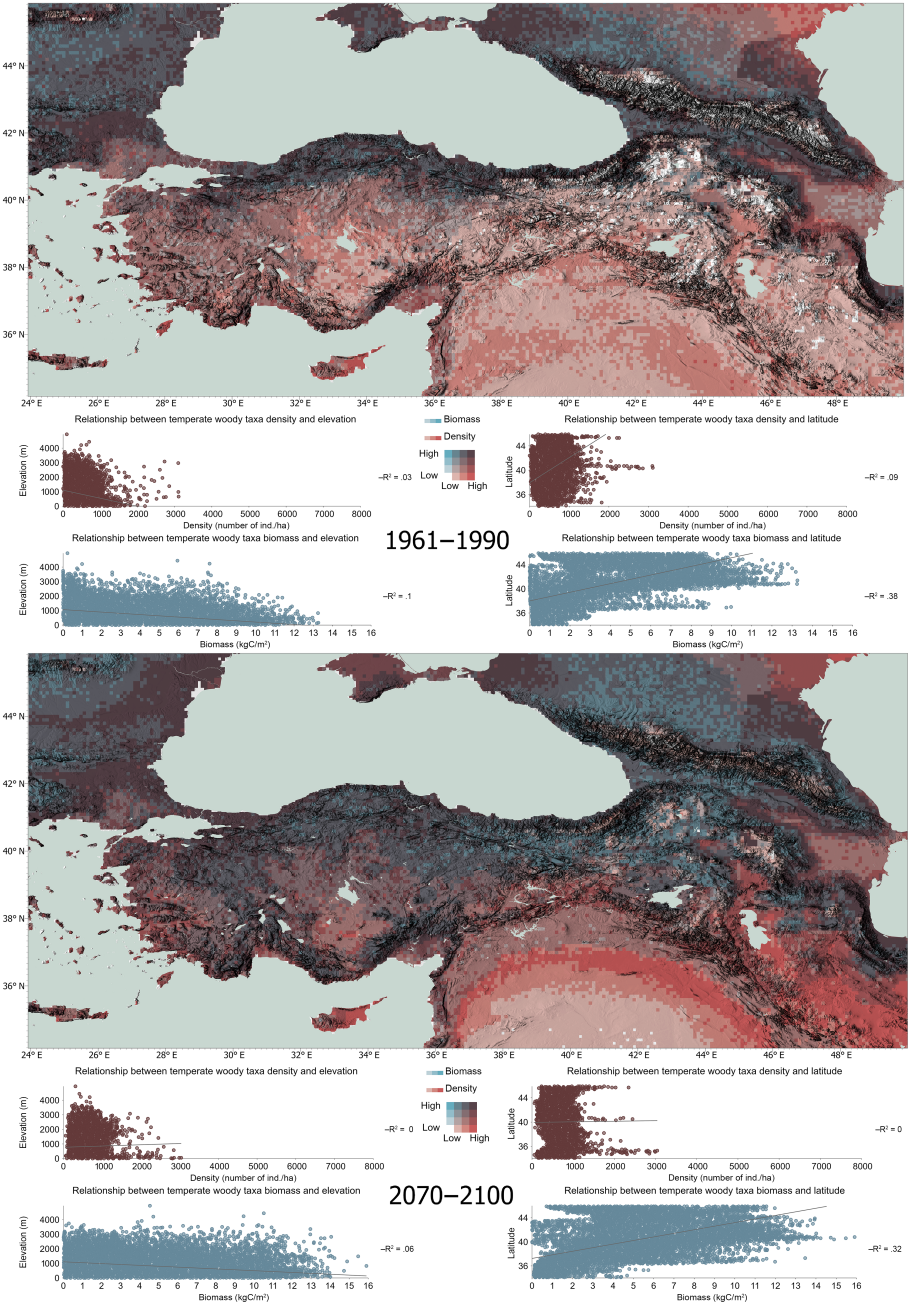
Density and biomass relationship for the temperate taxa for the beginning and end of the simulation.

Figure 5 shows the simulated takeover of our study area by temperate woody taxa by 2100. Drought‐resistant temperate taxa step into dominance, potentially forcing the boreal taxa to a pull‐back under a warming trend. The former not only increase their density, but their biomass as well, a pattern that largely explains the simulated centennial increase in total woody biomass and density in Central Anatolia. Their range expansion does not indicate any particular prioritization for altitude or latitude.

**FIGURE 6:**
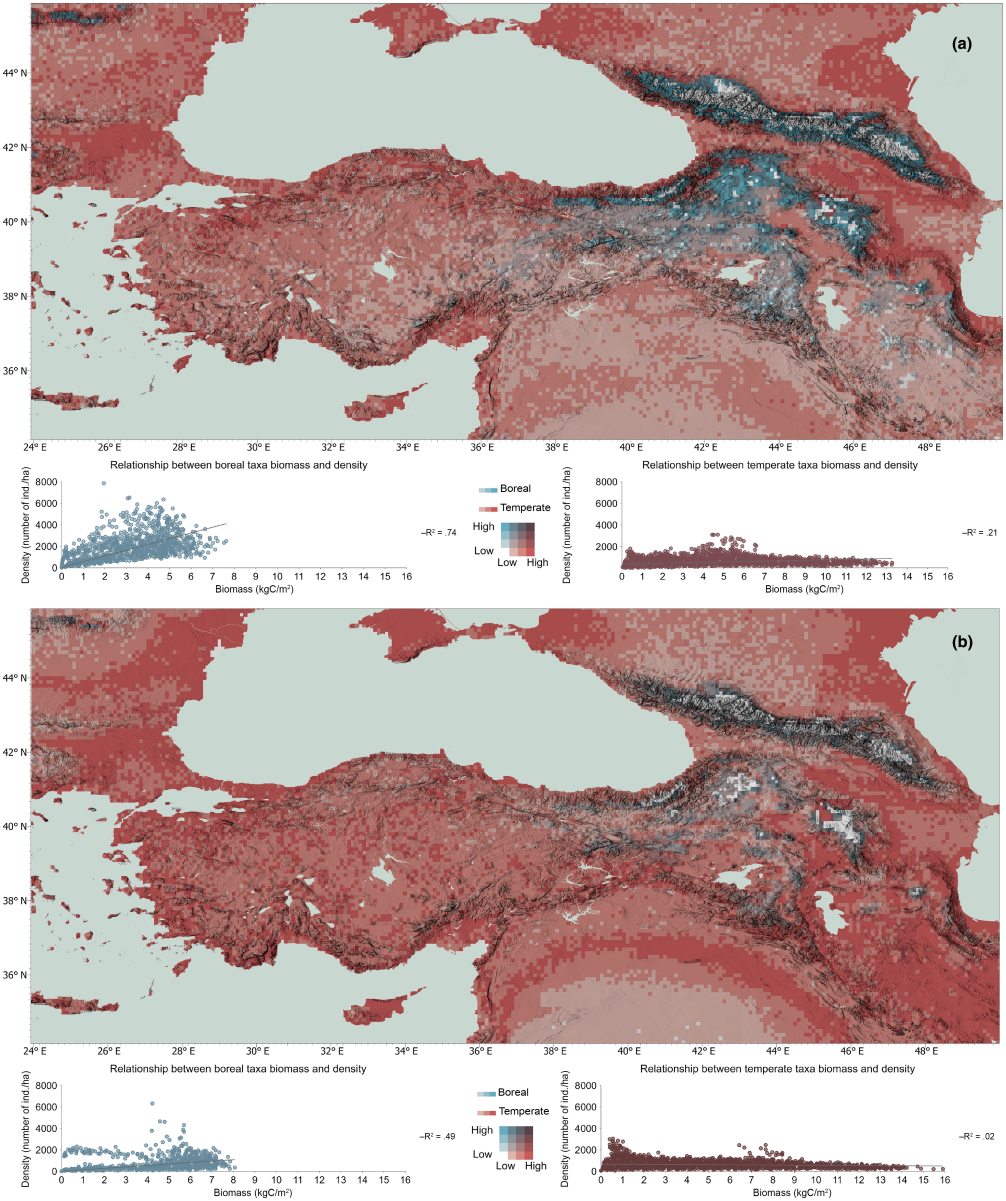
Density for temperate and boreal woody taxa (a) for the beginning (1961–1990) and (b) end (2071–2100) of our simulations. Scatter plots show biomass and density relationship within each group.

The potential takeover of temperate taxa with the retreat in boreal taxa in our simulation results become more apparent when we cross‐compare the relationship of boreal and temperate taxa density: the maps in Figure 6 show boreal taxa pulling back and consolidating their ranges around mountainous terrain, and the scatter plots highlight the noticeable centennial decrease in the boreal species' simulated density.

### 3.2 Climate mandates limitation

**FIGURE 7:**
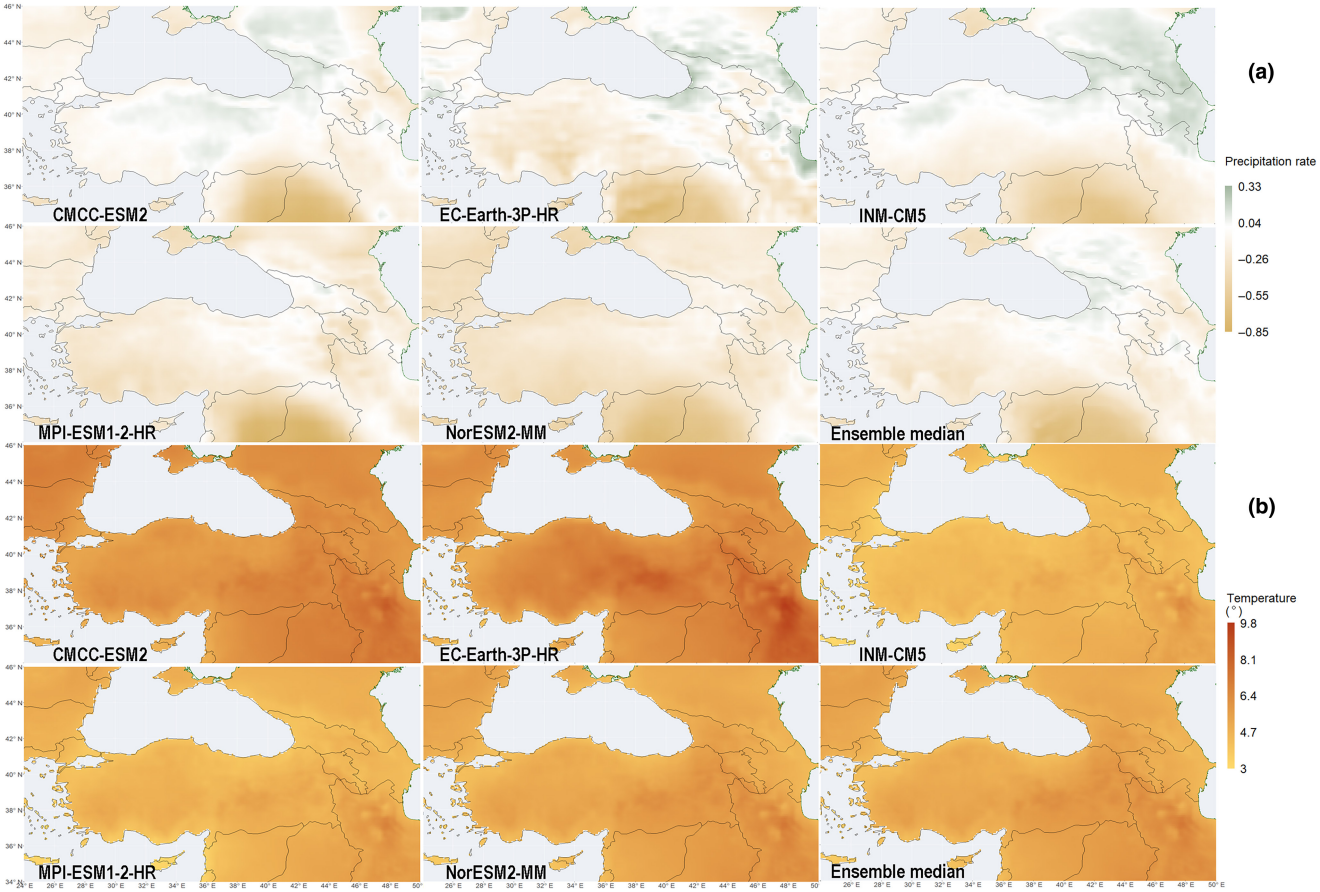
(a) Change in precipitation rate between 1961–1990 and 2071–2100 period means for the GCM datasets and their ensemble median; (b) same for temperature.

While the patterns in biomass and density of woody taxa are pronounced between the beginning and end of the 140‐year simulation period, there is indeed a gradual and steady shift in potential forest composition overtime, favoring drought‐tolerant taxa with a survival capacity under a wider range of temperatures. This can partially be explained by the consistent drying pattern for the southeastern segment of our study area in the five GCM datasets with which we forced the model with, and their ensemble median (Figure 7). Northwestern and western Anatolia, and western and central segments of the Pontic Mountains show a decrease in precipitation (around 30%) in the ensemble median, with only the eastern segment of the Pontic Mountains – where there is now a temperate rainforest – and the southeastern Black Sea coast showing an increase in precipitation (up to 33%). The datasets also forecast consistent warming for our study area, where the centennial increase in temperatures is roughly within the range of 4.5 and 6.5°C in the ensemble median.

**FIGURE 8:**
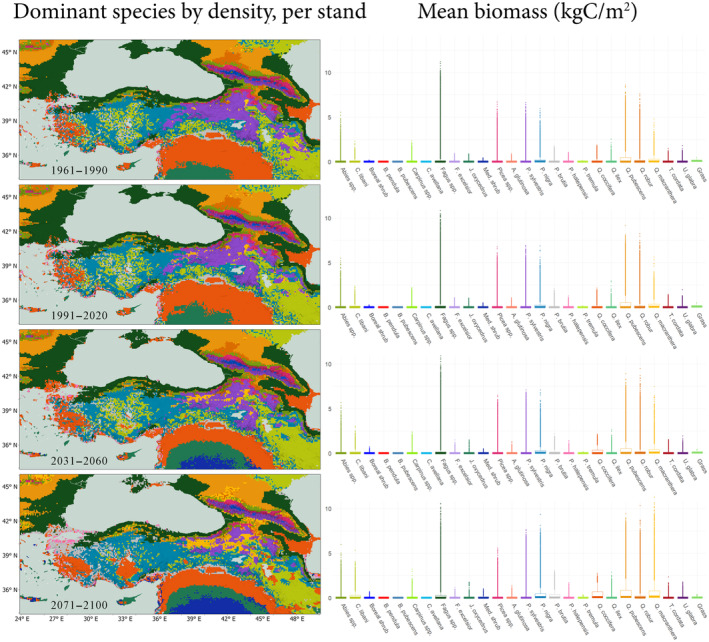
Dominant species by density and species' biomass for the median of the historical, and three scenario period means. Boxplot colors correspond to the taxa in the map. Grass subgroup represents C3 grasses.

Figure 8 shows the simulated spatial distribution of density dominance of taxa per stand and their total biomass calculated as medians of individual GCM results from the means of the historical period, and three time periods from the beginning, middle and end of the simulation period (individual GCM results are in Figure S1, Appendix S1). The potential increase in biomass of the four deciduous Quercus species, especially for the two taxa – *Q. pubescens* and *Q. macranthera*, both with higher drought tolerance – can be seen in the boxplots. Where the Quercus taxa's gain in both total biomass and spatial dominance is significant within the study area, *Fagus* spp. (representing joint ranges of *F. sylvatica* and *F. orientalis*) seem to preserve their biomass in their strongholds in the Pontic Mountains, and in the eastern segment of the Taurus Mountains, occasionally at a relative cost to their dominance. This future pattern of *Fagus* spp. potentially pulling back from their dominant positions is most pronounced in the central part of the Crimean Peninsula, and around the Marmara Sea, where the taxa lose their density dominance in the mixed forest ecosystems.

A similar pattern emerges for *P. sylvestris* as well, where the taxon potentially loses its density dominance considerably in the Eastern Highlands, replaced by cold and drought‐tolerant *Q. macranthera* in the north and eastern Anatolia; and by the continental temperate conifer *Pinus nigra* in the south, on the eastern segment of the Taurus Mountains and along the Zagros range.

### 3.3 Taxon‐specific survival strategy

#### 3.3.1 Cedrus libani

*Cedrus libani* can be seen potentially taking over parts of the southeastern edge of *P. sylvestris*'s density dominance (between lakes Van and Urmia), keeping its density dominance in the Central Iranian Highlands by the end of the century. However, for the most part of its simulated distribution range, *C. libani* also gradually loses to competition from *P. nigra* and the Mediterranean shrub taxon *Q. coccifera*.

In addition to projected changes in precipitation patterns and the forecasted increase in temperatures, the range shifts of taxa can also be explained by the projected change in growing degree days (GDD) between the beginning and the end of our simulations (Figures S6 and S7, in Appendix S1 show GDD 5°C for the ensemble median and individual GCM projections, respectively). *C. libani*, in its native Taurus range, starts off in mixed conifer forests alongside *P. nigra* and continues to climb, and with successful hold in karstic terrain with negligible amounts of topsoil, establishes healthy and pure cedar forests in high altitudes. Despite the taxon's general preference for higher elevations than *P. nigra*, and tolerance for cooler temperatures, it requires a higher GDD5 minimum for establishment than *P. nigra*. On the other hand, *P. nigra* can tolerate higher temperatures, but prefers a lower GDD5 for establishment, and cannot tolerate drought as well as *C. libani* (Köse et al., 2012). Thus, when both taxa compete, the latter comes out as the winner in Central Iranian Highlands in our simulations: despite an increase in projected temperatures in this region, the GDD5 stays within *C. libani*'s establishment range, and the taxon can potentially withstand the projected drying in this area, where *P. nigra* cannot (Doğan & Köse, 2019). Table 1 details the key taxa's simulated range expansion/contraction and the potential change in their stand dominance.

**TABLE 1:** Range expansion or contraction and gain or loss in areal dominance for key forest taxa simulated.

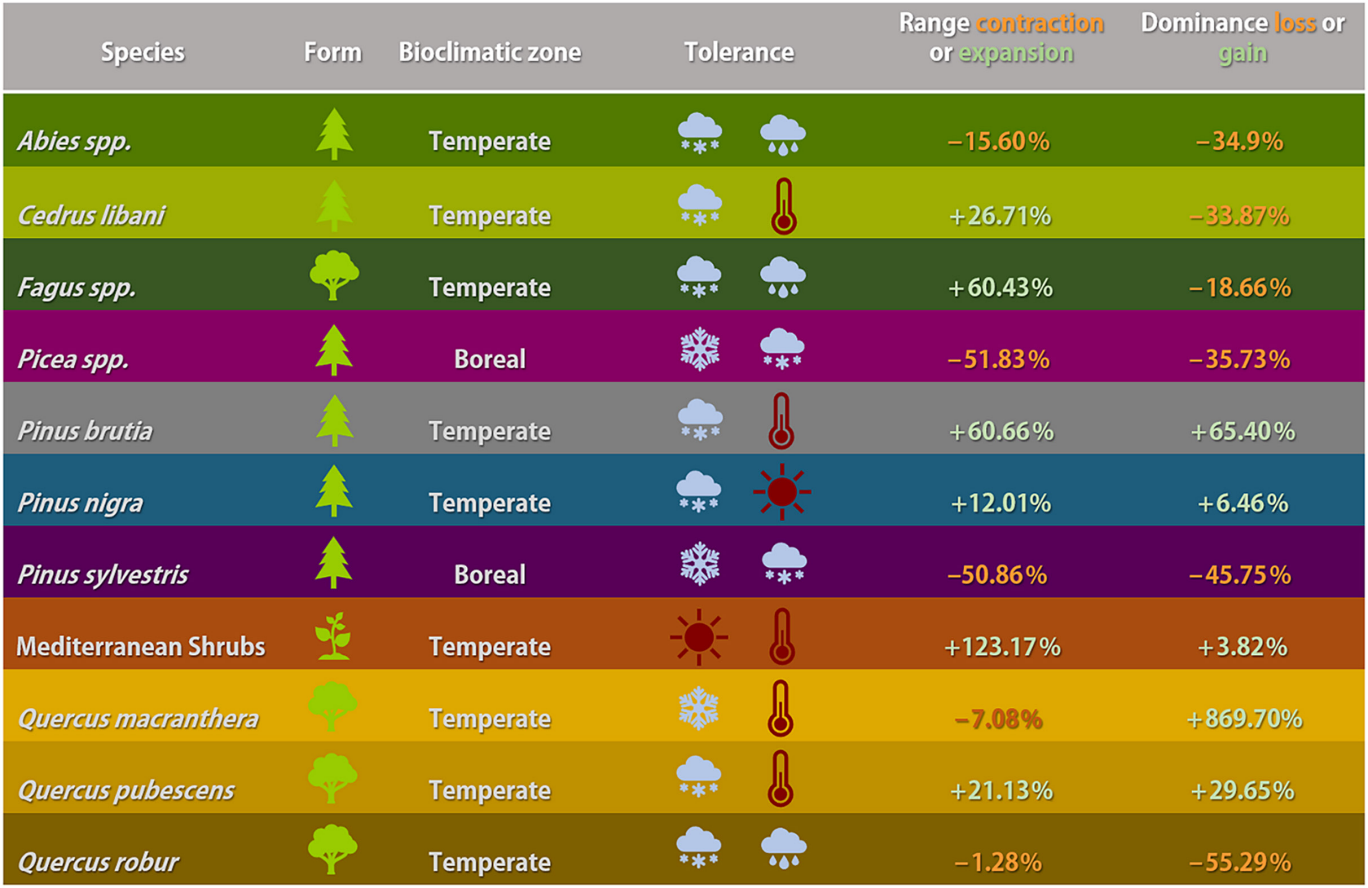

*Note*: Mediterranean shrubs represent simulated joint ranges for Mediterranean raingreen shrubs, *Juniperus oxycedrus* and *Quercus coccifera*. 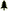: needleleaf; 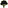: broadleaf; 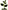: shrubs; 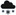: mostly snow, some rain; 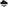: wet/cool; 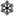: snow and ice; 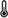: hot/dry; 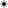: warm/dry. Line colors correspond to the color of the taxa in Figure 8.

#### 3.3.2 *Fagus* spp.

**FIGURE A1:**
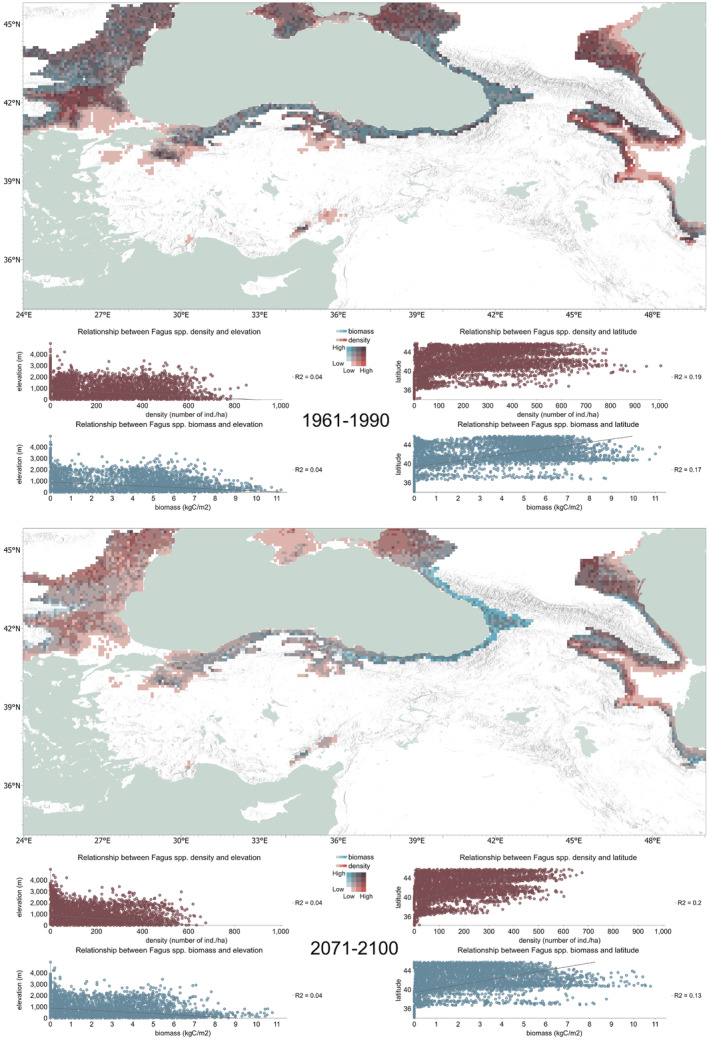
Density and biomass relationship for *Fagus* spp. for the beginning and end of our simulations. Scatter plots show relationships of density and biomass to elevation and latitude.

The species‐specific bivariate maps showing density‐biomass relationships in Appendix 2 highlight the nature of the simulated pull‐back pattern for *P. sylvestris* and *Fagus* spp., and the oaks' potential range expansion. *Fagus* spp. lose their density while somewhat retaining their biomass within their potential distribution range towards the end of the century, culminating in a transfer of their populations' density dominance in their areas of retreat to more drought resilient taxa (Figure A1, Appendix 2 and Table 1). This simulated pattern of a future handover of density dominance by *Fagus* spp. can partially be explained by an optimization strategy: the taxa preserve their potential centennial dominance along their southern Black Sea distribution around the Pontides where they face lesser resource limitation (Figure 7a), but lose both their density and biomass on the continental edges of this mountain system, where they give up dominance but continue to exist in smaller numbers within mixed forest compositions within a wider distribution range (Table 1).

#### 3.3.3 *Abies* spp.

**FIGURE A2:**
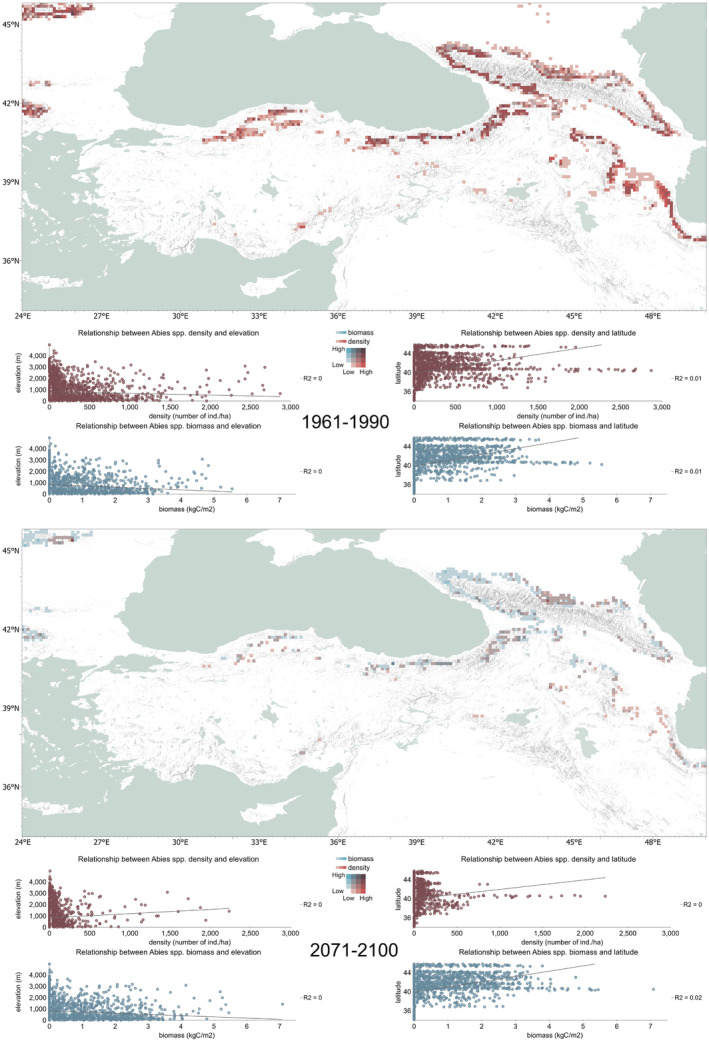
Density and biomass relationship for *Abies* spp. for the beginning and end of our simulations. Scatter plots show relationships of density and biomass to elevation and latitude.

When we consider the centennial density‐biomass relationship of *Abies* spp. (representing joint ranges for *A. nordmanniana* and *A. bornmülleriana* in the southern Black Sea coast, *A. borisiiregis* in the eastern segment of the Balkan Mountains, and partially *A. cilicica* in its centro‐eastern Taurus range), a potential shift from high to low density is seen: the taxa preserving and increasing their biomass in areas where they preserve their foothold (Figure A2, Appendix 2). A noticeable range contraction is simulated with the taxa pulling back from lowlands and investing in their biomass in the highlands. The observed preference of *Abies* spp. in the study area for an altitudinal niche of above‐the‐deciduous‐below‐the‐boreal‐belt is also supported by our simulations: despite their limited spatial resolution, our results highlight the clustering of the taxa's distribution along mountain ranges, suggesting an initial prioritization of an altitudinal shift instead of a latitudinal one when confronted with a warmer and drier climate. Considering the substantial loss of their simulated density dominance (Table 1), the thick, pure fir forests we are accustomed to seeing in the western and central part of the Pontic Mountains may become more heterogeneous by the end of the century, as the taxa may be forced to give up their stand dominance and become a “member of the team”.

#### 3.3.4 *Picea* spp., *Pinus sylvestris* and a potential refugium for boreal taxa

**FIGURE A3:**
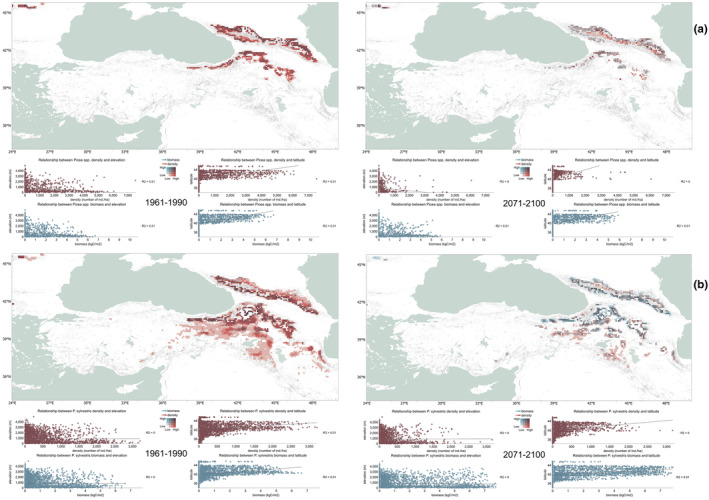
Density and biomass relationship for *Picea* spp. (a) and *Picea sylvestris* (b) for the beginning and end of our simulations.

The boreal conifer taxa *Picea* spp. (representing joint ranges of *P. orientalis* and *P. alba*) with their dominant populations converging around large mountain systems, also contract their simulated ranges overtime, while preserving their biomass in places where they can keep a foothold: in the Caucasus Mountains and the eastern segment of the Pontic Mountains (Figure A3a, Appendix 2). *P. sylvestris*, much like *Picea* spp. seems to follow a similar strategy, its populations increasing their biomass within their bioclimatic niches in the Caucasus range; keeping a foothold in both the Munzur and Hakkari Mountains (with summits of 3462 and 4116 m, respectively), while potentially contracting its distribution range by 2100 (Figure A3b, Appendix 2). And despite *C. libani* becoming dominant around *P. sylvestris'* southeastern hold in our simulations, the latter consolidates its range around Hakkari Mountains on the eastern segment of the Taurides. Our simulation results for *Fagus* spp., *Picea* spp., *P. sylvestris*, and in part *Abies* spp. are in line with the results from species distribution models reported by Dyderski et al. (2018), for Europe.

#### 3.3.5 Limitation mandates competition, competition mandates migration

One thing that the temperate evergreen needleleaf *Abies* spp., temperate deciduous broadleaf *Fagus* spp., boreal evergreen needleleaf *Picea* spp., and boreal evergreen needleleaf *P. sylvestris* have in common is their sensitivity to drought. While these taxa prefer cooler temperatures, studies show that when there is water limitation, their populations suffer under drought stress (Arend et al., 2021; Camarero et al., 2015; Obladen et al., 2021). Also, spruce forests may become more susceptible to insect outbreaks (Boczoń et al., 2018; Hlásny & Turcani, 2013), and beech populations may record diebacks (Arend et al., 2022; Schuldt et al., 2020). *Abies* spp., although more resilient, also have a hard time surviving prolonged dry spells and are sensitive to potential changes in seasonality, relinquishing their ranges to more drought‐resistant taxa when they can no longer compete for resources (George et al., 2015; Mátyás et al., 2021).

**FIGURE A4:**
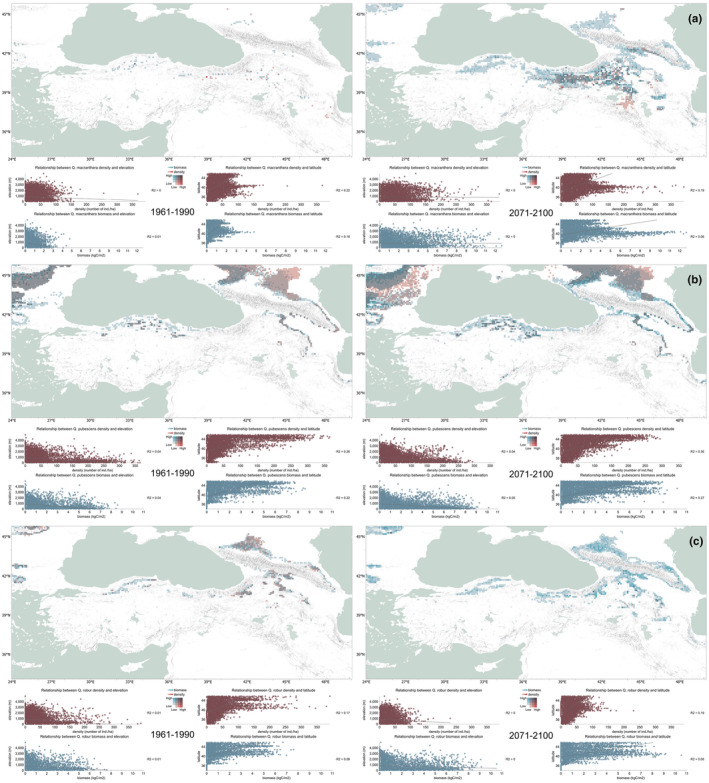
Density and biomass relationship for *Quercus macranthera* (a), *Quercus pubescens* (b), and *Quercus robur* (c) for the beginning and end of our simulations.

Additionally, while the displacement of *P. sylvestris* from the majority of the Eastern Anatolian Highlands by 2100 can be explained by potential resource limitation, the role of competition in the boreal taxon's simulated pull‐back should not be underestimated. In our simulation results, areas where *P. sylvestris* largely exits from north of the Eastern Highlands – where GCMs used in our simulations project a warming pattern – are in turn populated mainly by *Q. macranthera*, a native drought‐resistant broadleaf with high tolerance for continental climate; and areas where the taxon pulls back from density dominance (along with *Abies* spp. and *Picea* spp.) in central and western Pontides, are populated with three summergreen oak species simulated, whose biomass and density relationships are shown in Figure A4, Appendix 2.

#### 3.3.6 *Quercus macranthera*, *Q. pubescens*, and *Q. robur*

While our simulation results for *Q. macranthera*, *Q. pubescens*, and *Q. robur* show an overall increase in biomass throughout our simulation period, at the taxon level of our analyses, individual preferences over resources that finetune their distribution ranges become apparent. *Q. macranthera*, with considerable increase in biomass and density, preserves its areal presence while substantially exerting its dominance; as *Q. robur*, its sister taxon, records a loss in stand dominance; and *Q. pubescens* expands its distribution range in the steppe ecosystems in the north. And although all three taxa have deep root systems capable of tapping into underground reservoirs at times of drought, in comparative studies, potential provenance‐specific adaptation notwithstanding (Arend et al., 2011), *Q. robur* has been identified as the least drought resistant of the three, with *Q. macranthera* and *Q. pubescens* showing similar sensitivities (Genard‐Zielinski et al., 2018; Opala‐Owczarek et al., 2021). However, *Q. pubescens*, a renowned climate‐change‐winner (Früchtenicht et al., 2018) and a generalist (Kirsch, 2021), has tolerance for warmer temperatures than the cold favoring *Q. macranthera*, which is capable of penetrating lower parts of the alpine belt. Thus, with the persistent warming trend across the board in our climate datasets, *Q. pubescens*' range expansion does not come as a surprise. Nevertheless, both oak species' survival success does come at a cost for the four afore mentioned cold‐adapted, drought‐sensitive taxa forcing them to either consolidate their ranges as in the case of *Abies* spp., *Picea* spp., and *P. sylvestris*, or lose their dominance, like *Fagus* spp.

#### 3.3.7 Pinus nigra

**FIGURE A5:**
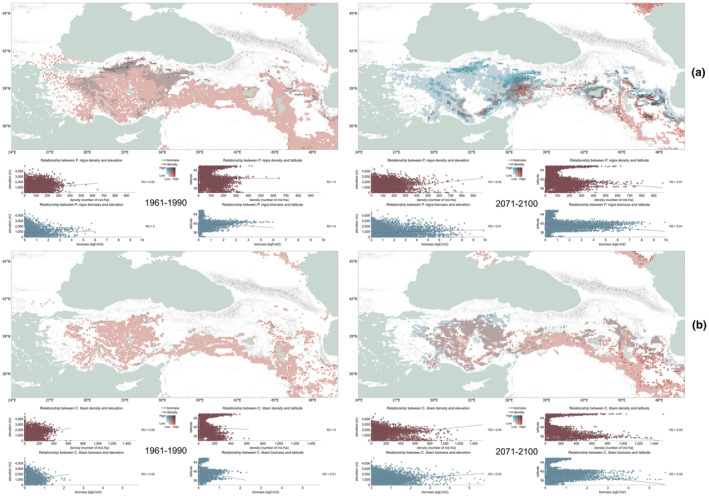
Density and biomass relationship for *Pinus nigra* (a) and *Cedrus libani* (b) for the beginning and end of our simulations.

*Pinus nigra*, a drought‐resistant temperate conifer with an affinity for the continental climate (Candel‐Pérez et al., 2022) takes dominance in Central Anatolia and on the edges of the Central Iranian Highlands in our simulations (prioritizing mid‐latitudes) where our datasets forecast a warming and drying trend for the end of the century. *P. nigra*'s survival success in plantations in northern Iran where it is not a native species has been documented in separate studies (Fataei, 2016; Picchio et al., 2022; Tavankar et al., 2018). The taxon is native to Central Anatolia, and in our simulations not only consolidates its spatial density dominance in this region, but also increases its biomass. Figure A5 in Appendix 2 shows the simulated density‐biomass relationship for *P. nigra* and *C. libani*, where, in the southeast, both taxa potentially consolidate their ranges mainly on the mountain systems by means of high density in the Zagros Mountains and higher biomass in the continental side of the Elbruz Mountains.

#### 3.3.8 *Pinus brutia* and a potential northern refugium

Another key player for forest ecosystems in the Eastern Mediterranean Basin, the drought‐resistant conifer *P. brutia*, potentially expands its range mainly in western‐central Anatolia, Thrace, and the Crimean Peninsula. In the northwestern part of its expansion zone in our simulations, its establishment pattern pushes *Fagus* spp. off its density dominance, since the deciduous taxa cannot withstand competition from the drought‐resistant conifer under a warming and drying climate. However, how much *Fagus* spp. relinquishes its established sites with respect to altitude, and whether this is indeed a shift from the well‐defined broadleaf‐to‐needleleaf vegetation belt transitions in this heterogeneous topography, to more of a mixed‐forest cover throughout, is something we plan to consider in our ongoing research using higher resolution climate data.

**FIGURE A6:**
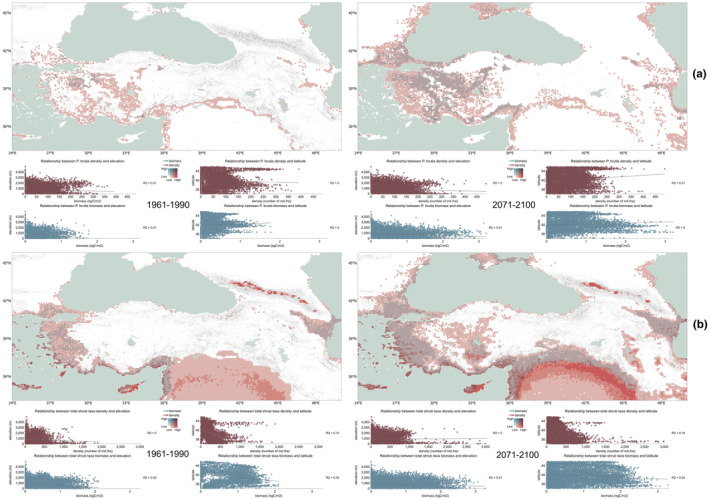
Density and biomass relationship for *Pinus brutia* (a) and all shrub taxa (b) for the beginning and end of the simulation run.

*Pinus brutia*'s simulated success in the Crimean sub‐Mediterranean forest complex – and its potential encroachment towards the Pontic steppe ecosystem in the north (Figure A6a, Appendix 2) – under the right climatic conditions is also supported by palynological and botanical studies, hypothesizing the historical presence and distribution of this taxon in the peninsula and its genetic relationship to varieties in the Anatolian Peninsula and along the western Caucasus coast (Cordova, 2007), and highlights the critical northern refugia potential of this peninsula, specifically for Mediterranean and sub‐Mediterranean taxa.

#### 3.3.9 *Quercus coccifera*, *Juniperus oxycedrus*, and woody species' encroachment

Shrubs also record a centennial increase in their potential range and biomass in our simulations, with *Quercus coccifera* and *Juniperus oxycedrus* taking the lead (Figure A6b, Appendix 2). In Cyprus, an island under the influence of Mediterranean‐type climate, the simulated increase in centennial density with no significant change in biomass for the coastal flats north of the island, signals a potential handover of dominance to *J. oxycedrus* by *Q. coccifera*. The conifer forests on the Troodos Mountains also potentially turn into *Q. coccifera* dominated shrubland (Figure 8 and Figure A6b, Appendix 2), a probable result of the drying pattern in our datasets.

Another result of our simulations is the potential woody species encroachment in grassland areas, such as Central Anatolian and Pontic steppe ecoregions. This simulated takeover by woody taxa can in part be explained by the projected increase in CO_2_ by the end of the century, while the history of anthropogenic deforestation (Aytuğ & Görecelioğlu, 1996) resulting in the current steppe formations in the Anatolian Peninsula should not be ruled out. Sensitivity tests made with a previous version of our model are in support of the hypothesis that elevated CO_2_ levels may exacerbate future woody species encroachment into steppe ecosystems (Verbruggen et al., 2023).

## 4 CONCLUSION

All taxa survived our 140‐years simulation period at some capacity, with no regional extinctions simulated within our study area. Yet, a distinct niche preference between temperate and boreal taxa emerged. The projected minimum 3°C increase in mean annual temperature by 2100, and the decrease in total annual precipitation in our datasets offer plausible explanations as to the drivers of competition between taxa for resources, potential range shifts, and niche partitioning. Our bivariate analyses of simulated biomass and density rendered an initial prioritization of an altitudinal shift rather than a latitudinal one for cold‐favoring taxa, while highlighting the potential competitive advantage of the temperate taxa in a warmer and drier world. The simulated pattern of boreal taxa gathering around mountain‐islands, rather than migrating northerly is an important result, stressing the vital role of the mountain ranges as potential refugia for cold‐favoring species for this century. Our simulations specifically highlight the Hakkari Mountains' potential as a refugium for boreal species for the region south of the 38th parallel by 2100. Similarly, the northeastern segment of the Pontic Mountains and the Caucasus Mountain Range may potentially gain in importance for the cold‐favoring taxa under the current warming pattern.

Several paleo studies including Denk et al. (2019) suggest the presence of a laurel forest ecosystem for the Anatolian Peninsula during the Miocene, based on macro fossil analyses. Absence of forest cover in eastern highlands in Türkiye, and in large parts of central Anatolia has also been suggested by palynologists as a possible outcome of direct anthropogenic deforestation with the additional effect of climate as a stressor (Bottema et al., 1986; Kaniewski et al., 2007). Thus, the simulated encroachment of woody species into grassland areas and the potential increase in woody biomass by 2100, sans human interference, suggest anthropogenic interference as a continuous primary disturbance parameter limiting the expansion of forested areas in this region.

## AUTHOR CONTRIBUTIONS

**Bikem Ekberzade:** Conceptualization (lead); data curation (lead); formal analysis (lead); funding acquisition (equal); investigation (lead); methodology (lead); project administration (lead); resources (equal); software (lead); validation (lead); visualization (lead); writing – original draft (lead); writing – review and editing (lead). **Omer Yetemen:** Conceptualization (supporting); formal analysis (supporting); funding acquisition (equal); resources (equal); supervision (supporting); writing – review and editing (supporting). **Yasemin Ezber:** Data curation (supporting); writing – review and editing (supporting). **Omer Lutfi Sen:** Conceptualization (supporting); formal analysis (supporting); methodology (supporting); supervision (supporting); writing – review and editing (supporting). **Hasan Nuzhet Dalfes:** Conceptualization (supporting); project administration (supporting); supervision (supporting); writing – review and editing (supporting).

## FUNDING INFORMATION

This study has been produced benefiting in part from the 2232 International Fellowship for Outstanding Researchers Program of the Scientific and Technological Research Council of Turkey (TUBITAK) through Grant 118C329. The financial support received from TUBITAK does not mean that the content of the publication is approved in a scientific sense by TUBITAK. Bikem Ekberzade's site visits and research are financed under ITU‐BAP grant number MDK‐2023‐44546, and TUBITAK Grant 123O248. Computing resources used in this work were provided by the National Center for High Performance Computing (UHeM) in Turkey, under Grant Number 1007482020.

## CONFLICT OF INTEREST STATEMENT

The authors have no relevant financial or non‐financial conflict of interest to disclose.

### OPEN RESEARCH BADGES

This article has earned an Open Data badge for making publicly available the digitally‐shareable data necessary to reproduce the reported results. The data is available at https://datadryad.org/stash/share/obPEH_nK83mDPID_owW72bu9A7RuTzUlUeQvObAFnPk and https://gitfront.io/r/user‐8458414/Z4xgC9HKUunL/LPJ‐GUESS‐OutputPlots/.

## Supporting information


Appendix S1

## Data Availability

All simulation results from different climate datasets used for final results analyses and their ensemble median, including the instruction file used in model simulations are kept on Dryad at the following link: https://datadryad.org/stash/share/obPEH_nK83mDPID_owW72bu9A7RuTzUlUeQvObAFnPk. The R code used in all analyses can be accessed both through the dataset or at GitHub: https://gitfront.io/r/user‐8458414/Z4xgC9HKUunL/LPJ‐GUESS‐OutputPlots/. Use of the simulation results is open and free, with proper citing of this manuscript. The raw form of climate and atmospheric data used in this research is open source and freely available to download from original sources cited in Appendix S1 (links also provided in Dryad/README.md).

## APPENDIX 1

**TABLE A1:** Climatic limits for the PFTs used in the simulations.

Species/PFT	Tcmin^s^ (°C)	Tcmin^e^ (°C)	Tcmax^e^ (°C)	Twmin (°C)	GDD5_min_ (°C)	Root distribution	Drought tolerance	Shade tolerance	Growth form	Phenology	Bioclimatic zone
*Abies* spp.^a^	−8	−7	0	10	1200	0.7 0.3	0.25	Tolerant	Tree	Evergreen	Temperate
*Alnus glutinosa*	−20	−20	3	5	600	0.8 0.2	0.4	Intermediate	Tree	Summergreen	Temperate
*Betula pendula*	−30	−30	7	5	700	0.8 0.2	0.42	Intolerant	Tree	Summergreen	Temperate
*Betula pubescens*	−30	−30	3	5	350	0.8 0.2	0.45	Intolerant	Tree	Summergreen	Boreal
*Carpinus* spp.^b^	−8	−8	5	5	1100	0.8 0.2	0.25	Intermediate	Tree	Summergreen	Temperate
*Cedrus libani*	−9	−8	2	16	2200	0.6 0.4	0.05	Intolerant	Tree	Evergreen	Temperate
*Corylus avellana*	−11	−11	7	5	800	0.8 0.2	0.3	Intermediate	Shrub	Summergreen	Temperate; k_allom1 250
*Fagus* spp.^b^	−3.5	−3.5	6	5	1500	0.8 0.2	0.2	Tolerant	Tree	Summergreen	Temperate
*Fraxinus excelsior*	−16	−16	6	5	1100	0.8 0.2	0.4	Intermediate	Tree	Summergreen	Temperate
*Juniperus oxycedrus*	0	1	1000	−1000	2200	0.5 0.5	0.01	Intolerant	Shrub	Evergreen	Temperate
*Picea* spp.^b^	−30	−30	−1.5	5	600	0.9 0.1	0.43	Tolerant	Tree	Evergreen	Boreal
*Pinus brutia*	−2.5	−2	9	−1000	1500	0.6 0.4	0.05	Intolerant	Tree	Evergreen	Temperate
*Pinus halepensis*	3	3	9	21	3000	0.6 0.4	0.05	Intolerant	Tree	Evergreen	Temperate
*Pinus nigra*	−8	−7	5	10	1250	0.6 0.4	0.1	Intolerant	Tree	Evergreen	Temperate
*Pinus sylvestris*	−30	−30	−1	5	500	0.6 0.4	0.25	Intermediate	Tree	Evergreen	Boreal
*Populus tremula*	−31	−30	6	−1000	500	0.7 0.3	0.4	Intolerant	Tree	Summergreen	Temperate
*Quercus coccifera*	0	0	11	21	2200	0.5 0.5	0.1	Intermediate	Shrub	Evergreen	Temperate; k_allom1 250
*Quercus ilex*	3	3	7	5	1800	0.5 0.5	0.2	Intermediate	Tree	Evergreen	Temperate
*Quercus macranthera*	−16	−15	5	−1000	900	0.6 0.4	0.175	Intolerant	Tree	Summergreen	Temperate
*Quercus pubescens*	−6	−5	6	−1000	1900	0.6 0.4	0.175	Intermediate	Tree	Summergreen	Temperate
*Quercus robur*	−10	−9	6	5	1100	0.6 0.4	0.25	Intermediate	Tree	Summergreen	Temperate
*Tilia cordata*	−12	−11	5	5	1100	0.8 0.2	0.33	Intermediate	Tree	Summergreen	Temperate
*Ulmus glabra*	−10.5	−9.5	6	5	850	0.6 0.4	0.4	Intermediate	Tree	Summergreen	Temperate
Boreal evergreen shrub	−1000	−1000	−1	−1000	200	0.8 0.2	0.25	Intermediate	Shrub	Evergreen	Boreal; k_allom1 250
Mediterranean raingreen shrub	0	1	1000	−1000	2200	0.9 0.1	0.01	Intolerant	Shrub	Raingreen	Temperate; k_allom1 250
C3 herbaceous	−1000	−1000	1000	−1000	0	0.9 0.1	0.01	–	–	–	

*Note*: *Alnus glutinosa*, *Cedrus libani*, *Pinus brutia*, *P. nigra*, and *Quercus macranthera* were added, and the bioclimatic limits of all taxa from the default European species list have been calibrated with considerations for Sitch et al. (2003), Sykes et al. (1996), Hickler et al. (2012) and Ekberzade et al. (2022); through literature review, and expert opinion. Tcmin^s^ and Tcmin^e^ indicate minimum temperature of the coldest month for survival and establishment, respectively; Tcmax^e^, maximum temperature for establishment of the coldest month; and Twmin, minimum temperature of the warmest month.

^a^ Including *A. nordmanniana*, *A. bornmülleria*, and *A. borisiiregis* and in limited capacity *A. cilicica*.

^b^ *Fagus* spp. including *F. sylvatica* and *F. orientalis*; *Carpinus* spp. including *C. betulus* and *C. orientalis*; *Picea* spp. Including *P. abies* and *P. orientalis*.

**TABLE A2:** To calibrate the respiration coefficients for boreal versus temperate taxa, we had to do an ensemble run, where we introduced incremental increases and decreases and plotted the results of species distribution.

Geographic range	Resp. coefficient
Boreal tree	1.5
Temperate tree	1

*Note*: As there had to be a differentiation between the different climatic groups (Patterson et al., 2018; Reich et al., 2016) and Hickler et al. (2012) values for boreal taxa rendered simulation results which were an overshoot to field observation and TGDF occurrence data for Turkey. A better fit was reached at 1.5 for boreal taxa while temperate taxa stayed at default. These parameters are the same as of those which we used for the previous version of the model in Ekberzade et al. (2022).
