# Supplementary material for: Latitude or altitude as the future refugium? A case for the future of forests in Asia Minor and its surroundings
Source: Ecol Evol. 2024 Apr 12;14(4):e11131. doi: 10.1002/ece3.11131 (PMC11009660; doi:10.1002/ece3.11131)
Supplement: Supplementary file 1 — Appendix S1 [file ECE3-14-e11131-s001.docx]

**Table S1.** The list of GCMs used in this study.

| GCM name | Time Period | Resolution (km) | Variant Label | Research Institution and references |
| --- | --- | --- | --- | --- |
| EC-Earth 3p-HR | 1950-2099 | 50 | r3i1p2f1 | The EC-Earth Consortium  (Haarsma et al. 2020) |
| CMCC-ESM2 | 1950-2100 | 100 | r1i1p1f1 | The Euro-Mediterranean Center on Climate Change  (Lovato et al. 2022) |
| INM-CM5 | 1950-2100 | 100 | r1i1p1f1 | The Russian Science Foundation  (Institute for Numerical Mathematics 2020; Volodin and Gritsun 2018) |
| MPI-ESM1-2-HR | 1950-2100 | 100 | r1i1p1f1 | Max Planck Institute  (Gutjahr et al. 2019) |
| NorESM2-MM | 1950-2100 | 100 | r1i1p1f1 | The Norwegian Climate Center  (Seland et al. 2020) |

**References:**

Gutjahr O, Putrasahan D, Lohmann K, Jungclaus JH, von Storch J-S, Brüggemann N, Haak H, and Stössel A (2019) Max Planck Institute Earth System Model (MPI-ESM1.2) for the High-Resolution Model Intercomparison Project (HighResMIP). Geosci. Model Dev., 12:3241–3281. <https://doi.org/10.5194/gmd-12-3241-2019>

Haarsma R, Acosta M, Bakhshi R, Bretonnière P-A, Caron L-P, Castrillo M, Corti S, Davini P, Exarchou E, Fabiano F, Fladrich U, Fuentes Franco R, García-Serrano J, von Hardenberg J, Koenigk T, Levine X, Meccia VL, van Noije T, van den Oord G, Palmeiro FM, Rodrigo M, Ruprich-Robert Y, Le Sager P, Tourigny E, Wang S, van Weele M, and Wyser K (2020) HighResMIP versions of EC-Earth: EC-Earth3P and EC-Earth3P-HR – description, model computational performance and basic validation. Geosci. Model Dev., 13:3507–3527. <https://doi.org/10.5194/gmd-13-3507-2020>

Seland Ø, Bentsen M, Olivié D, Toniazzo T, Gjermundsen A, Graff LS, Debernard JB, Gupta AK, He Y-C, Kirkevåg A, Schwinger J, Tjiputra J, Aas KS, Bethke I, Fan Y, Griesfeller J, Grini A, Guo C, Ilicak M, Karset IHH, Landgren O, Liakka J, Moseid KO, Nummelin A, Spensberger C, Tang H, Zhang Z, Heinze C, Iversen T, and Schulz M (2020) Overview of the Norwegian Earth System Model (NorESM2) and key climate response of CMIP6 DECK, historical, and scenario simulations. Geosci. Model Dev., 13:6165–6200. <https://doi.org/10.5194/gmd-13-6165-2020>

Institute for Numerical Mathematics (2020) WCRP CMIP6: Institute for Numerical Mathematics (INM) INM-CM5-0 model output for the "1pctCO2" experiment. Centre for Environmental Data Analysis. https://catalogue.ceda.ac.uk/uuid/5b50fd3fe7b74a64be91fb7ebb8c21a9 Accessed 12 Dec 2022.

Lovato T, Peano D, Butenschön M, Materia S, Iovino D, Scoccimarro E, et al. (2022) CMIP6 simulations with the CMCC Earth System Model (CMCC-ESM2). Journal of Advances in Modeling Earth Systems, 14, e2021MS002814. <https://doi.org/10.1029/2021MS002814>

Volodin E, Gritsun A (2018) Simulation of observed climate changes in 1850–2014 with climate model INM-CM5, Earth Syst. Dynam., 9, 1235–1242. <https://doi.org/10.5194/esd-9-1235-2018>


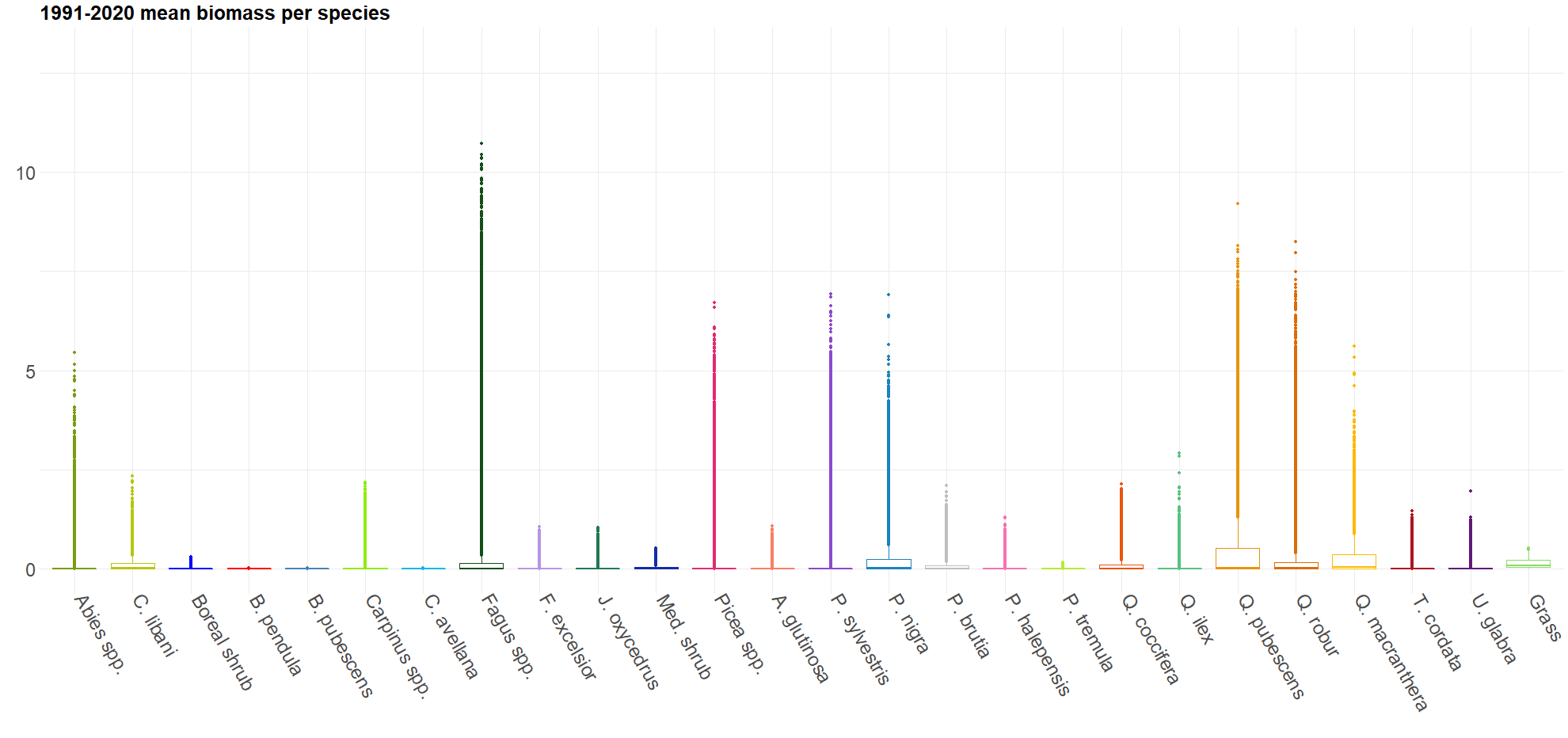

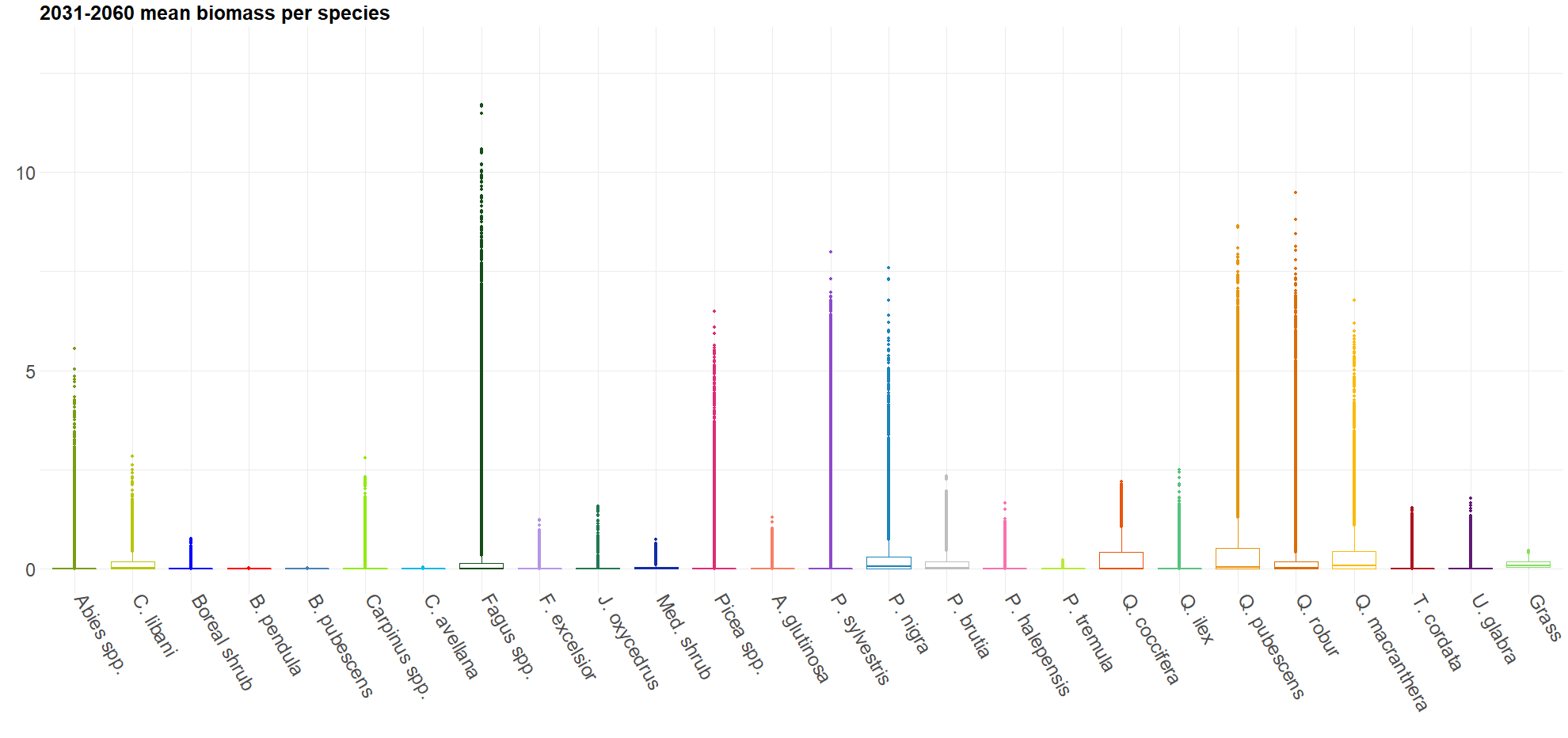

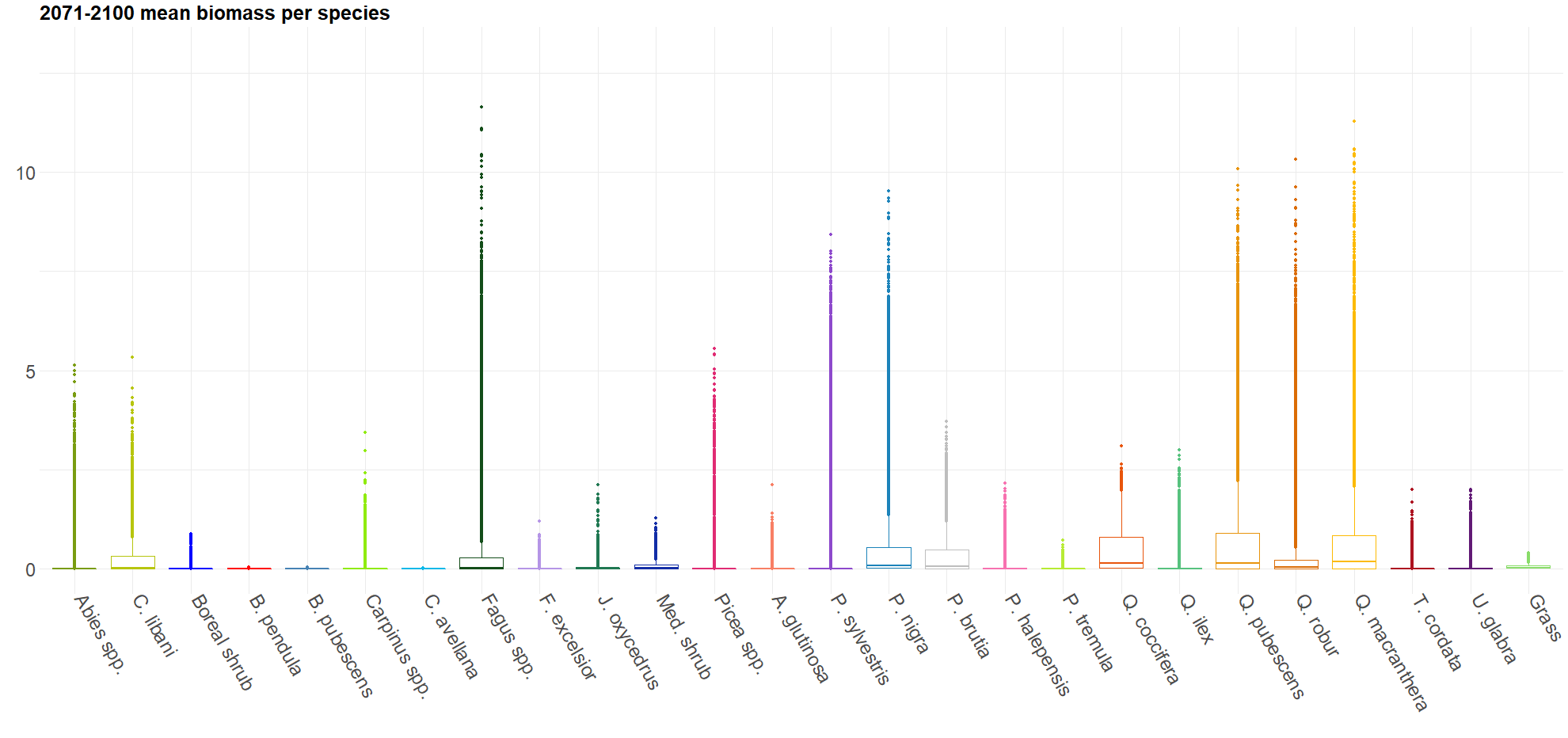

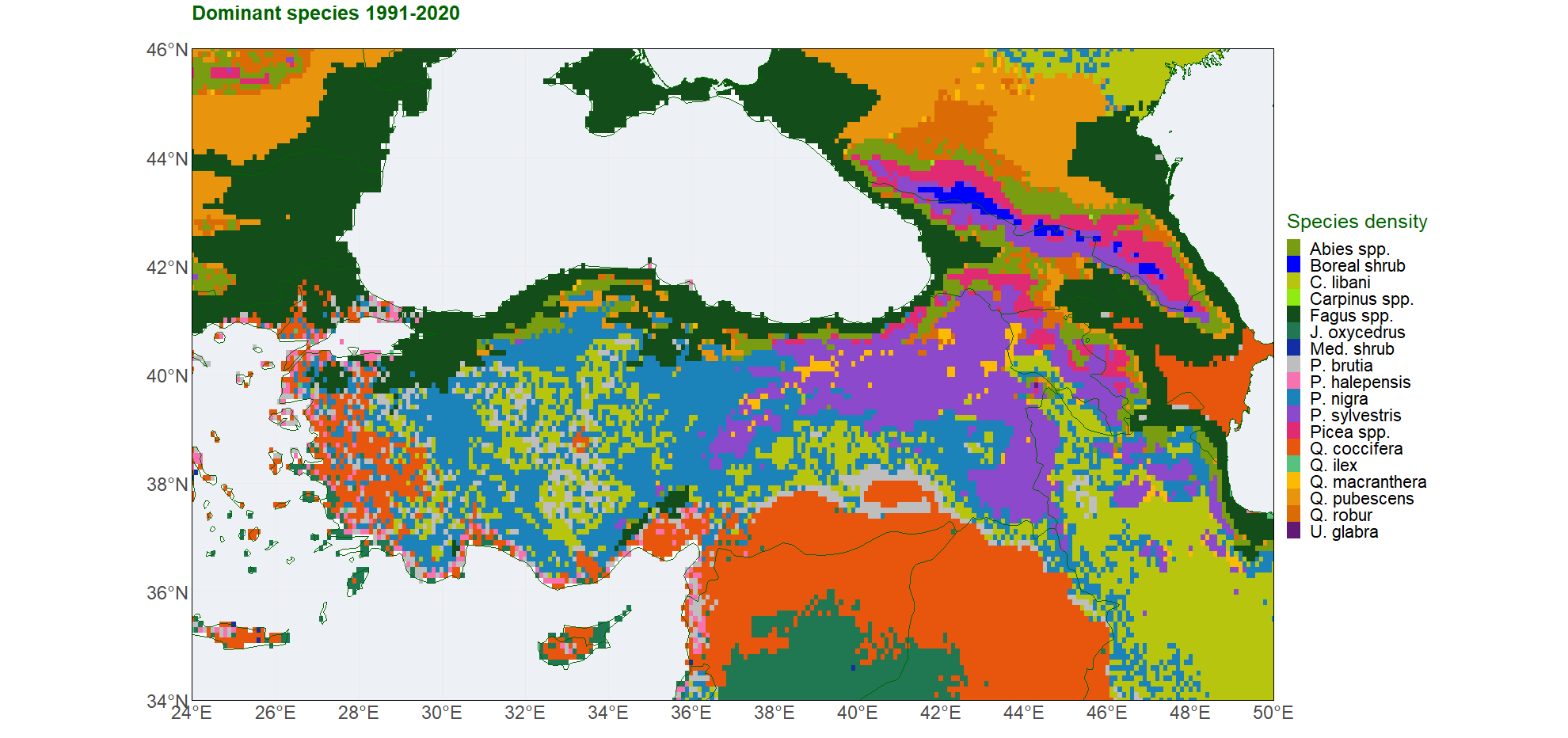


CMCC-ESM2


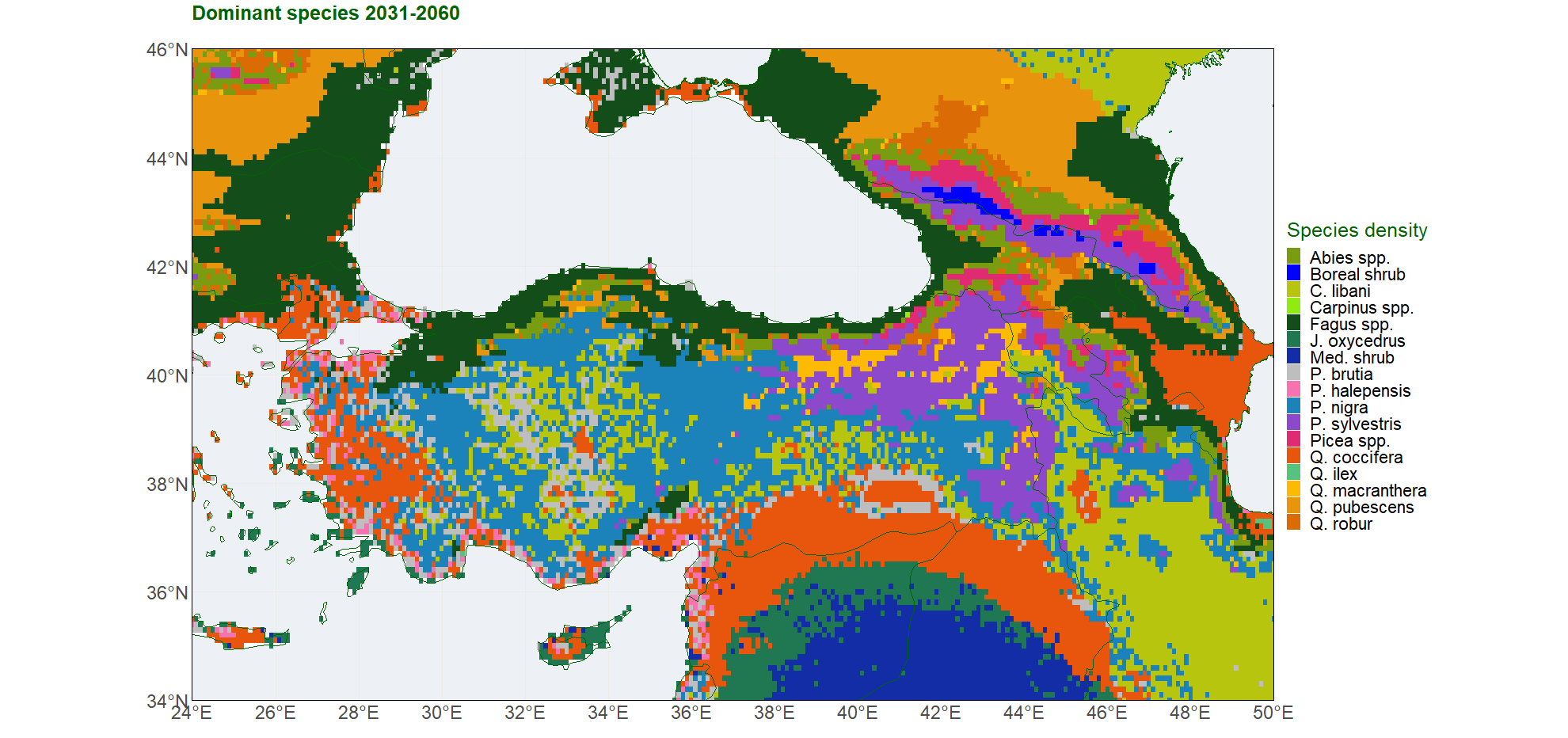

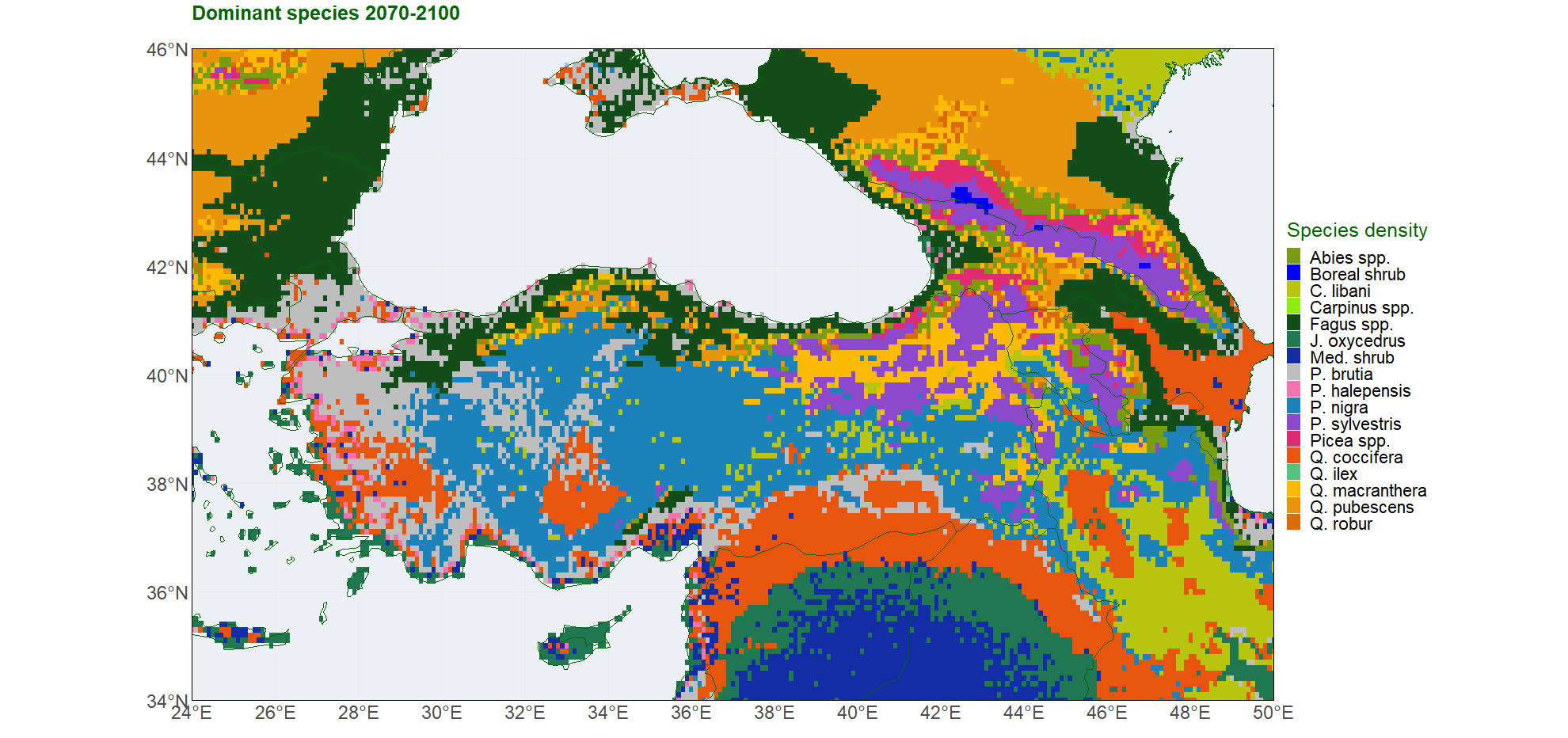

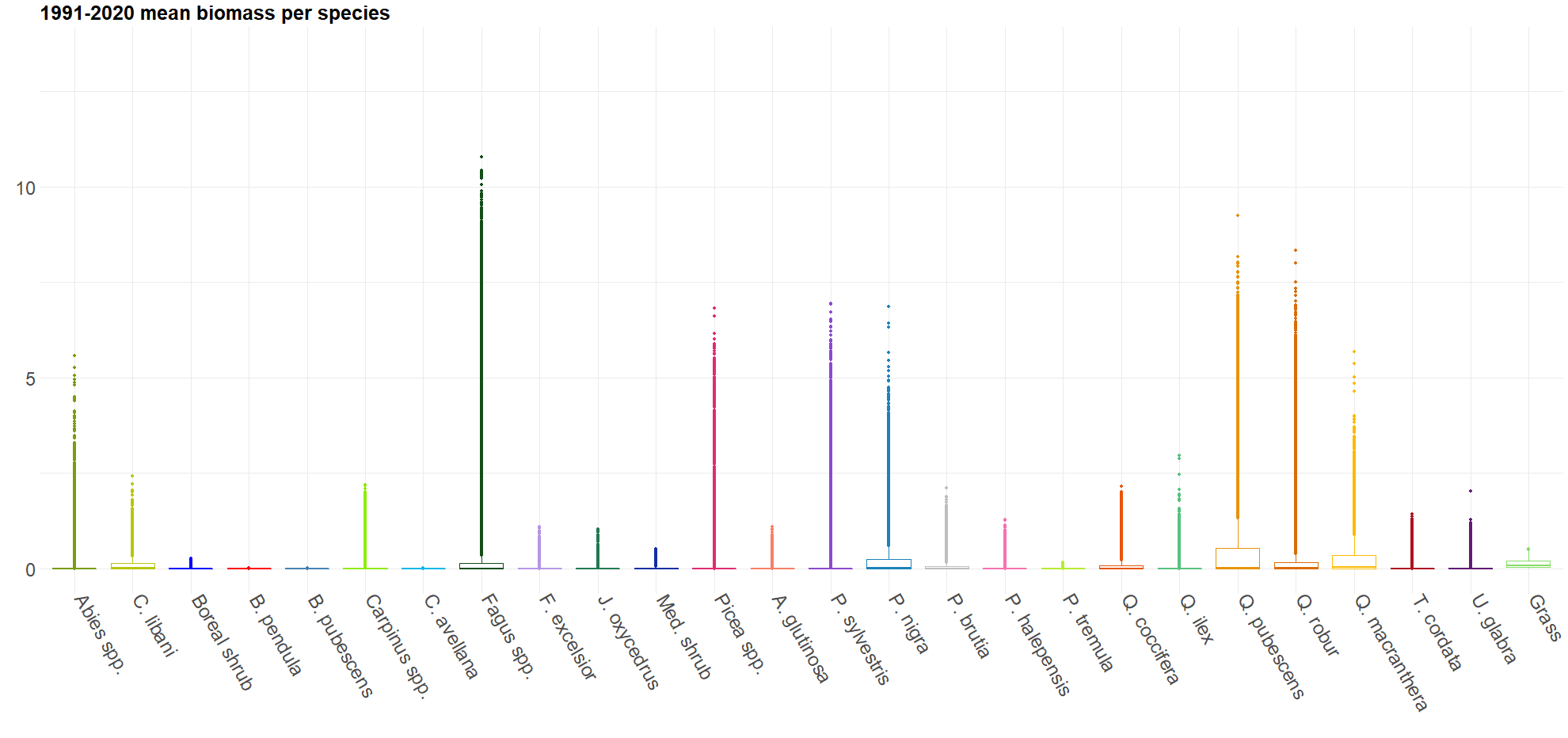

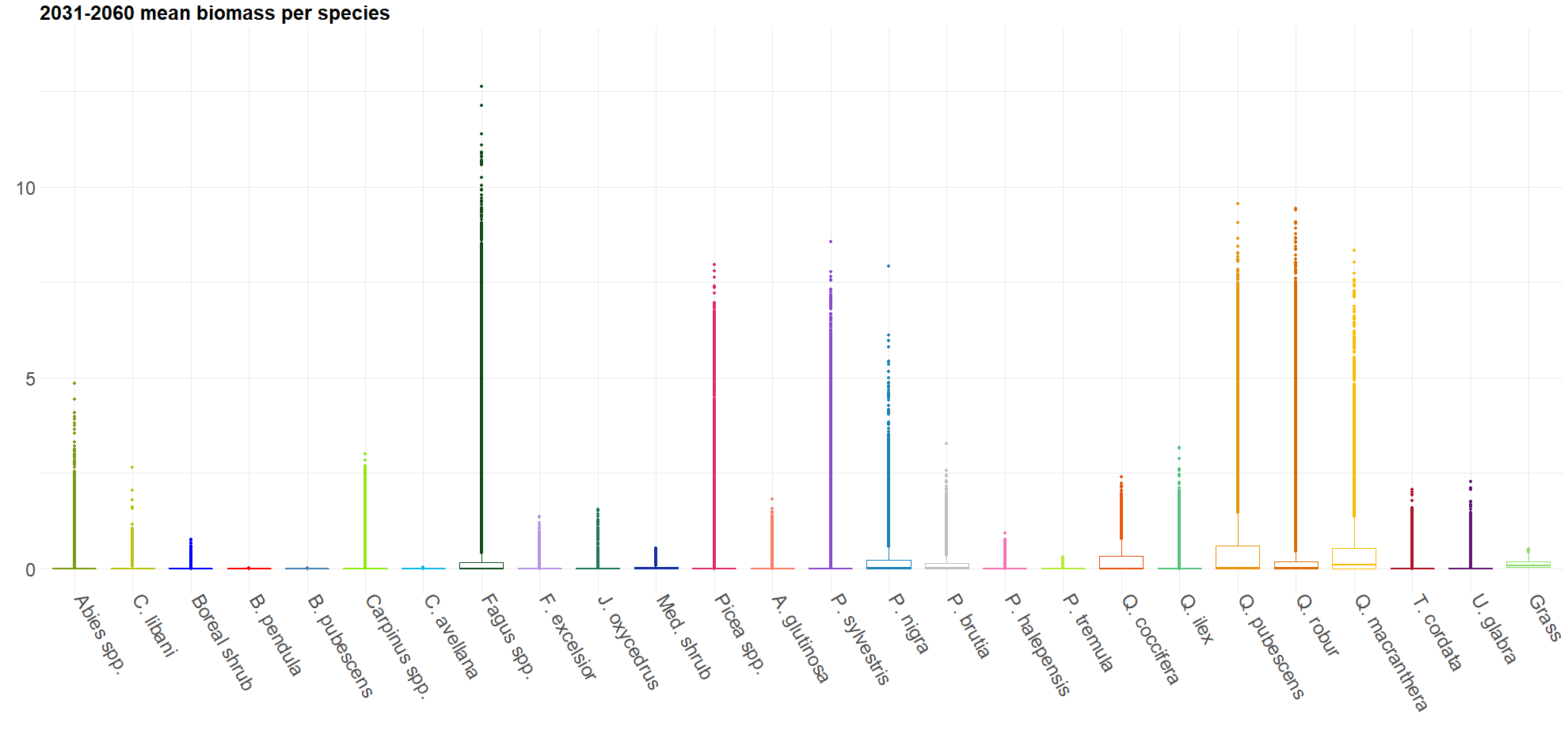

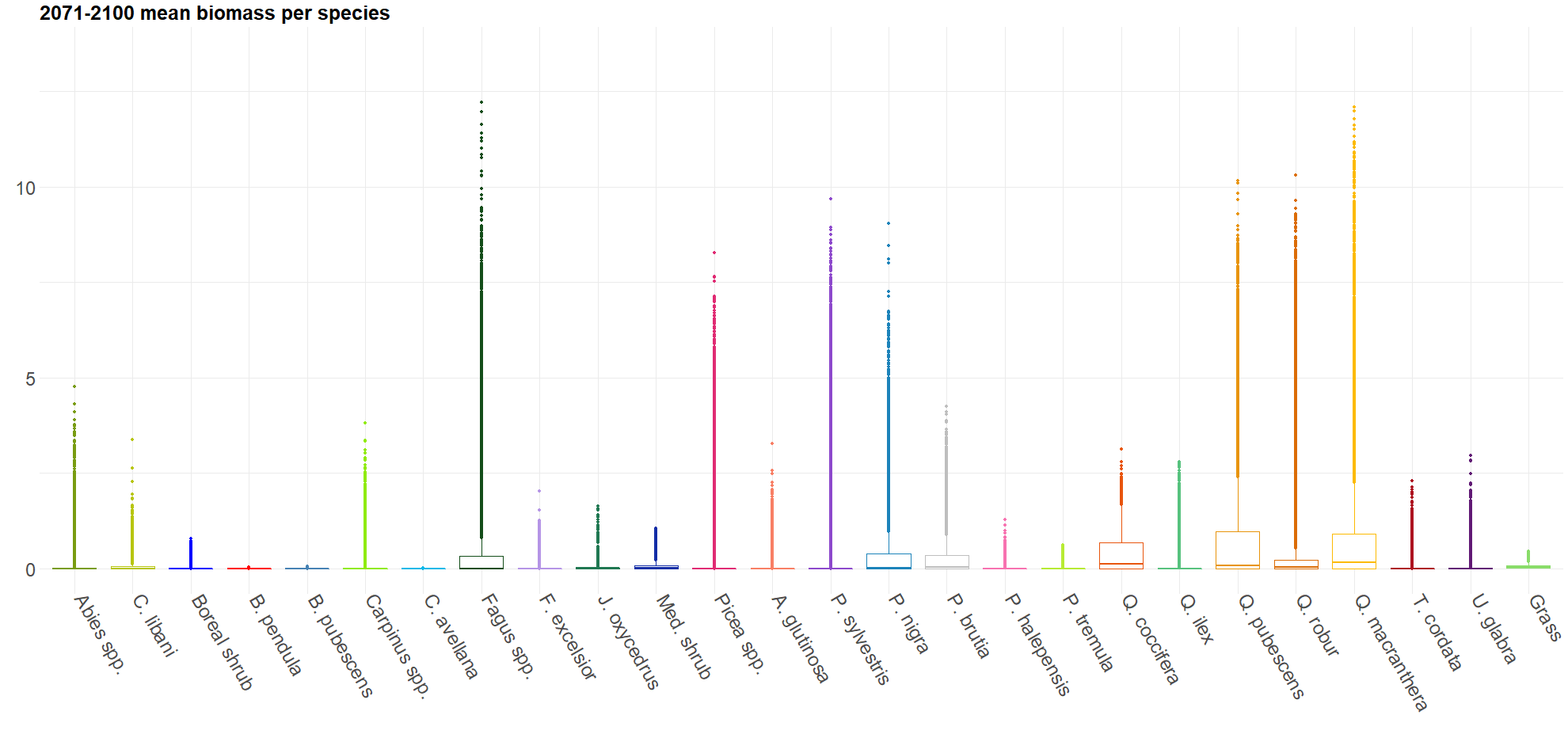

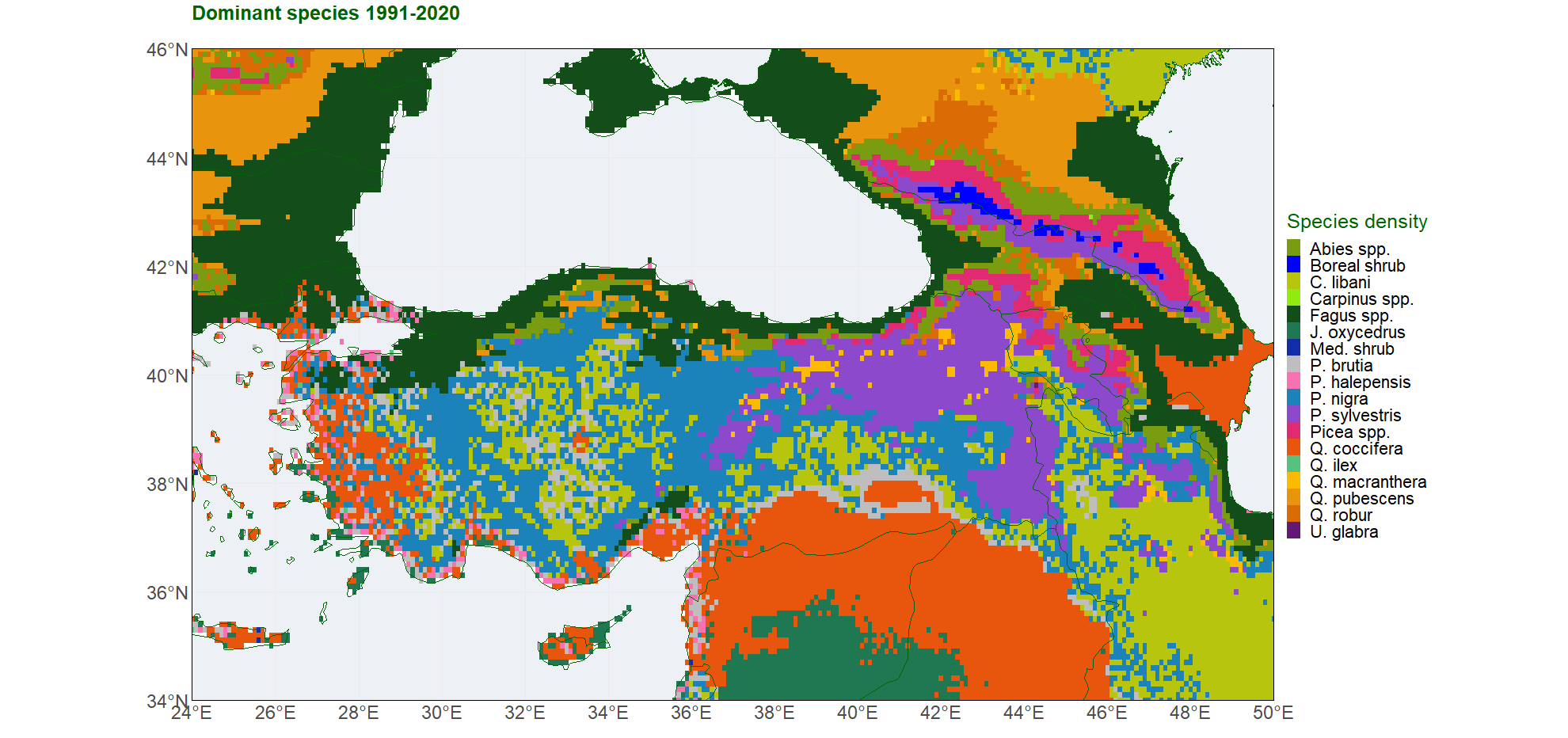

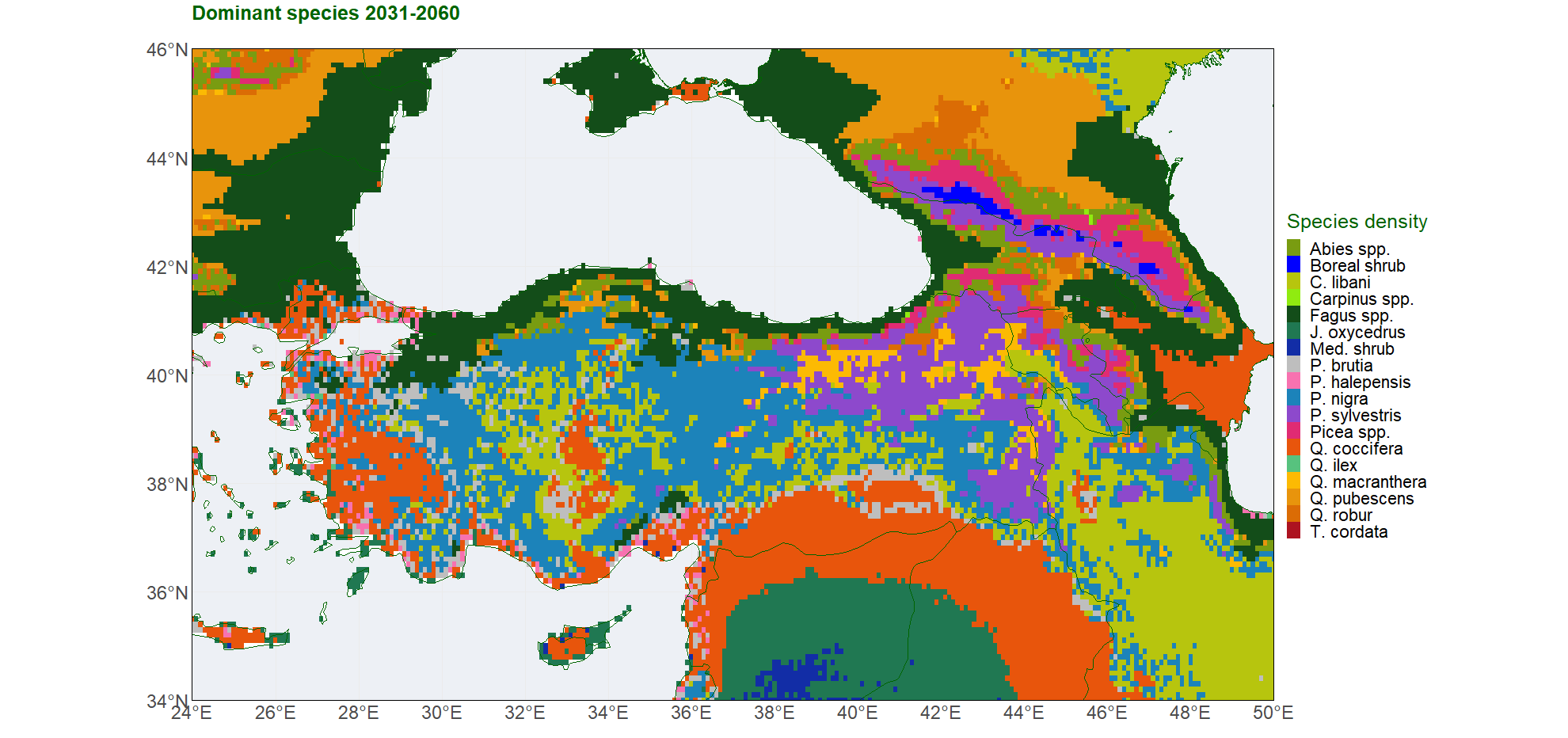

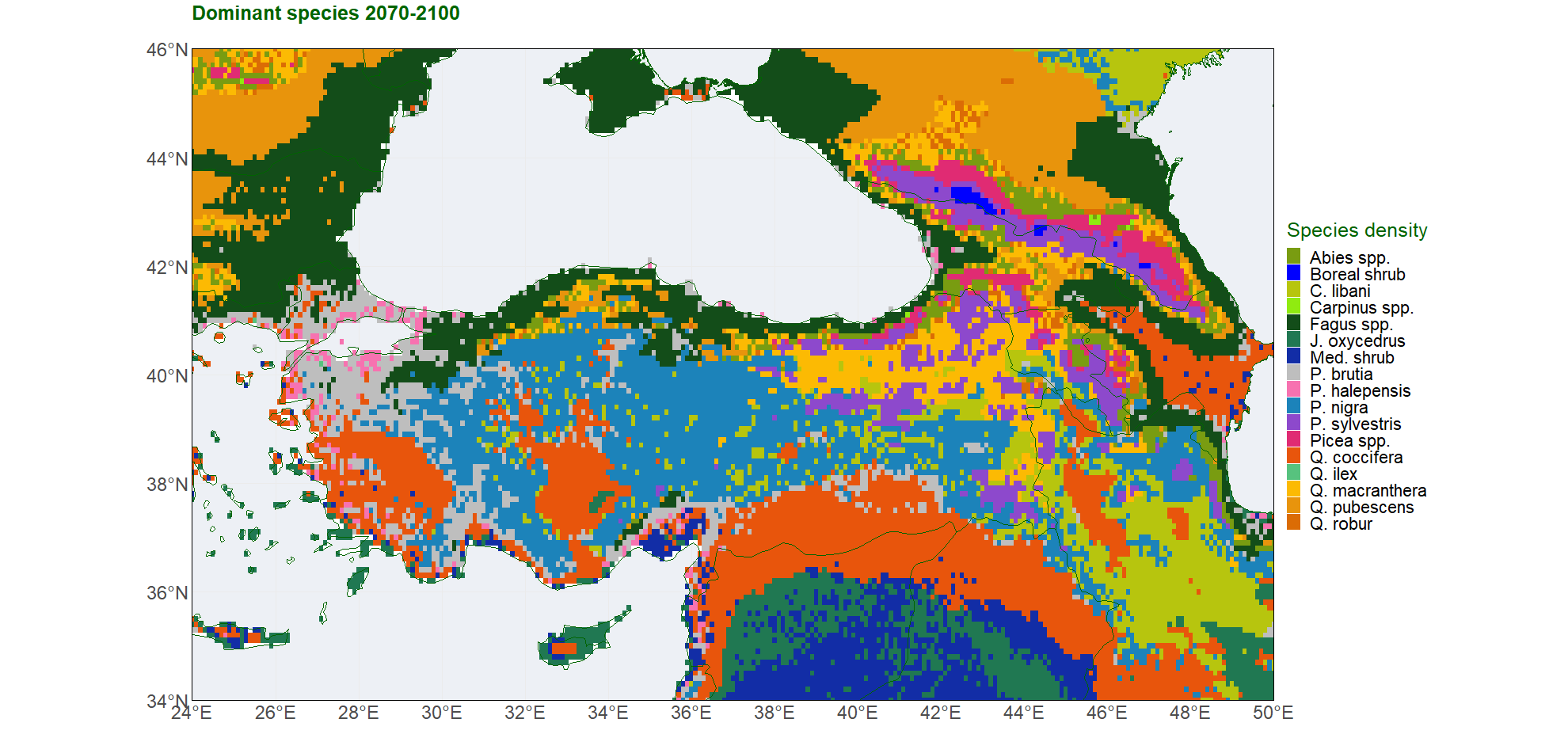


EC-Earth-3p-HR

# 1


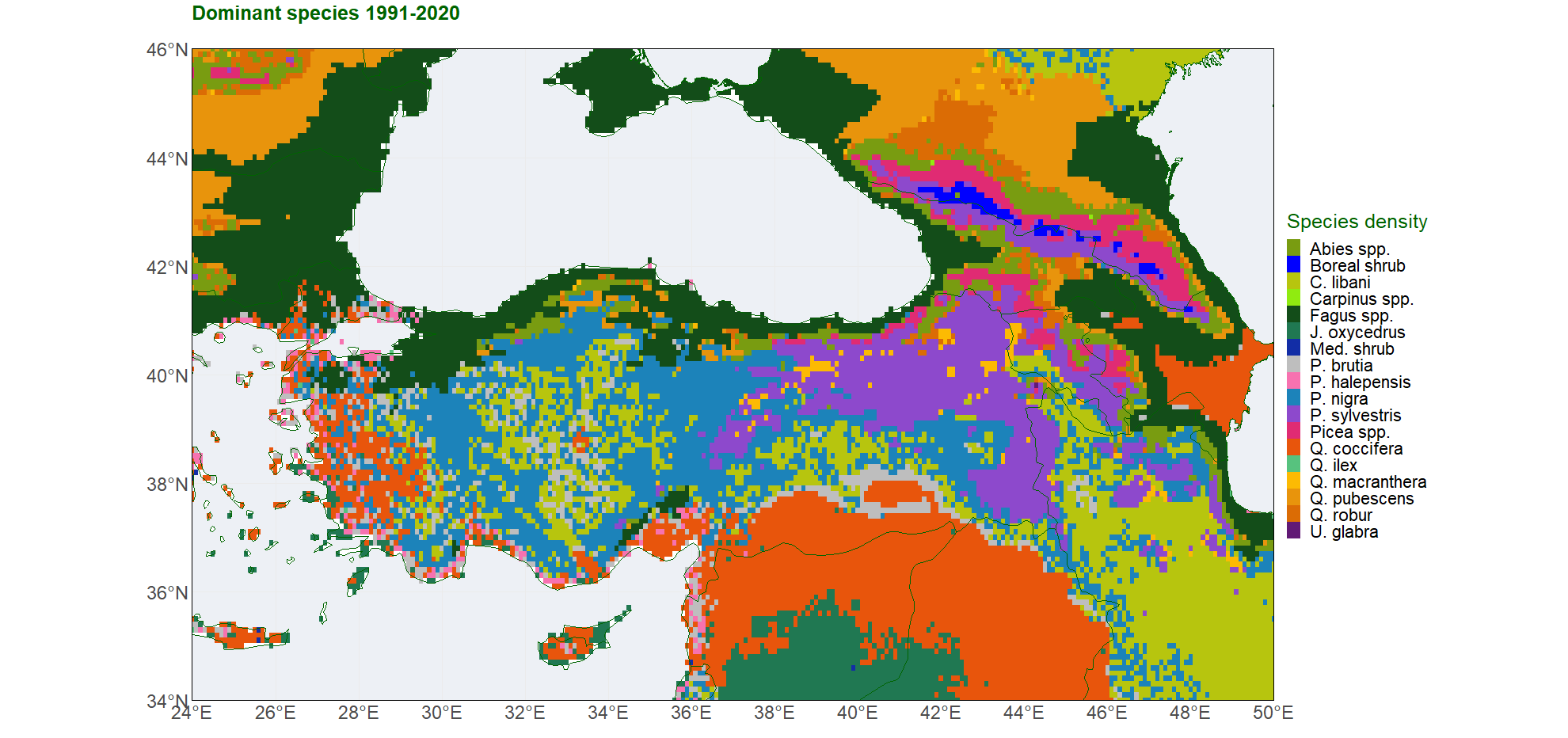

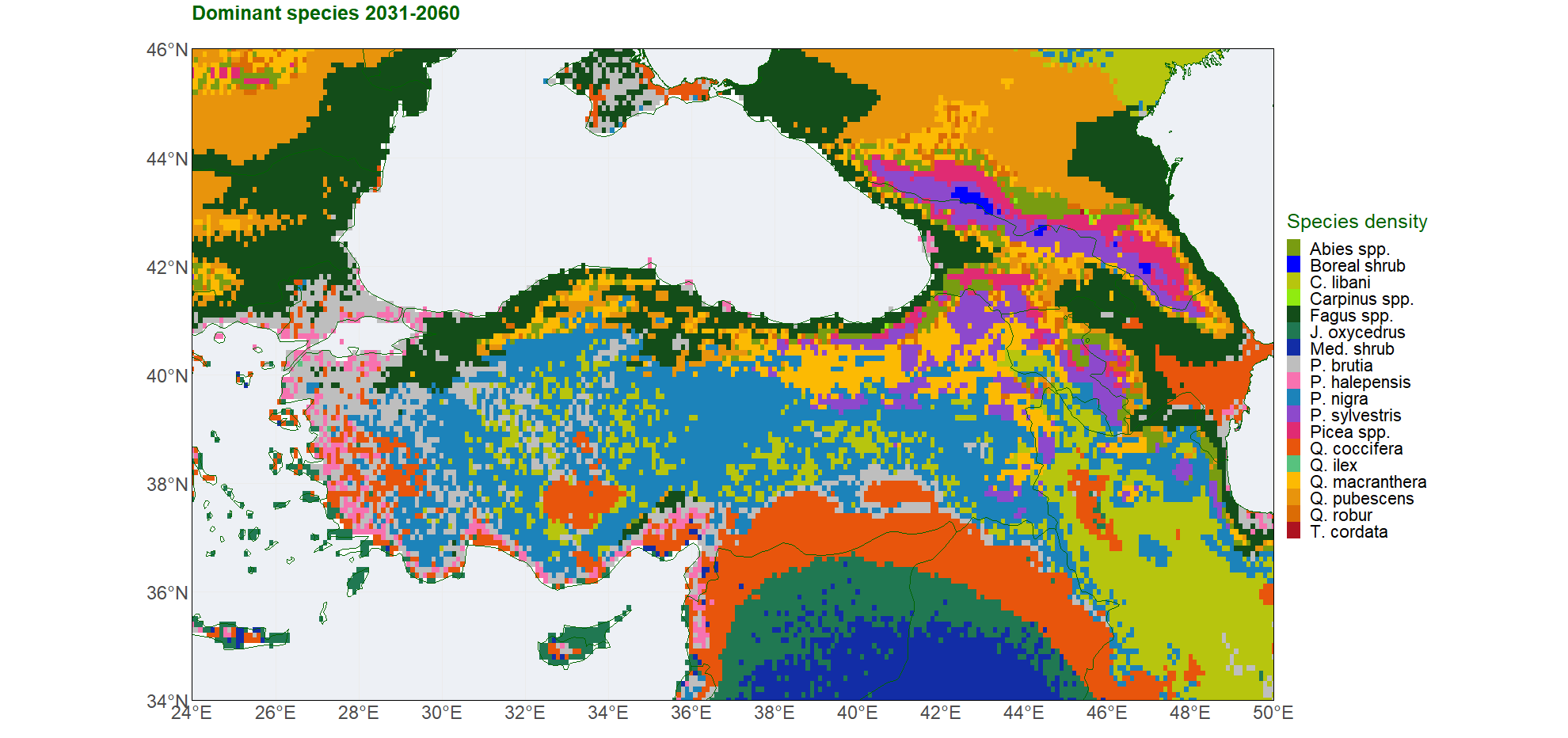

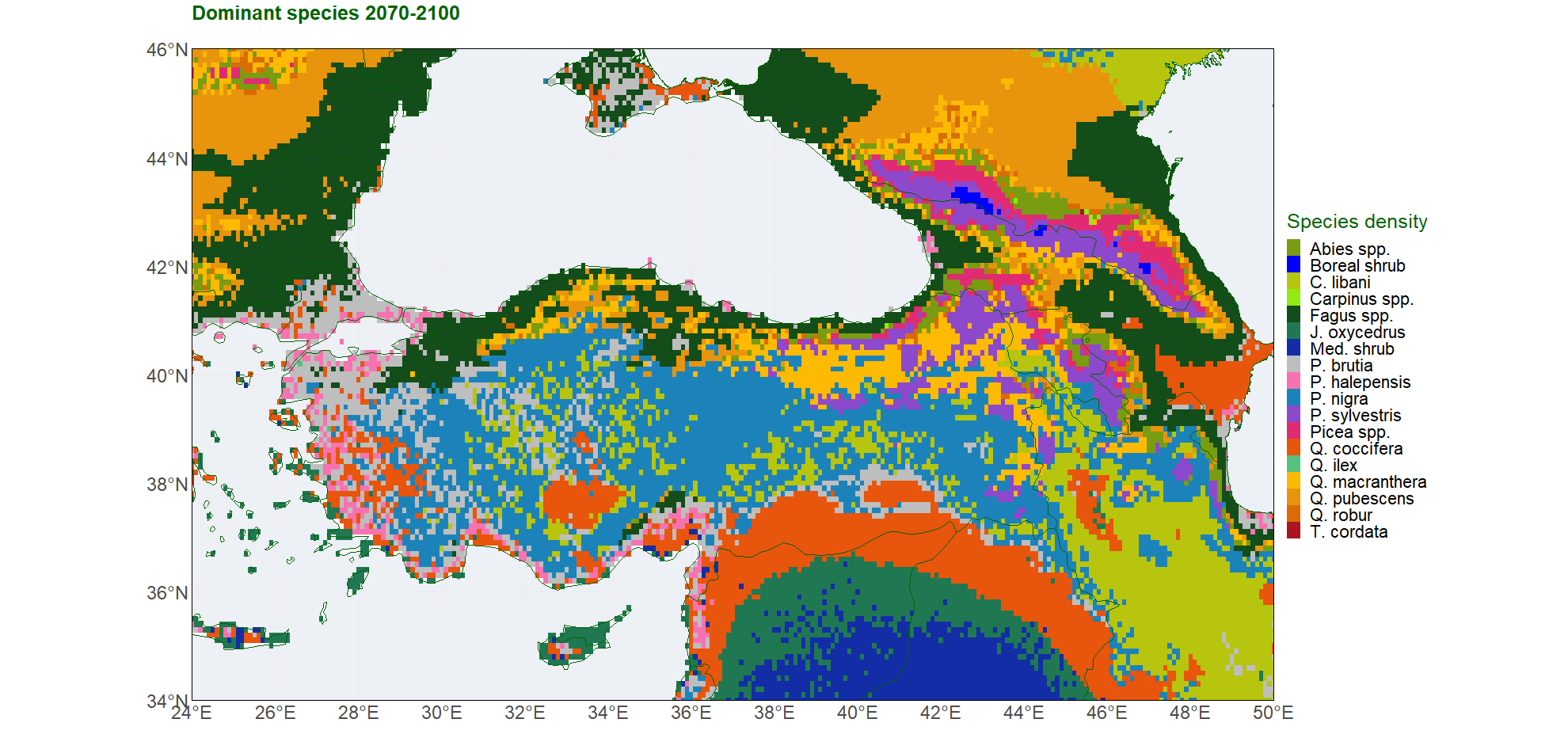

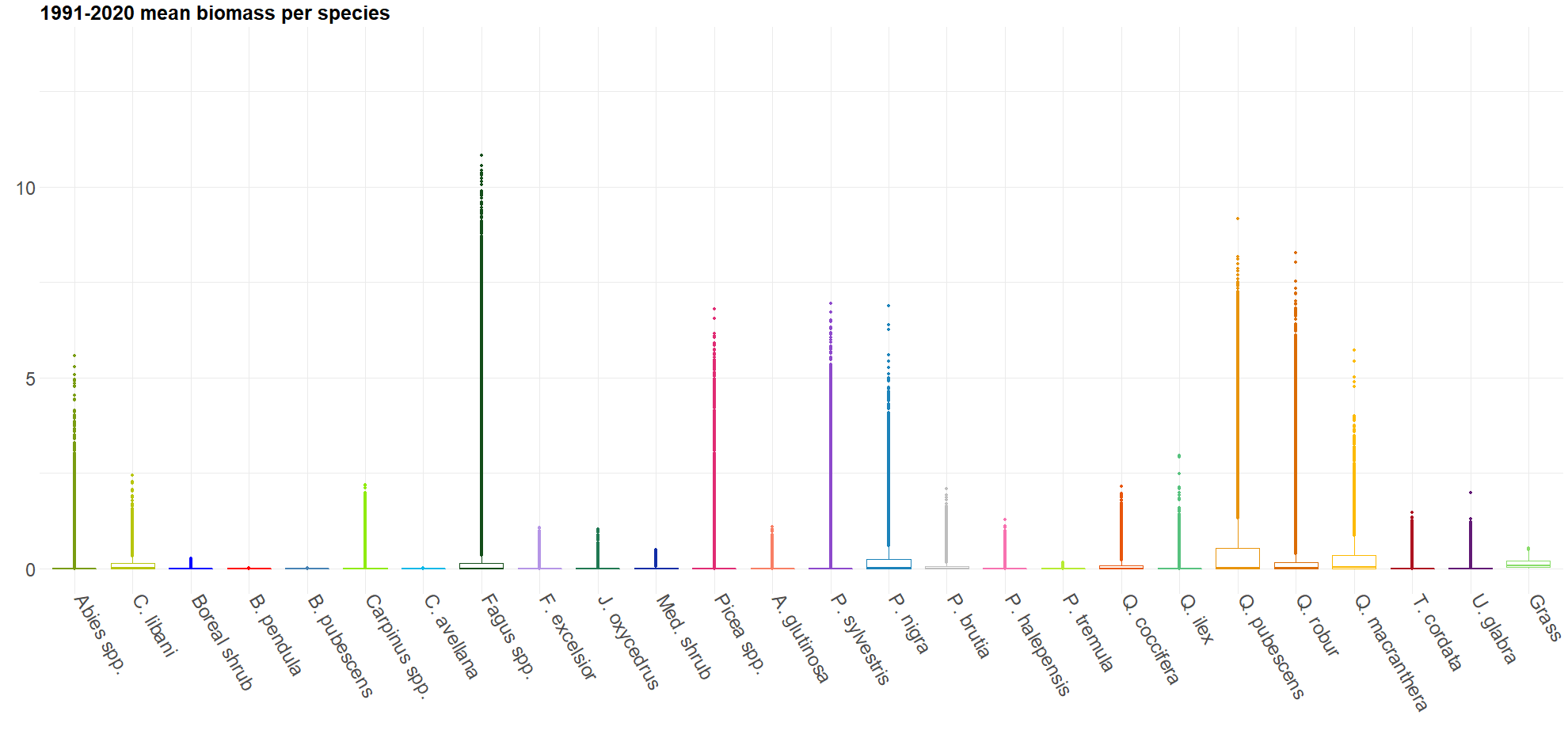

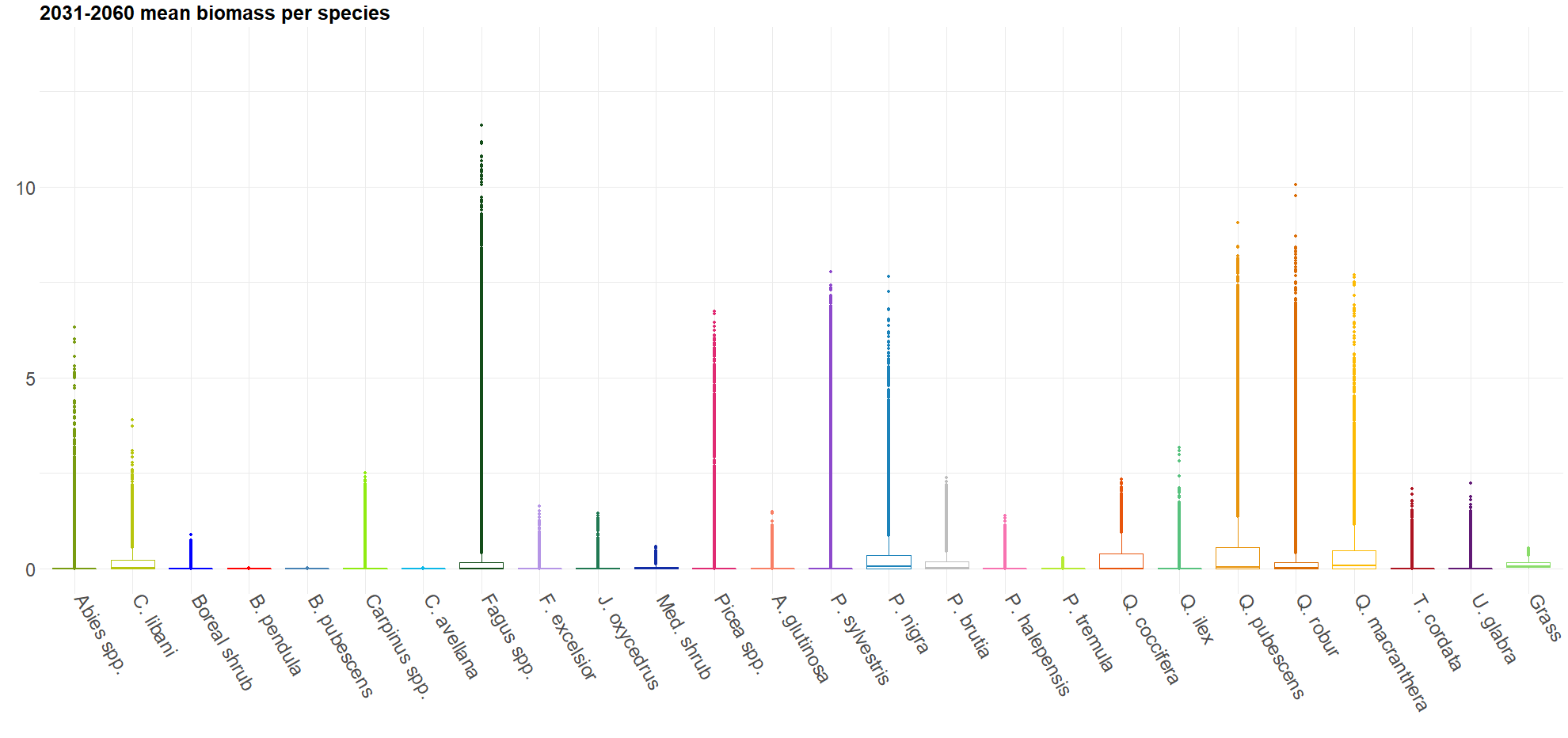

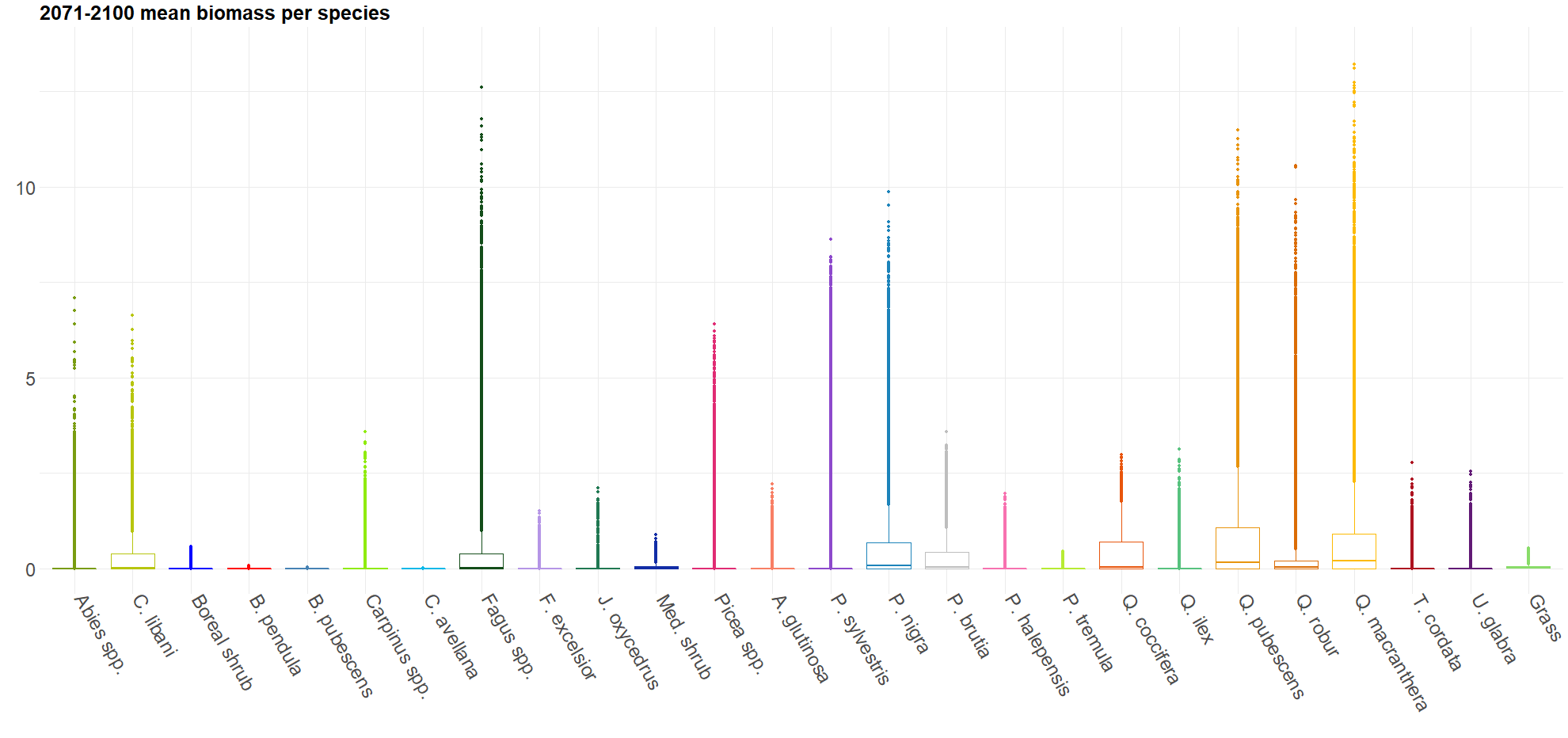


INM-CM5


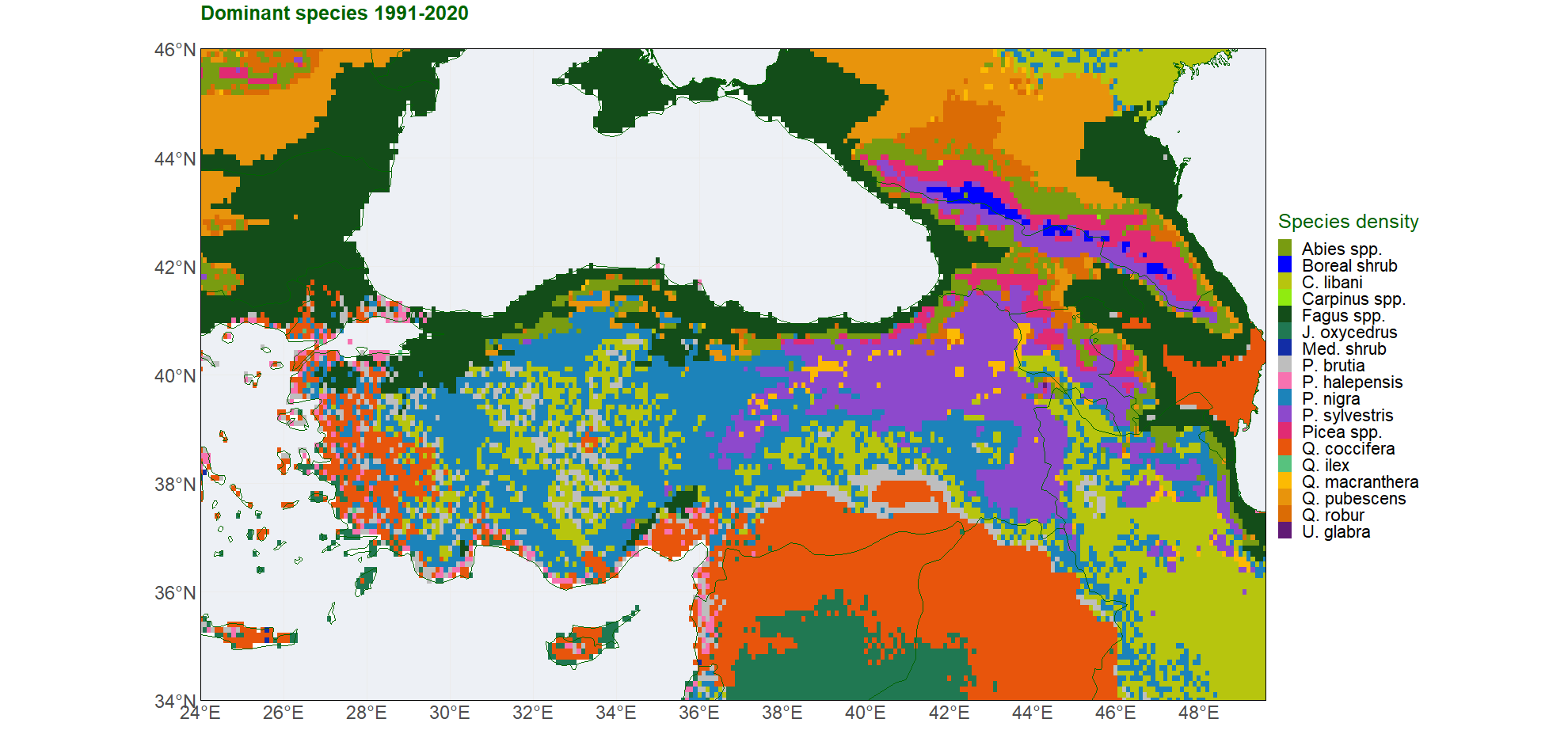

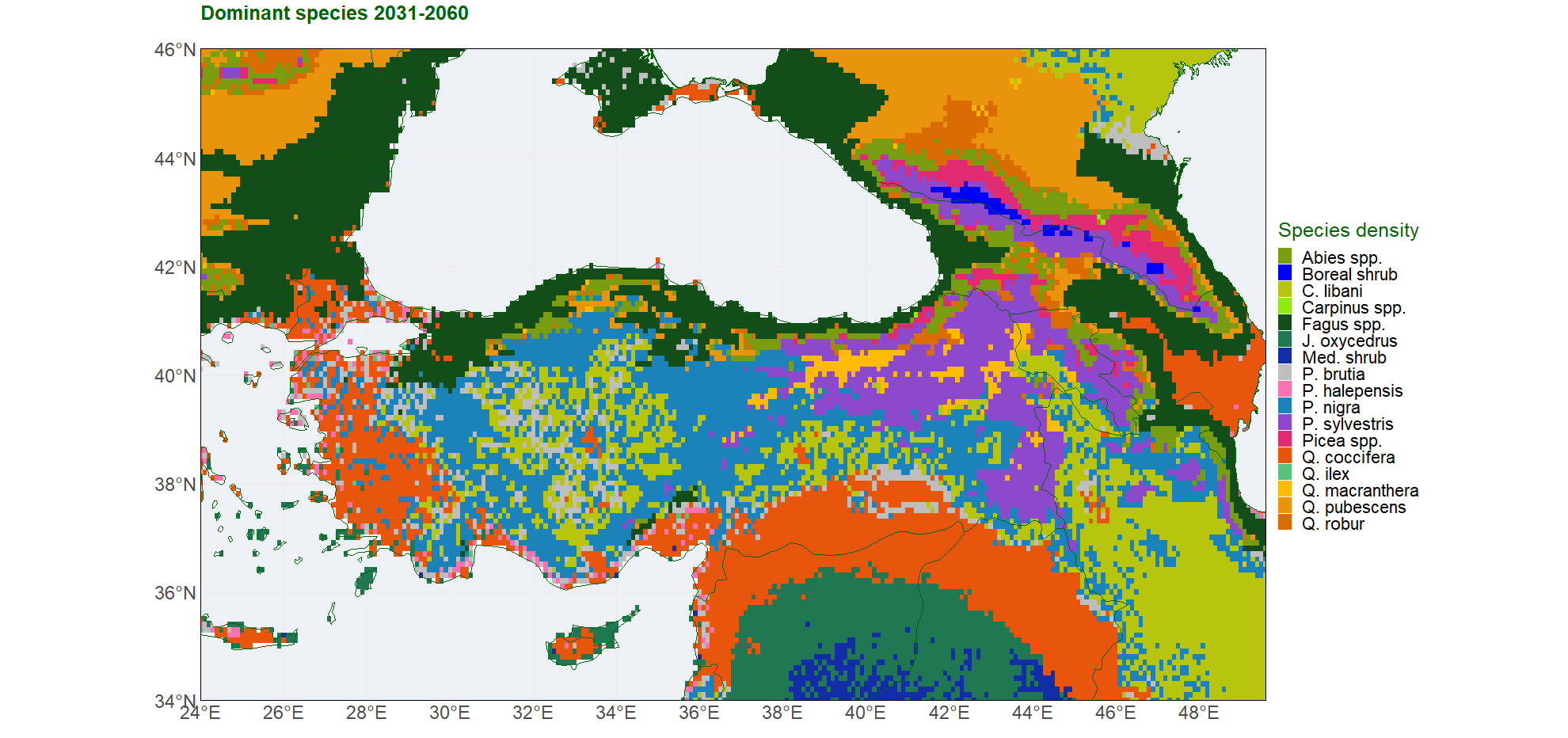

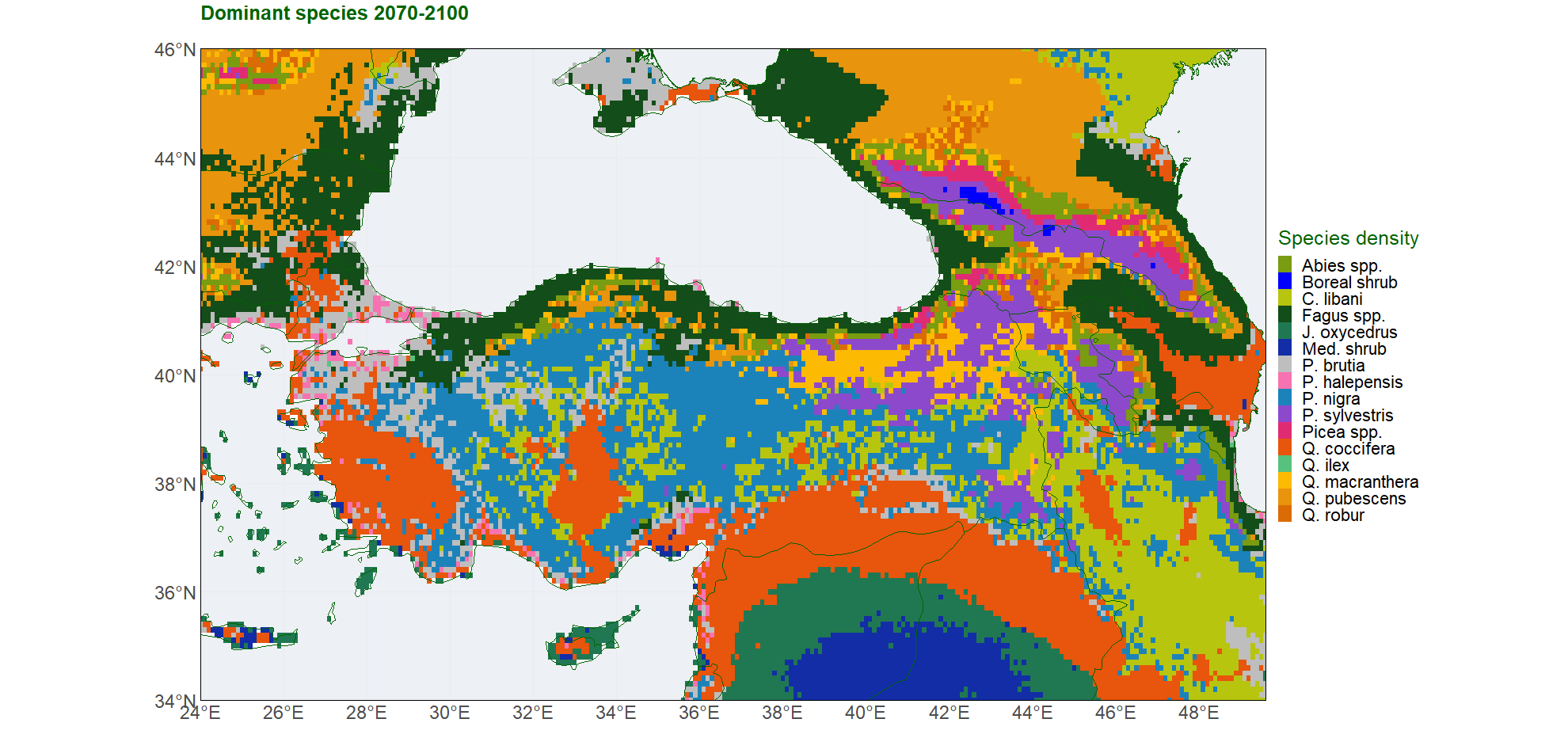

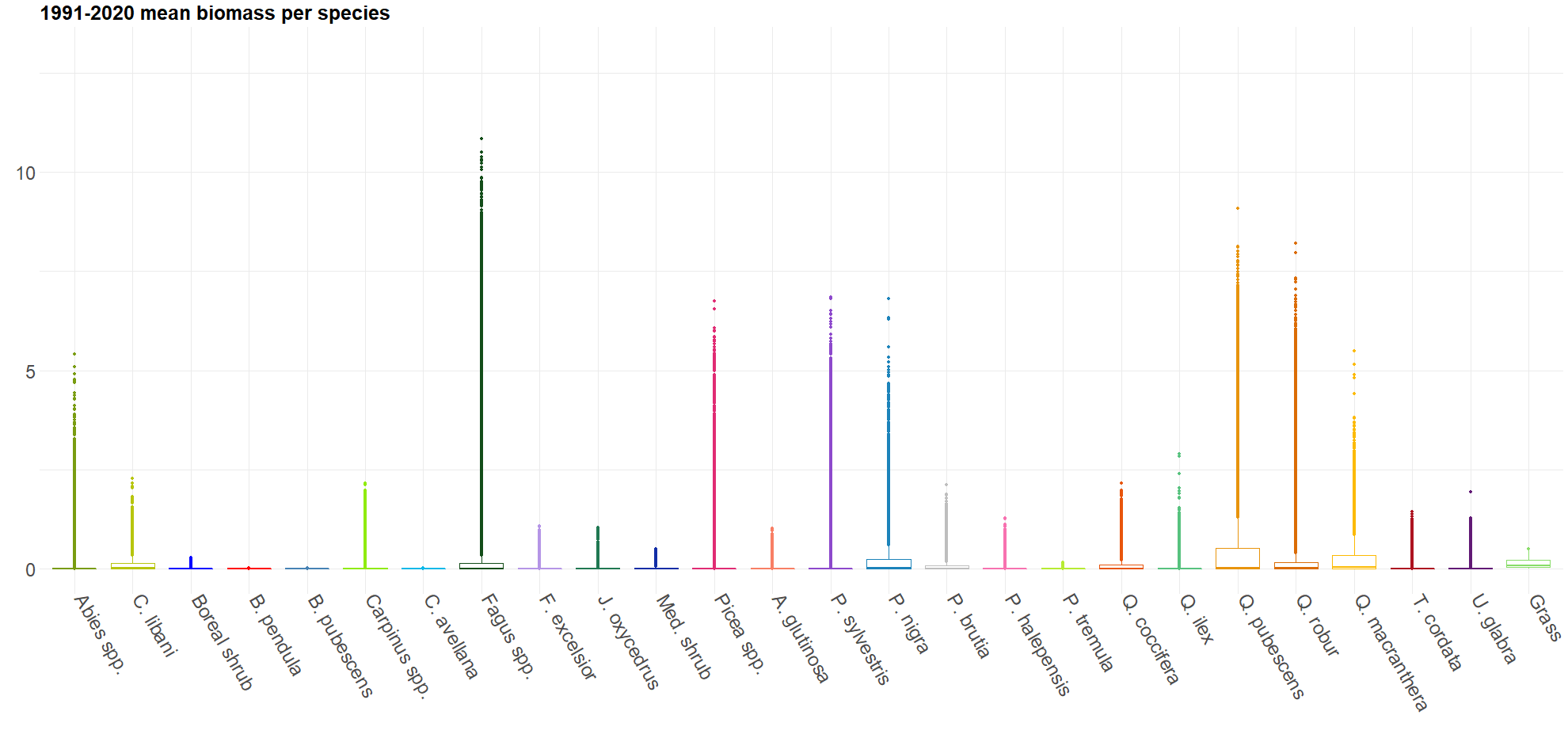

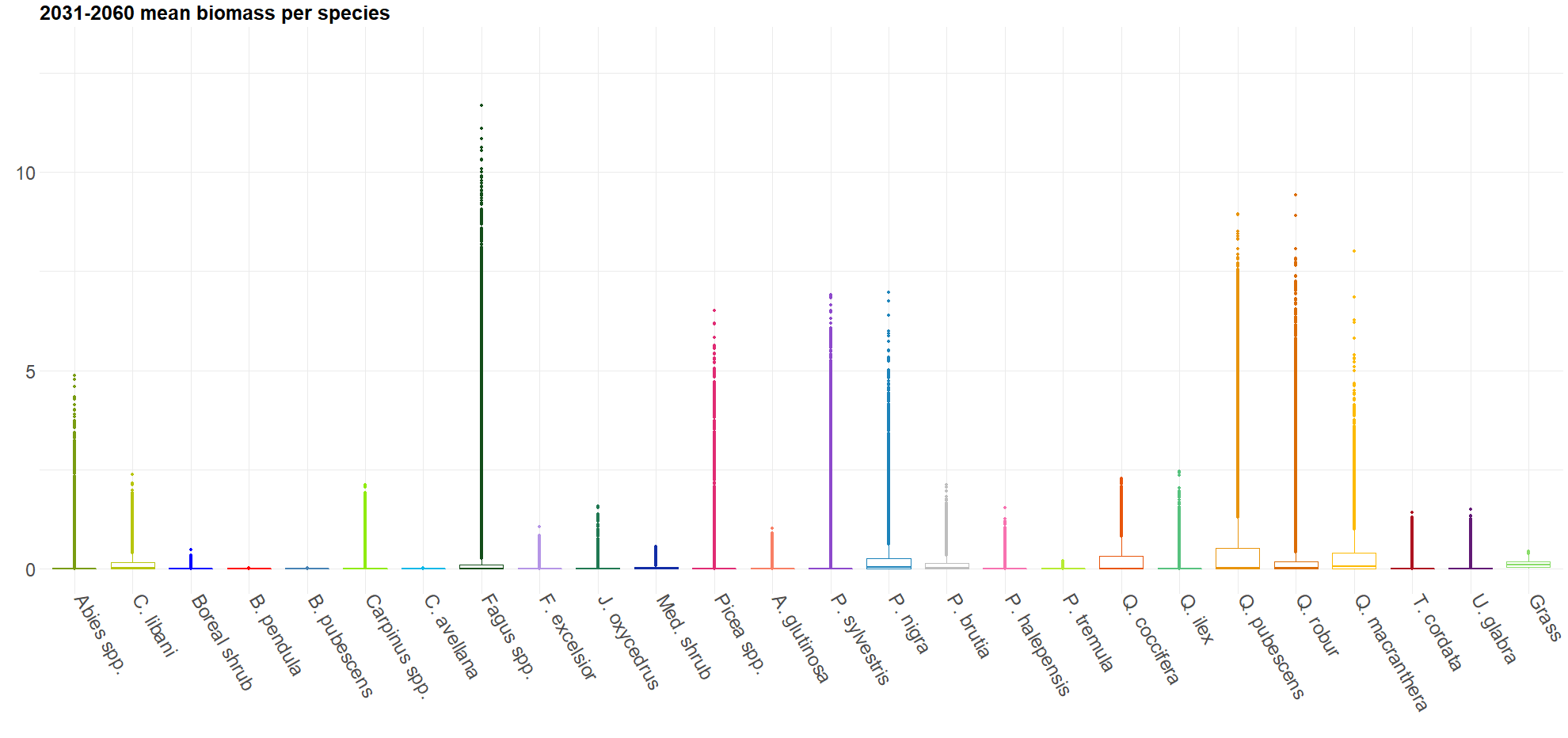

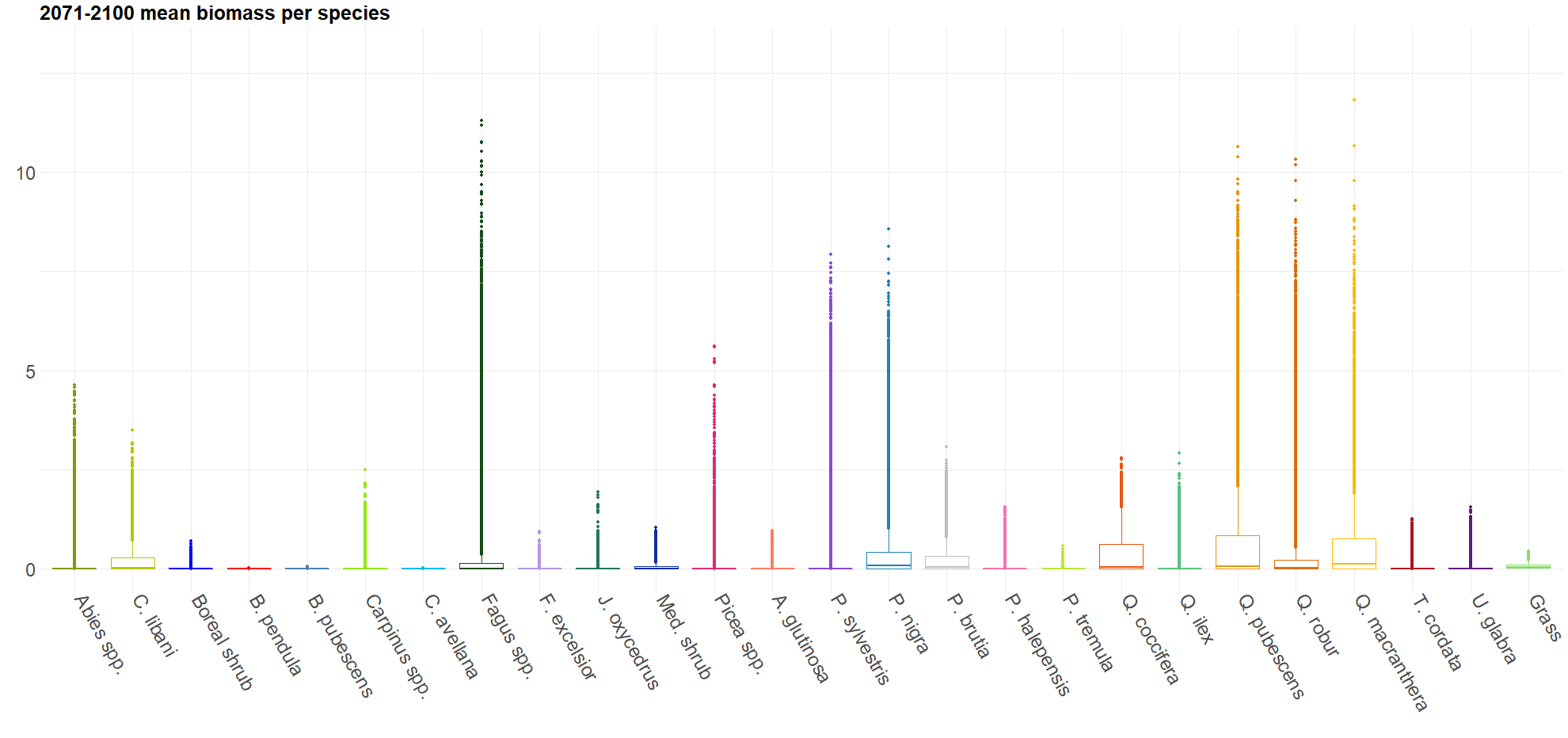


MPI-ESM1-2-HR

# 2


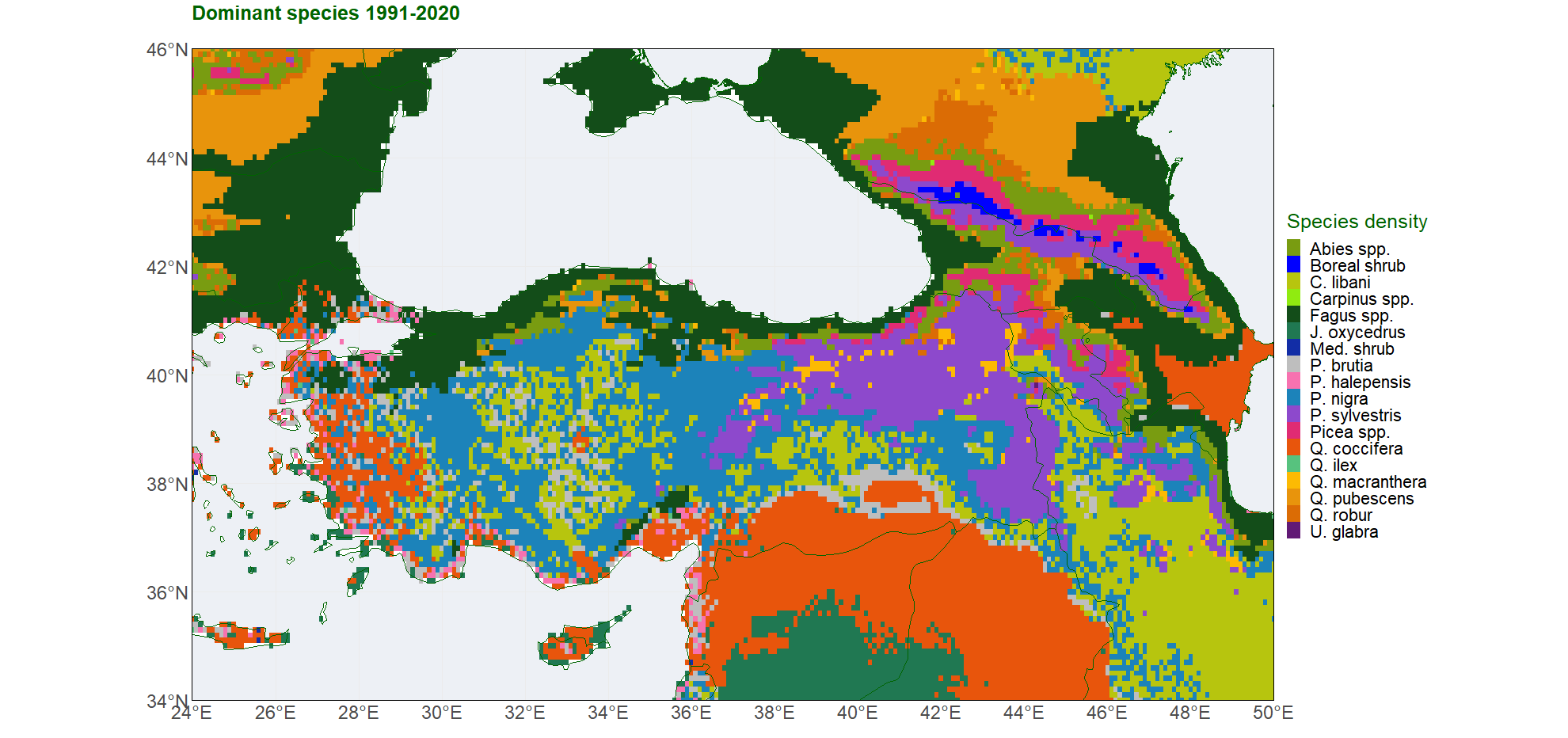

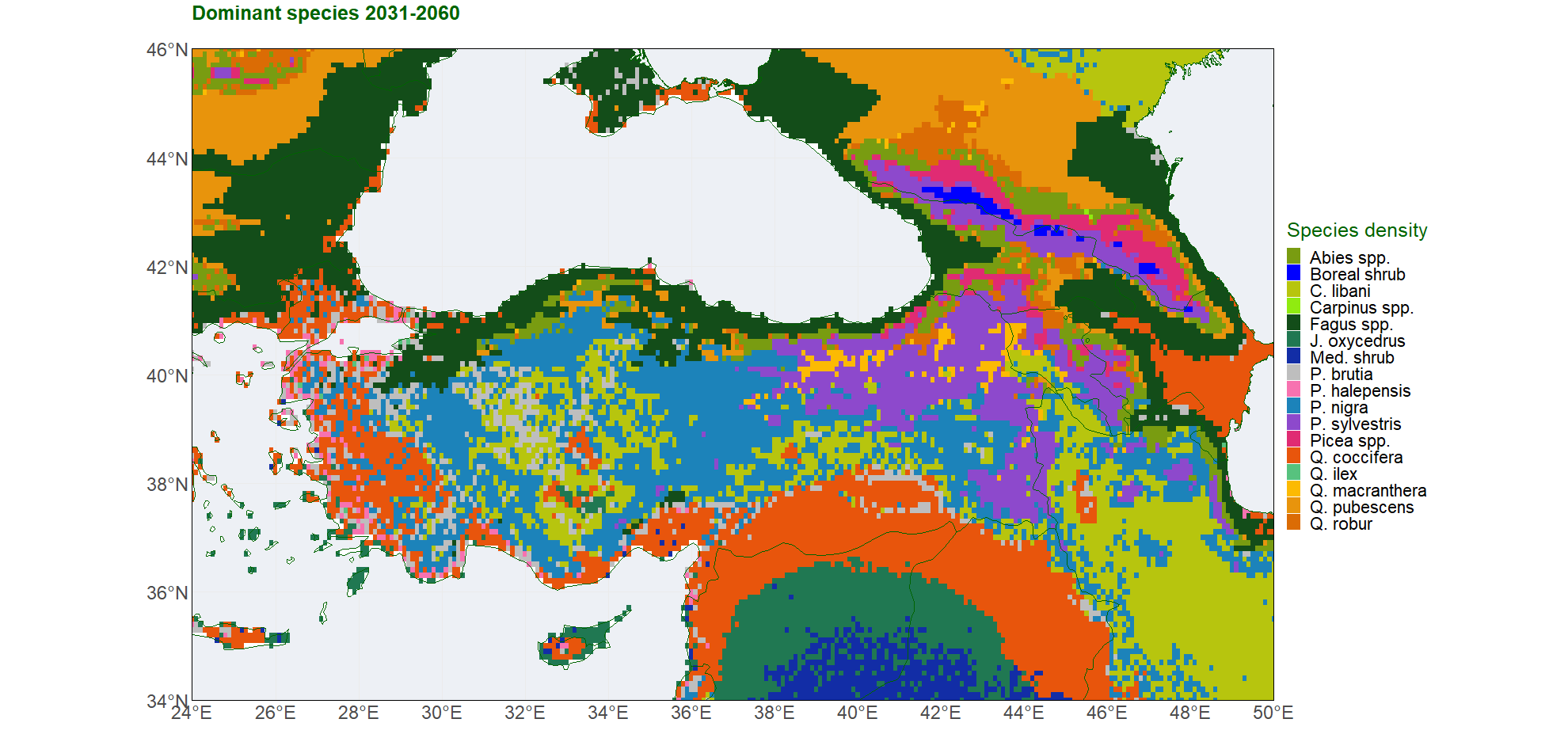

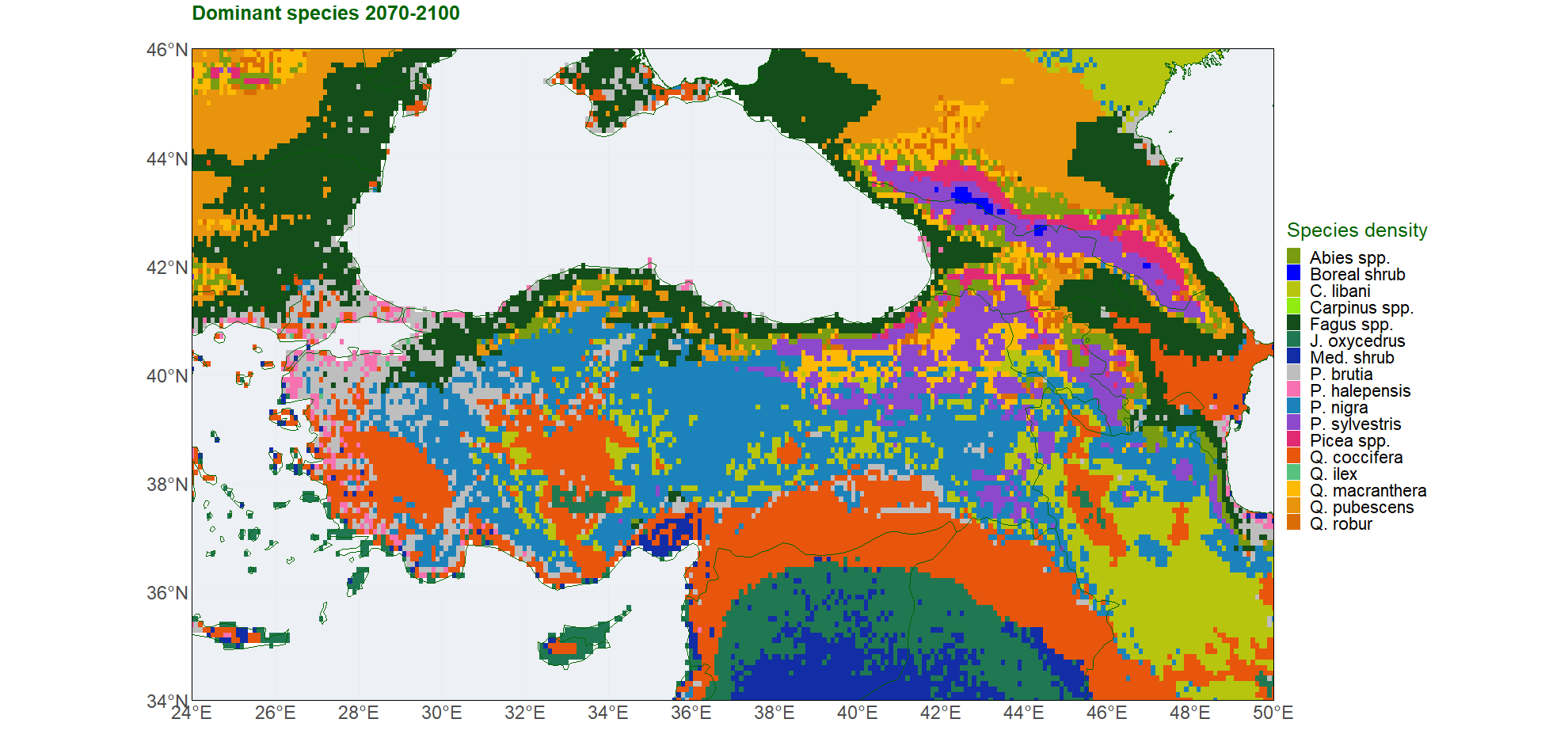

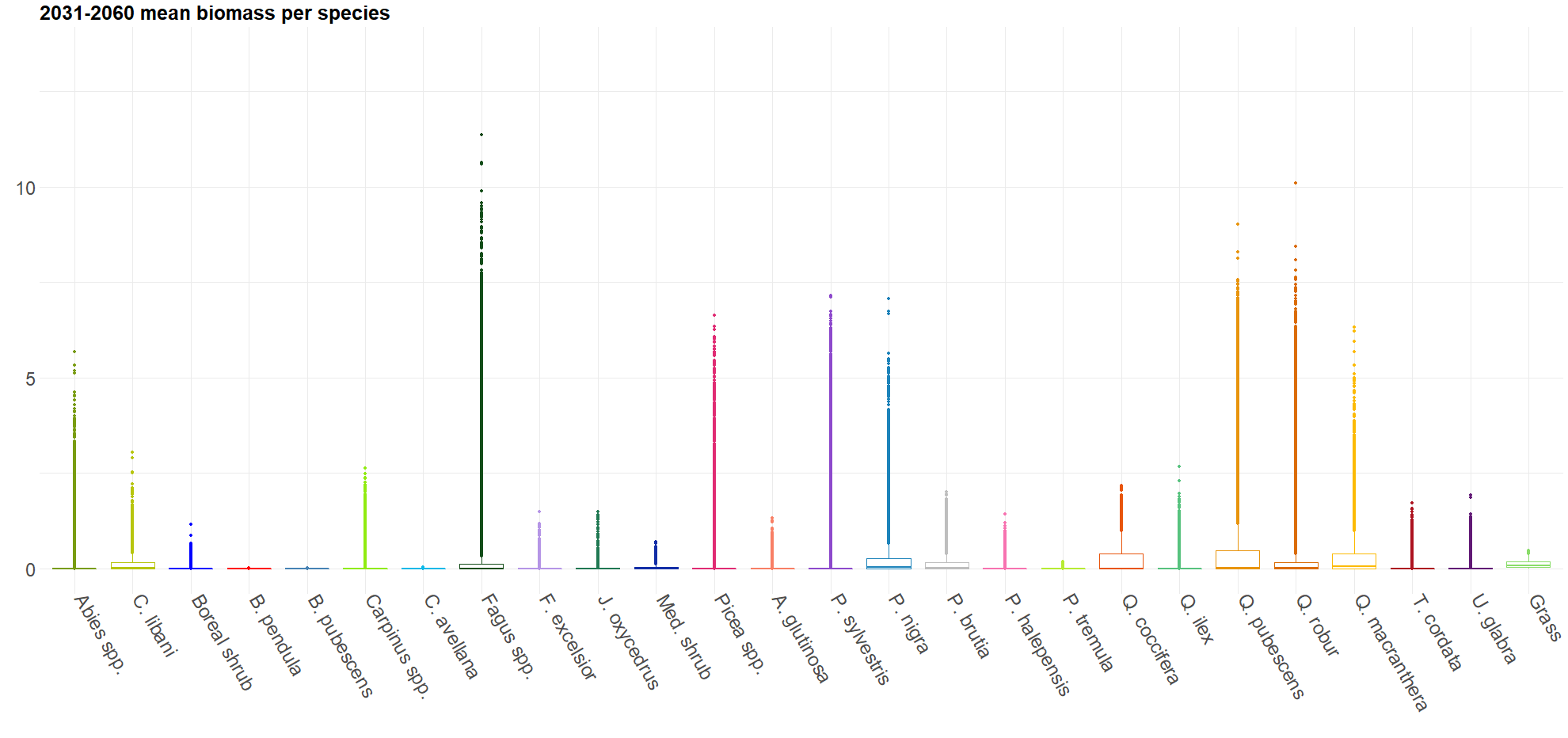

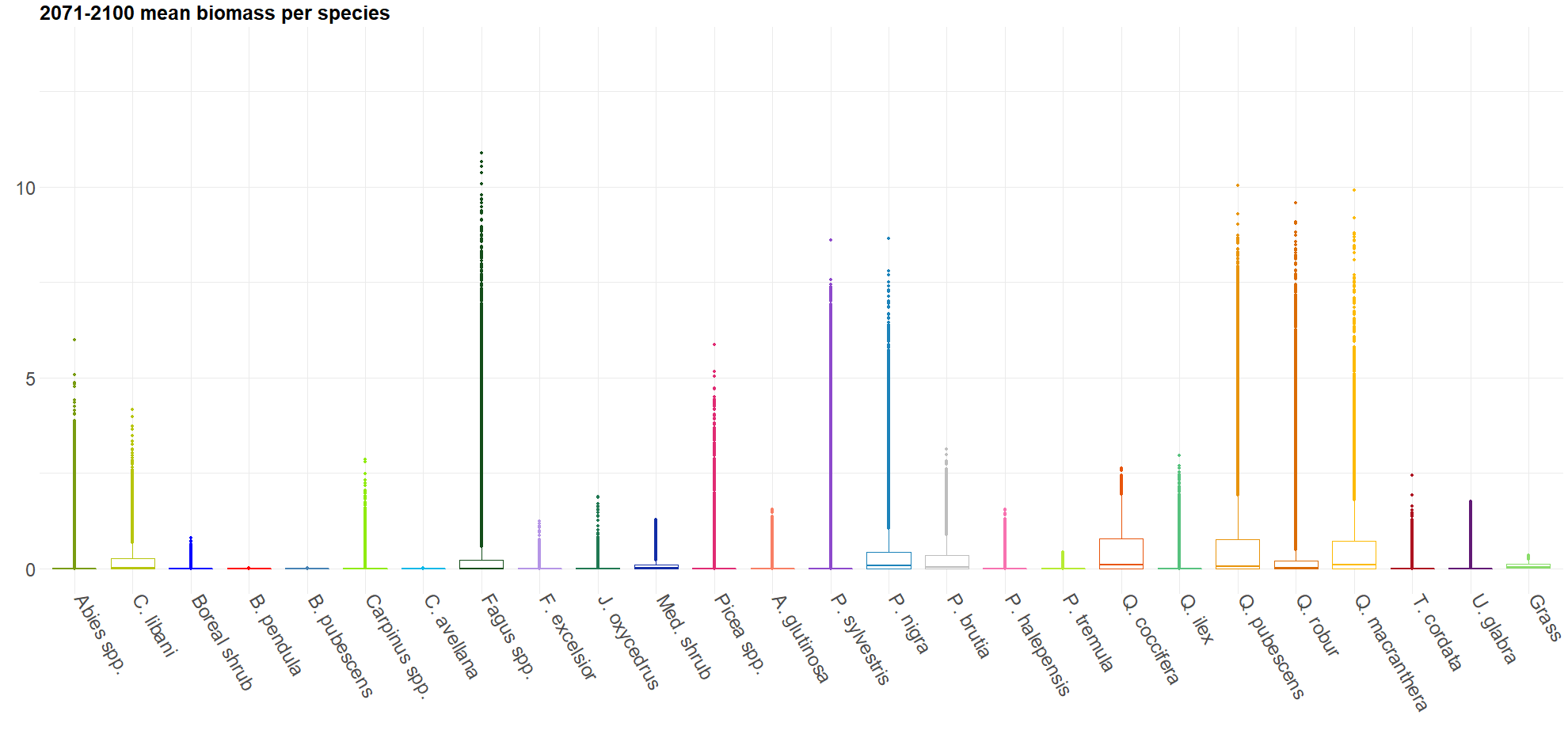


NorESM2-MM


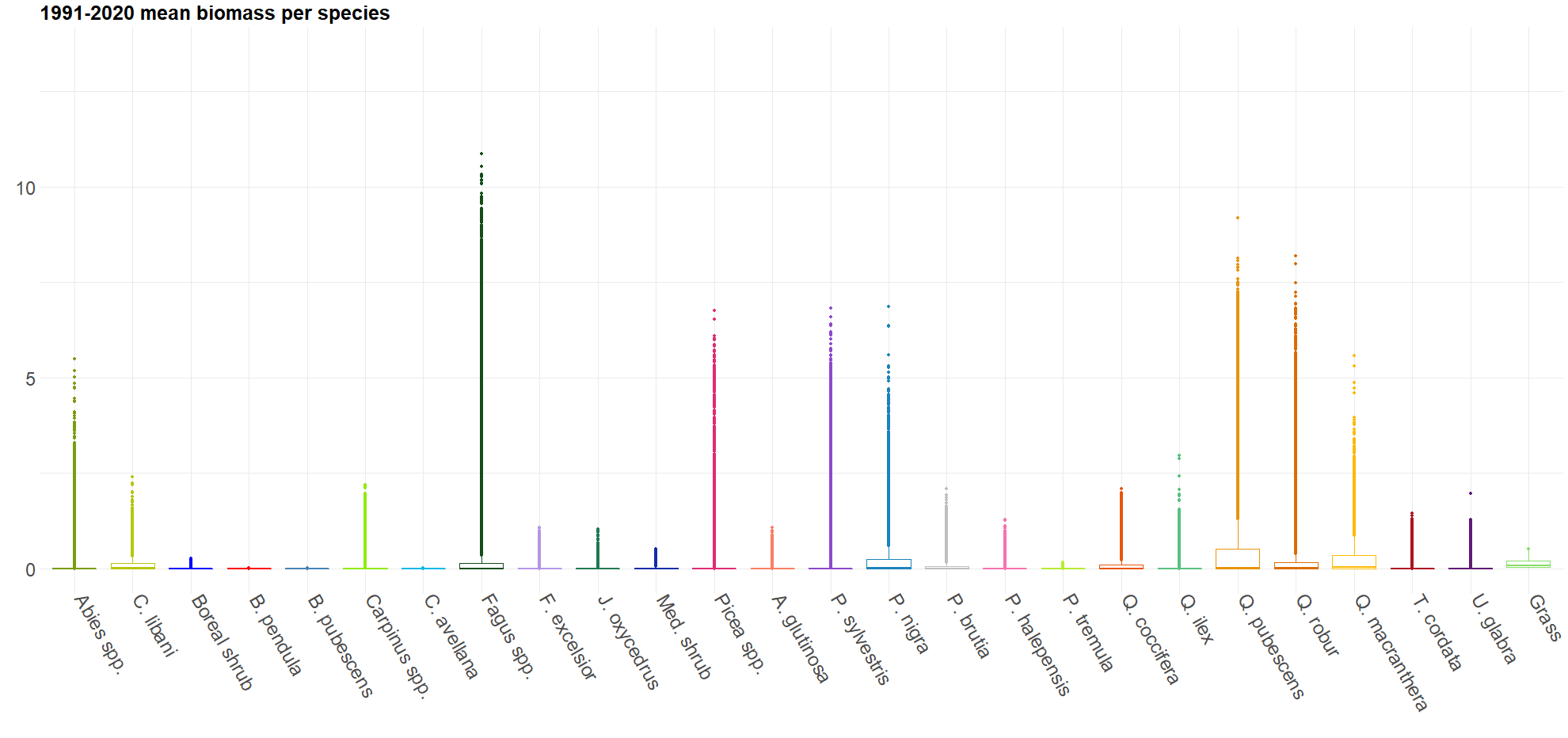


**Fig. S1** Maps of dominant species by density/stand calculated for individual GCMs for the period means of 1991-2020, 2031-2060 and 2070-2100 with boxplots of the taxa’s total biomasses (in kgC/m2) for the study area for the same periods.

# **
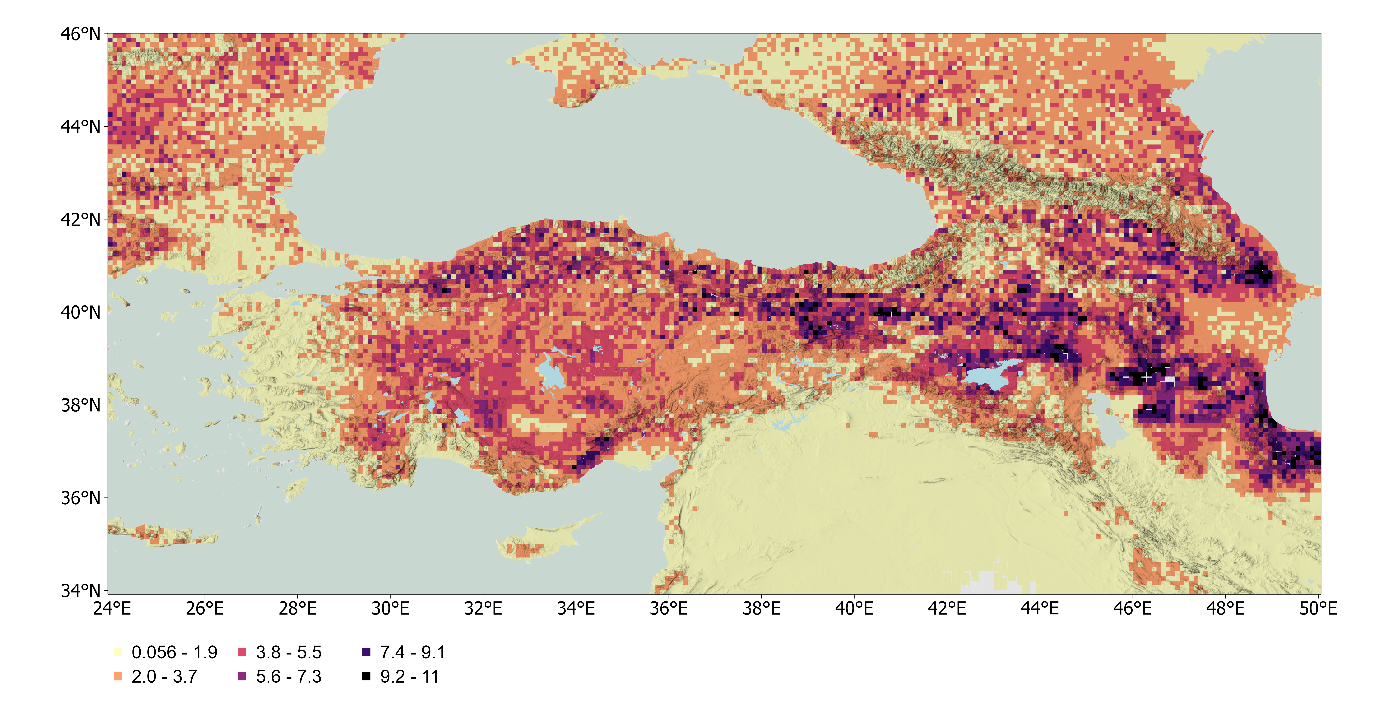

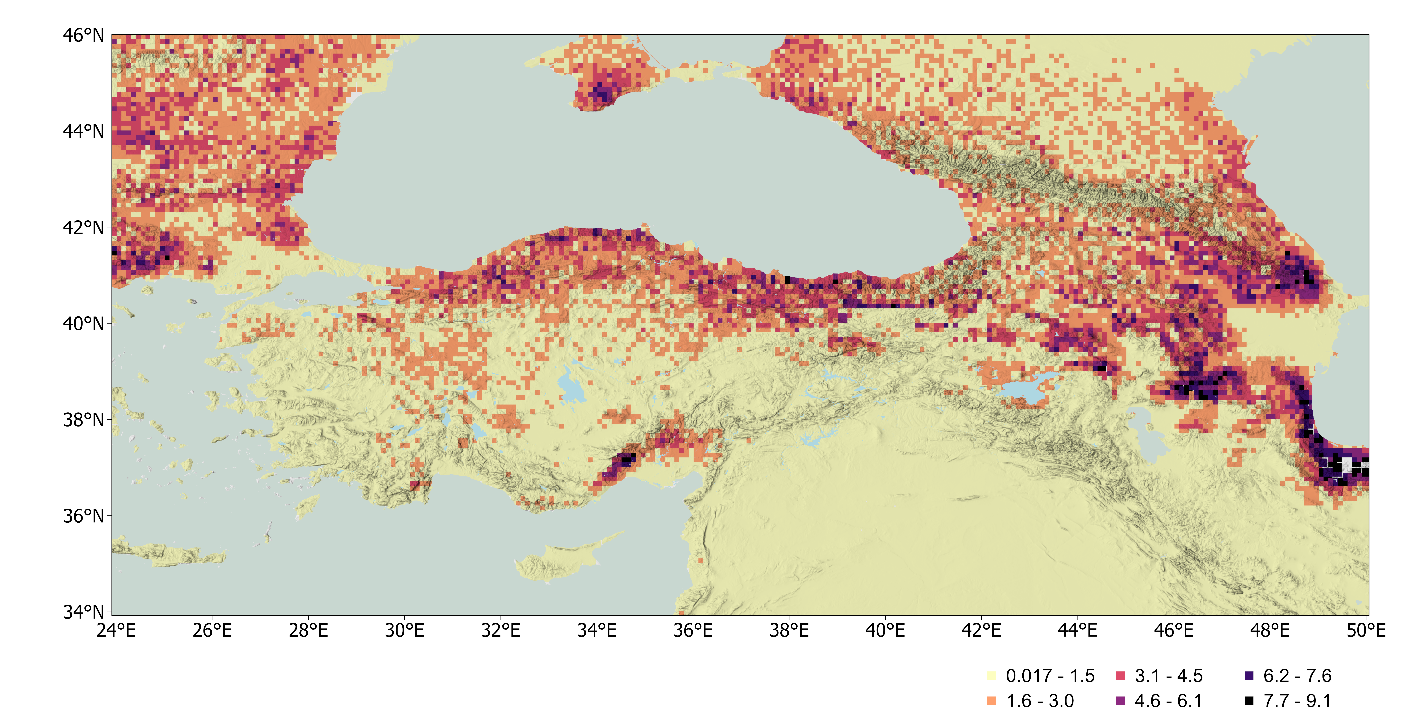

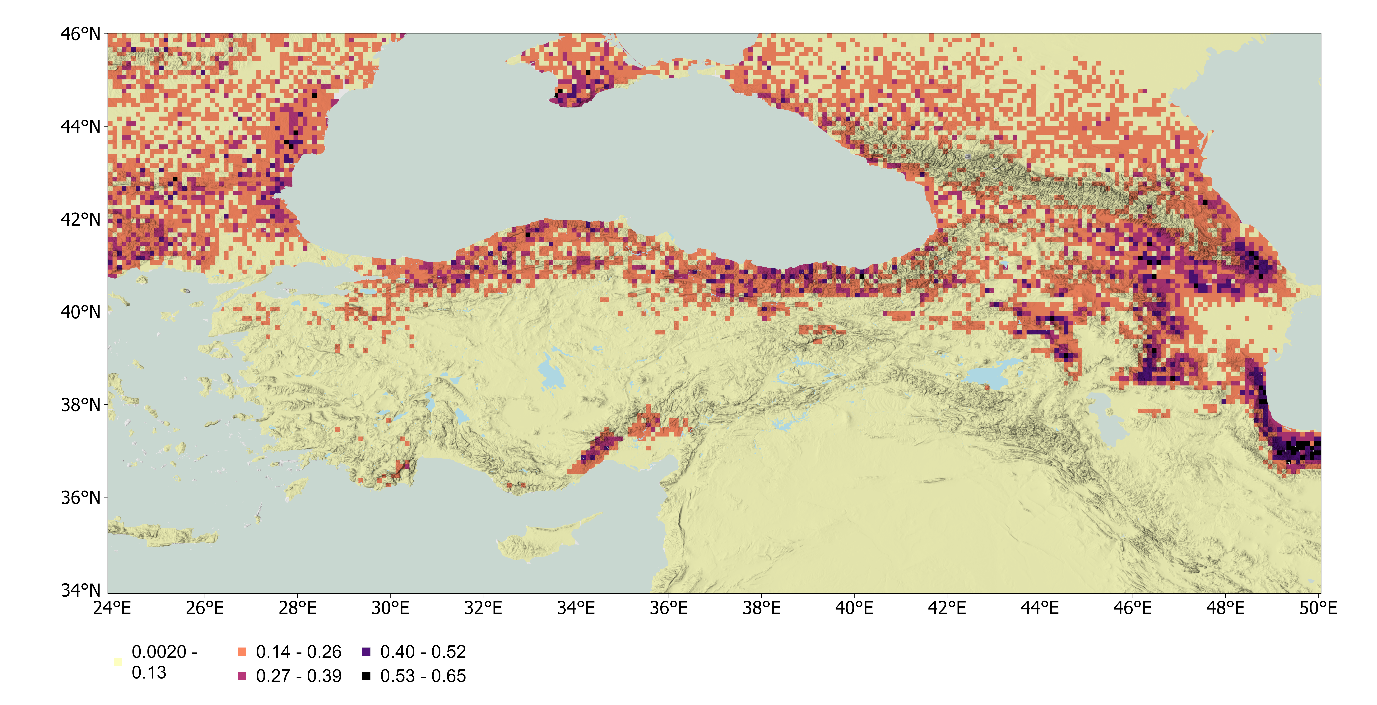
** **Fig. S2** Sum of absolute differences for the simulation results from the 5 GCMs used in the study: EC-Earth 3p-AHR; CMCC-ESM2; INM-CM5; MPI-ESM1-2-HR; NorESM2-MM, calculated for total woody biomass for (a) 1991-2020, (b) 2031-2060 and (c) 2071-2100

2031-2060

1991-2020

2071-2100

# a

# b

# c

#
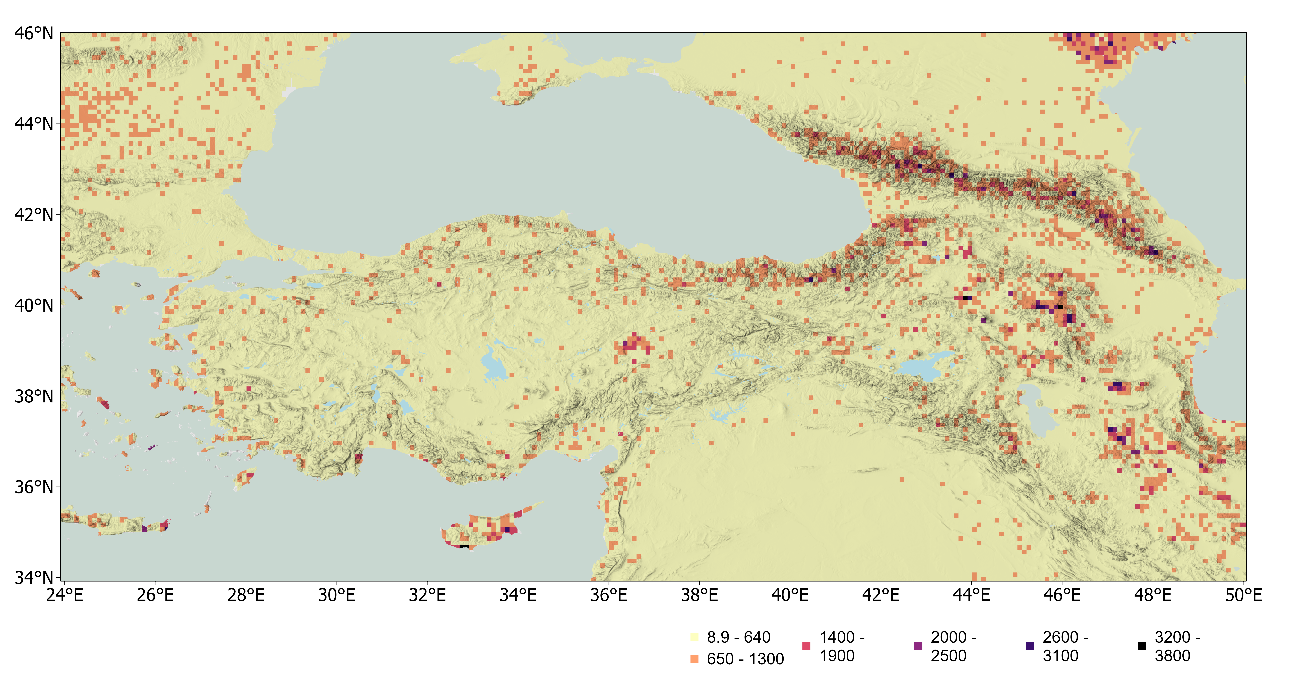

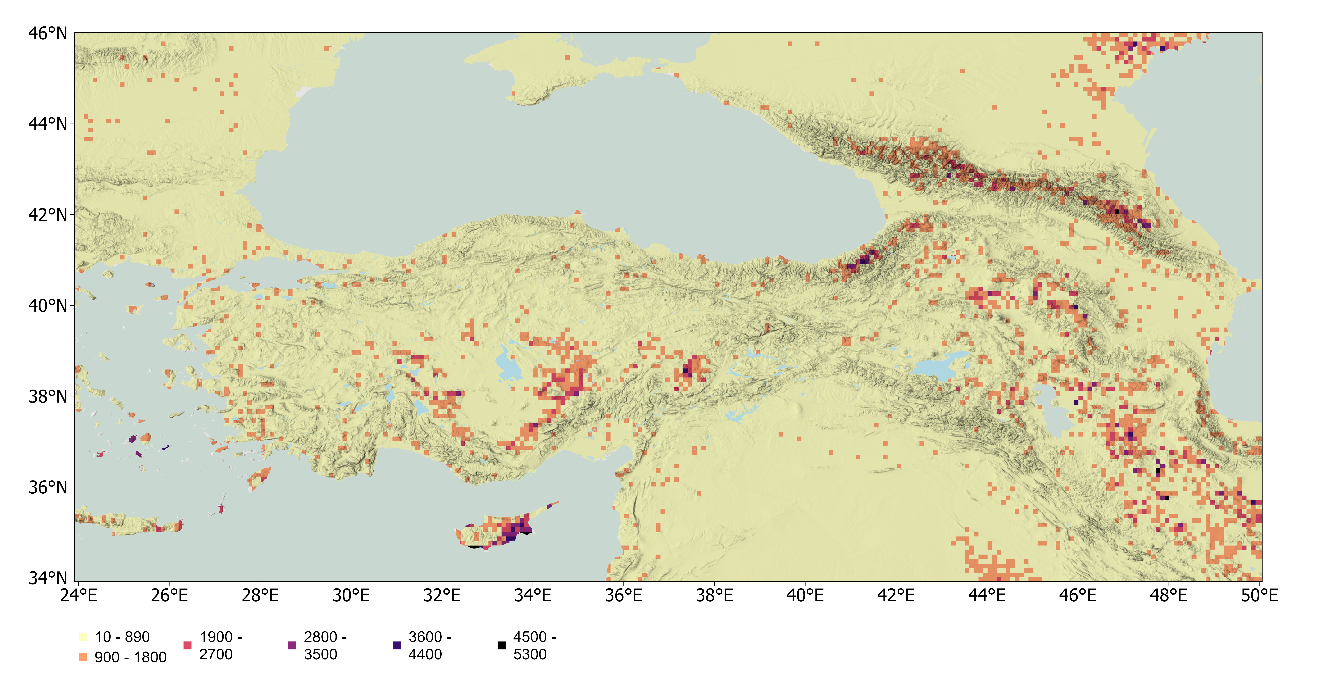

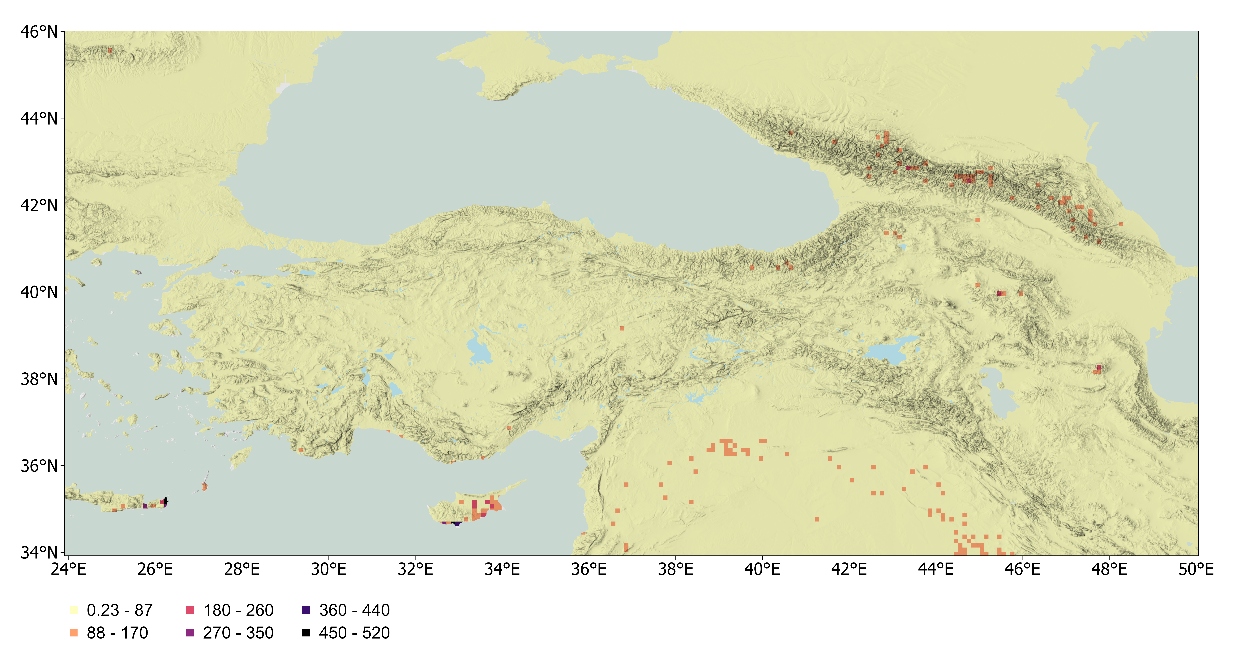
**Fig. S3** Sum of absolute differences for the simulation results from the 5 GCMs used in the study: EC-Earth 3p-HR; CMCC-ESM2; INM-CM5; MPI-ESM1-2-HR; NorESM2-MM, calculated for total woody density for (a) 1991-2020, (b) 2031-2060 and (c) 2071-2100

2071-2100

2031-2060

1991-2020

# c

# b

# a

**
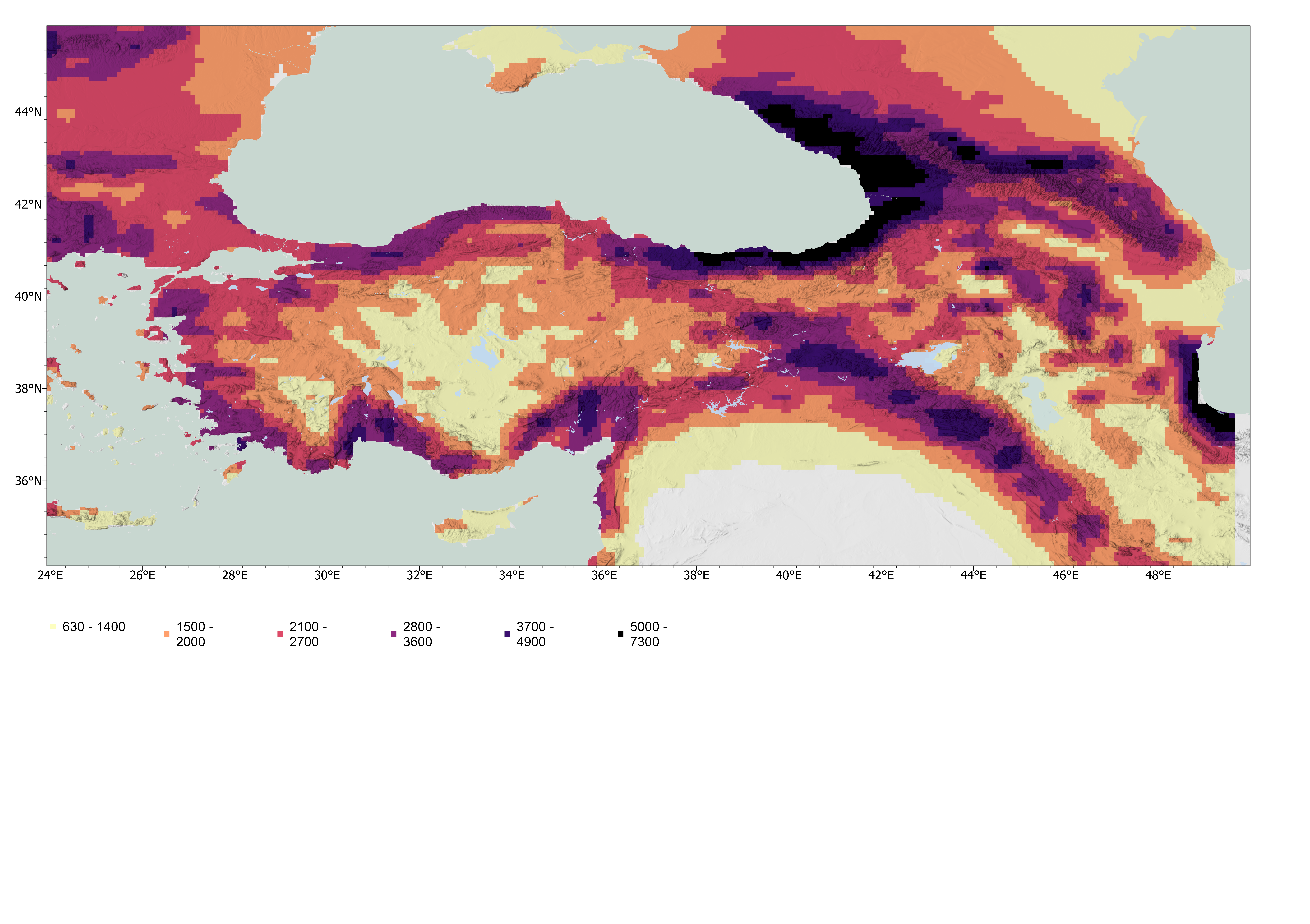

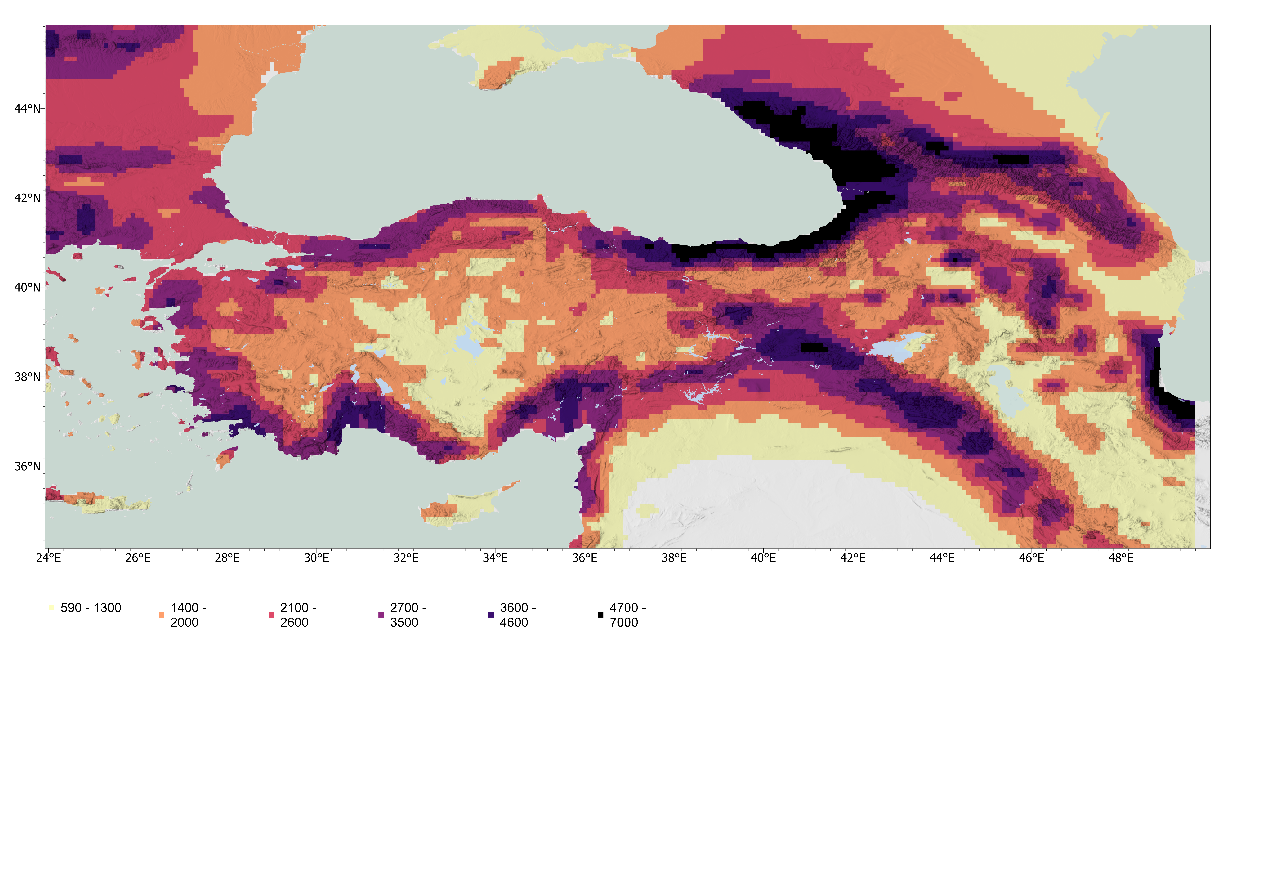

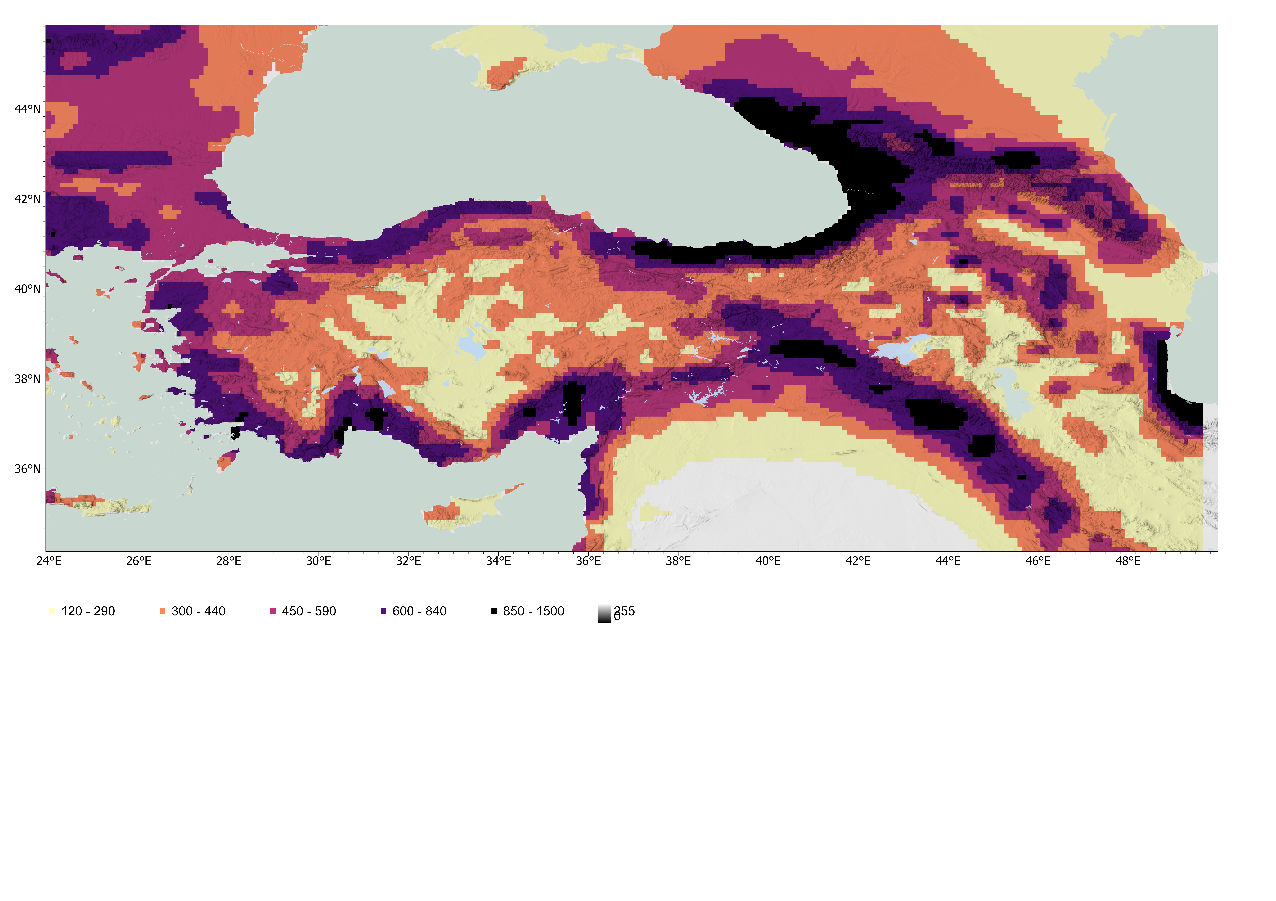
Fig. S4** Sum of absolute differences for total annual precipitation from the 5 GCMs used in the study: EC-Earth 3p-HR; CMCC-ESM2; INM-CM5; MPI-ESM1-2-HR; NorESM2-MM, calculated for (a) 1991-2020, (b) 2031-2060 and (c) 2071-2100

2071-2100

2031-2060

1991-2020

# c

# b

# a

**
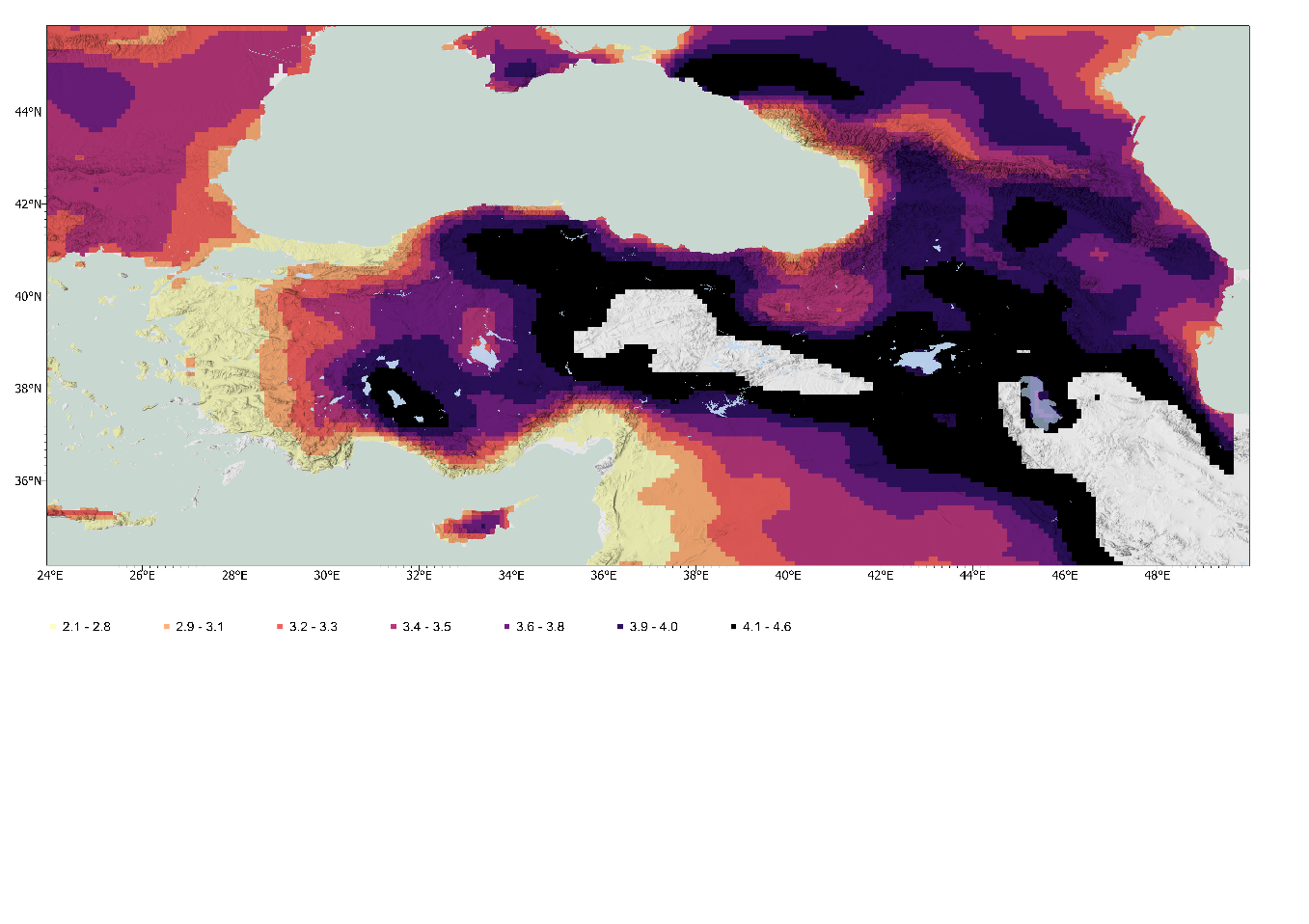

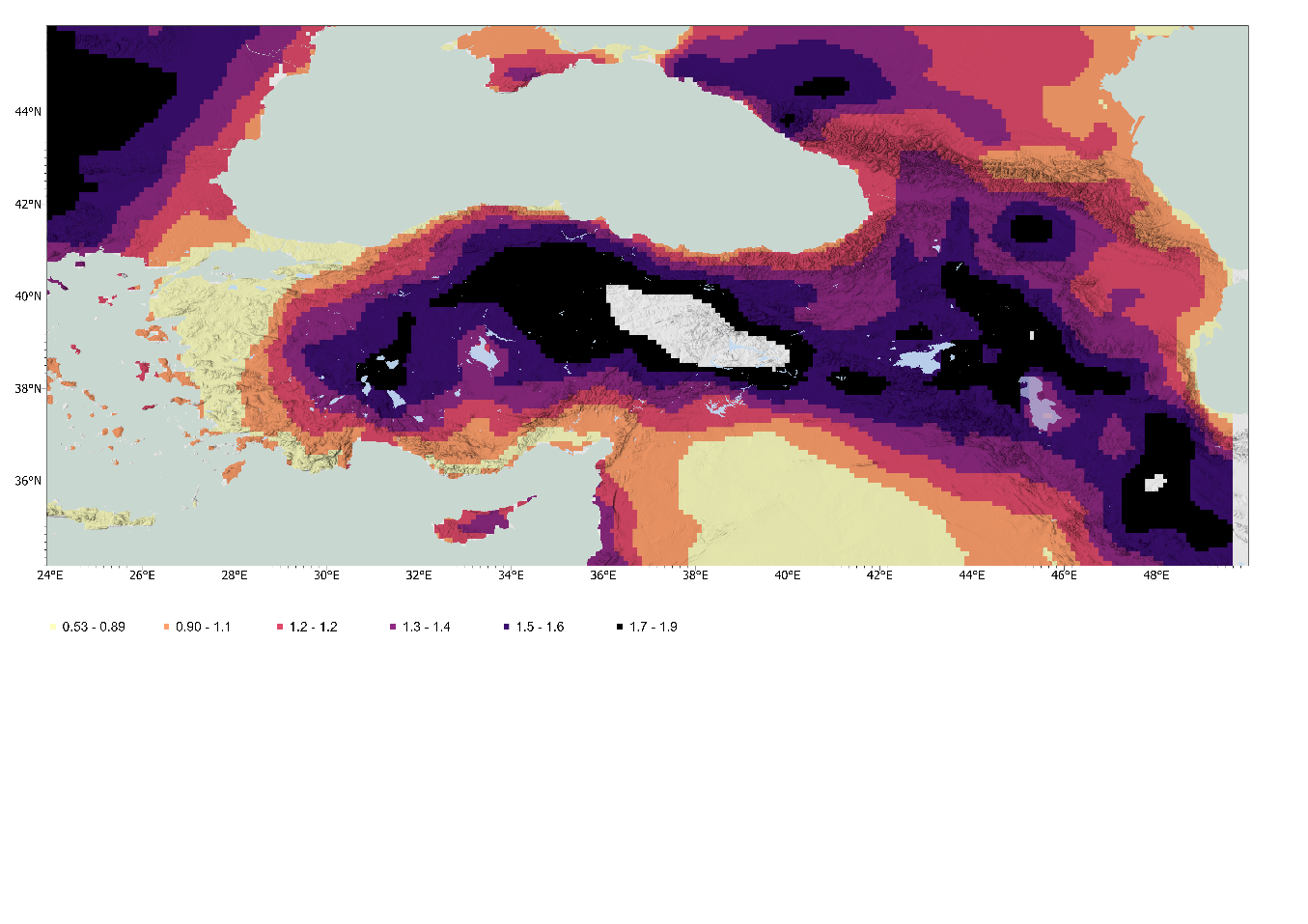

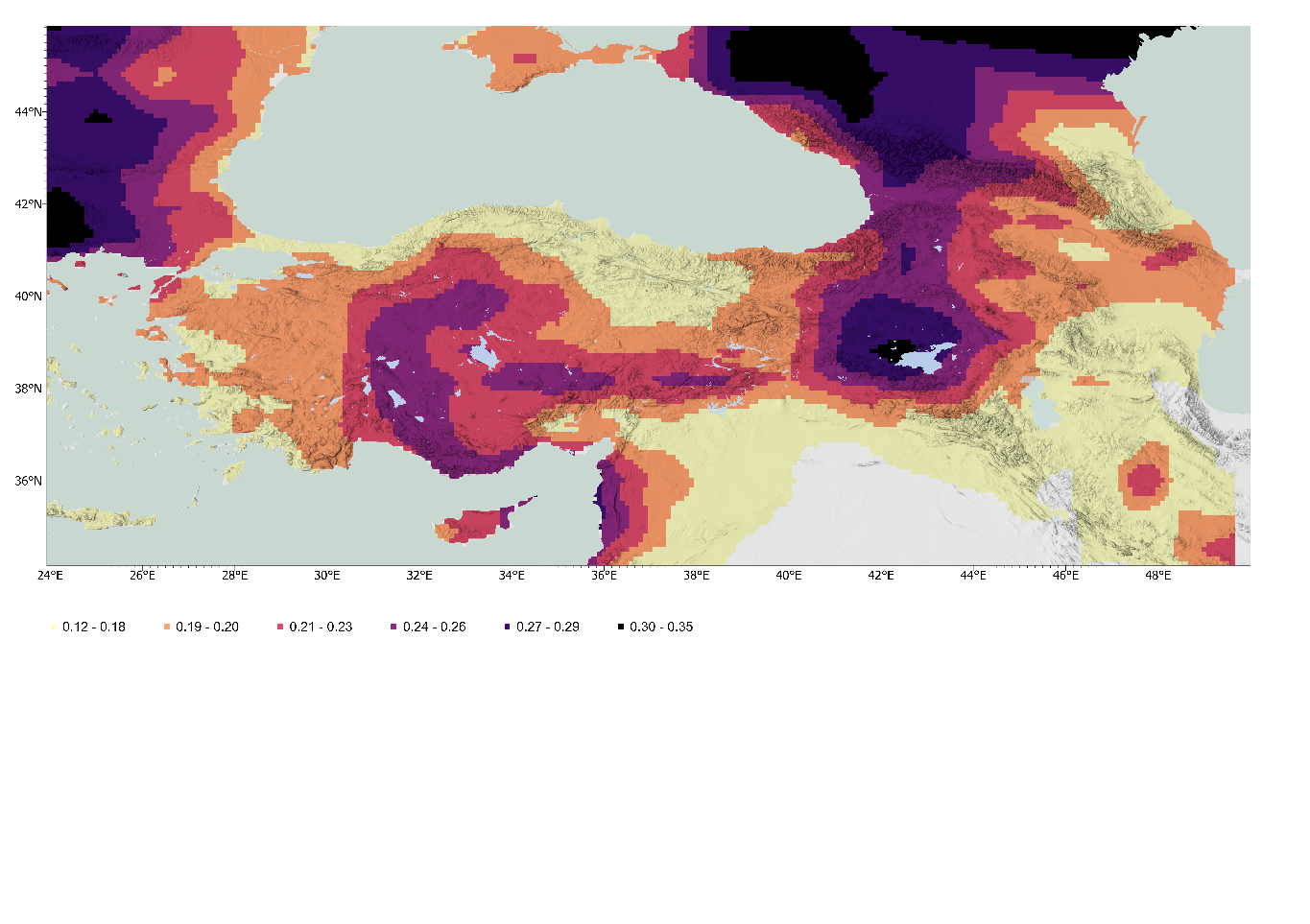
Fig. S5** Sum of absolute differences for annual mean temperature from the 5 GCMs used in the study: EC-Earth 3p-HR; CMCC-ESM2; INM-CM5; MPI-ESM1-2-HR; NorESM2-MM, calculated for (a) 1991-2020, (b) 2031-2060 and (c) 2071-2100

2071-2100

2031-2060

1991-2020

# c

# b

# a

**
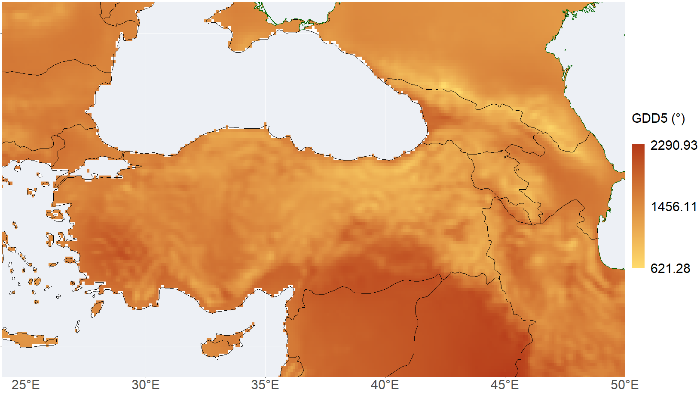
**
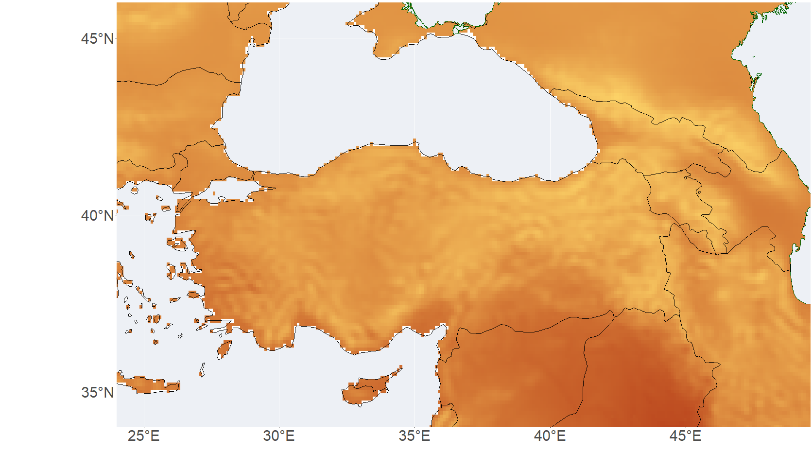

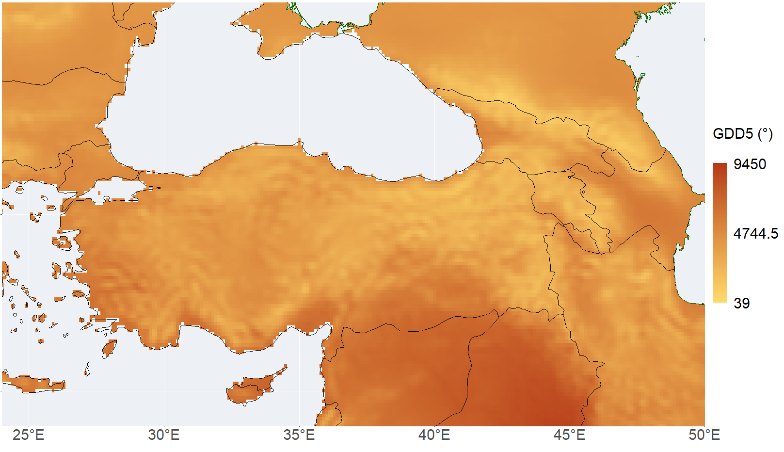

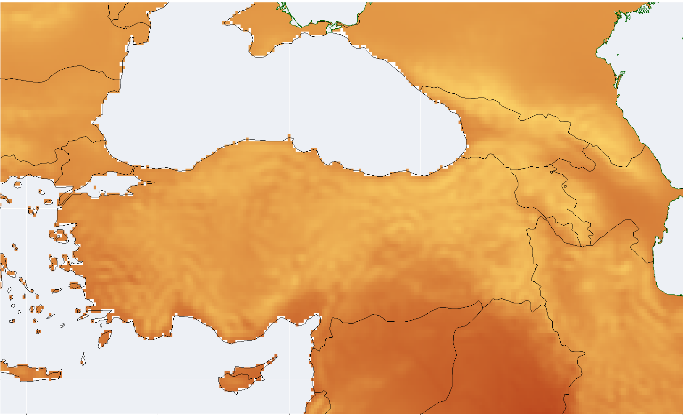

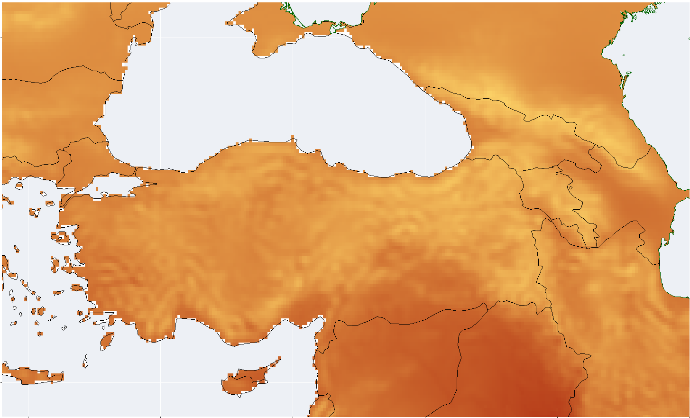

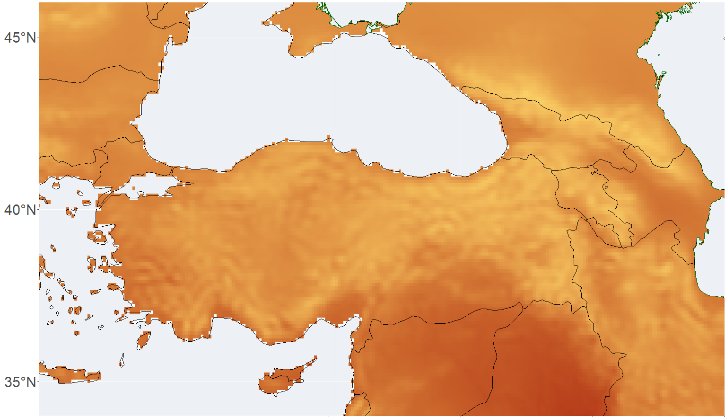
**
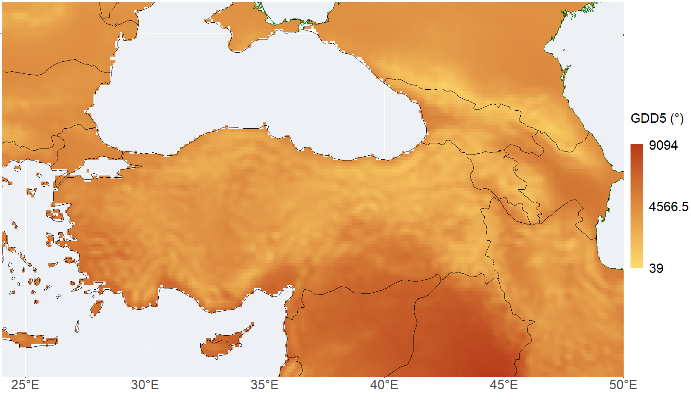

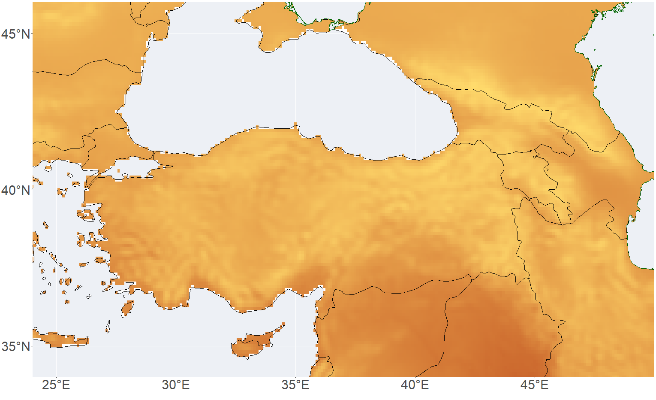
Fig. S6** GDD5min for the beginning of the simulation period; end of the simulation period for the ensemble median and the difference between (as in main text)

difference

NorESM2-MM

MPI-ESM1-2-HR

INM-CM5

EC-EARTH-3P-HR

CMCC-ESM2

2071-2100

1961-1990

**Fig. S7** Projected GDD5min for the end of the simulation period (2071-2100) for each GCM.
